# Induced Resistance Mechanism of Novel Curcumin Analogs Bearing a Quinazoline Moiety to Plant Virus

**DOI:** 10.3390/ijms19124065

**Published:** 2018-12-15

**Authors:** Limin Yin, Xiuhai Gan, Jing Shi, Ningning Zan, Awei Zhang, Xiaoli Ren, Miao Li, Dandan Xie, Deyu Hu, Baoan Song

**Affiliations:** State Key Laboratory Breeding Base of Green Pesticide and Agricultural Bioengineering/Key Laboratory of Green Pesticide and Agricultural Bioengineering, Ministry of Education, Guizhou University, Guiyang 550025, China; 18585062530@163.com (L.Y.); gxh200719@163.com (X.G.); sjxw99@163.com (J.S.); gs.nnzan17@gzu.edu.cn (N.Z.); ewzhang15@163.com (A.Z.); gs.xlren17@gzu.edu.cn (X.R.); gs.miaoli17@gzu.edu.cn (M.L.); xddxed@163.com (D.X.)

**Keywords:** tobacco mosaic virus, curcumin analogs, quinazoline moiety, protective activity, plant induced resistance

## Abstract

Plant immune activators can protect crops from plant virus pathogens by activating intrinsic immune mechanisms in plants and are widely used in agricultural production. In our previous work, we found that curcumin analogs exhibit excellent biological activity against plant viruses, especially protective activity. Inspired by these results, the active substructure of pentadienone and quinazoline were spliced to obtain curcumin analogs as potential exogenously induced resistant molecule. Bioassay results showed that compound **A13** exhibited excellent protective activity for tobacco to against Tobacco mosaic virus (TMV) at 500 μg/mL, with a value of 70.4 ± 2.6% compared with control treatments, which was better than that of the plant immune activator chitosan oligosaccharide (49.0 ± 5.9%). The protective activity is due to compound **A13** inducing tobacco resistance to TMV, which was related to defense-related enzymes, defense-related genes, and photosynthesis. This was confirmed by the up-regulated expression of proteins that mediate stress responses and oxidative phosphorylation.

## 1. Introduction

Tobacco mosaic virus (TMV) is a well-studied plant virus that causes massive crop loss. The prevention and treatment of TMV remain a problem worldwide [1]. Previous achievements in the prevention and treatment of plant virus diseases involved large doses of drugs, such as Ribavirin, Triacontanol, Benzo-(1,2,3)-thiadiazole-7-carbothiolic acid, which caused irreparable damage to the plants [2,3,4]. Therefore, based on crop health, the development of efficient, environmentally friendly antiviral agents is particularly important [5]. The relevant defense substances in plants that provide defense can resist viral invasion, inhibit viral proliferation in vivo, or confer self-protection. The defense process is extremely slow; however it can be hastened by plant immune activators, which can boost pattern-triggered immunity (PTI) resulting in enhanced resistance against pathogens [6,7,8]. Plant immune activators are agrochemicals that protect crops from pathogens [9]. This defense mechanism is similar to the innate immune system in animals [10]. So, plant activators, provides an effective method of crop protection [11]. The integration of plant immune activators into agricultural production practices can decrease both the volume and number of pesticides used and provide a solution to avoid environmental contamination [12].

A number of different types of compounds with stable and effective activities as plant immune activators have been explored so far. As one of the important plant immune activators, chitosan oligosaccharide (COS) can induce disease resistance in plants and enhances a plant’s defense against pathogens [13,14,15]. COS is also widely used to induce resistance induction in plants for the prevention and treatment of TMV [16]. However, the widespread use of this antiviral agent in field trials is limited by its short-term control effect [17,18]. Therefore, new plant immune activators should be discovered to use in the field.

**Figure 1:**
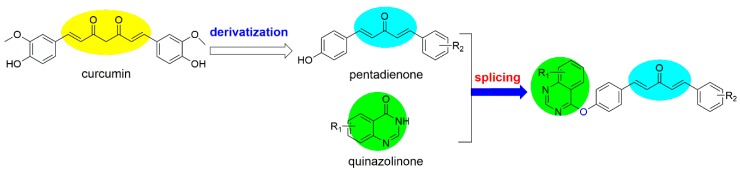
Design of the target compounds. The yellow circle represents the modifiable structure part of curcumin, the blue circles represent the structure part of pentadienone, and the green circles represent the structure part of quinazolinone.

It is well known that most of plant immune activators are metabolic products of organisms or derive from the metabolic products. So, finding a plant immune activator that is based on a natural product is an effective way to develop new drugs. In our previous study, we found that *trans*-ferulic acid derivatives, which are α,β-unsaturated carbonyl compounds, display a favorable inhibitory activity against TMV, and they can enhance the disease resistance of tobacco by increasing the activity of defense enzymes and promoting photosynthesis [19]. Curcumin analogs, which are also α,β-unsaturated carbonyl compounds, exhibit excellent biological activity against plant viruses [20,21,22,23]. However, the mechanism by which resistance is induced in plants has not been studied yet. In addition, quinazoline derivatives show a favorable inhibitory activity against plant viruses, and some studies revealed that they can induce resistance in tobacco [20,21,22,23,24]. Searching for novel plant immune activators, we designed and synthesized a series of novel curcumin analogs bearing the quinazoline moiety (Figure 1). Their inhibitory activity against TMV was evaluated. Then, the mechanism by which of **A13** induces resistance was analyzed, including the defense-related enzyme activity, chlorophyll content, defense genes, and differentially expressed proteins (DEPs). The results show that compound **A13** can effectively enhance the resistance of tobacco, and it can be used as a plant immune activator.

## 2. Results

### 2.1. Synthesis

The target compounds **A1**–**A20** were synthesized with the intermediates 4-chloroquinazoline and (1*E*, 4*E*)-1-(substituted-phenyl)-5-(4-hydroxyphenyl)-penta-1,4-dien-3-one. The intermediate 4-chloroquinazoline was synthesized as described in a previous report [22]. The intermediate (1*E*, 4*E*)-1-(substituted-phenyl)-5-(4-hydroxyphenyl)-penta-1,4-dien-3-one was synthesized by using a previously described method [25]. The target compounds were confirmed by 1H NMR, 13C NMR, 19F NMR, and HRMS, and synthesized as detailed in the Supporting data-1. 

### 2.2. Antiviral Biological Assay

The antiviral activities of the target compounds **A1**–**A20** against TMV were tested by using the half-leaf method in vivo [26]. The plant activator COS was used as the control. The results are presented in Table 1. The protective activity of compounds **A3**, **A8**, **A9**, **A15**, and **A20** against TMV was comparable with that of COS (49.0 ± 5.9%) at 500 μg/mL, with values of 50.6 ± 2.8%, 47.3 ± 9.3%, 48.8 ± 3.3%, 47.6 ± 9.8%, and 53.8 ± 3.0%, respectively. Interestingly, compounds **A12** and **A13** exhibited superior protective activity against TMV over the control agent COS, with values of 66.5 ± 3.0%, and 70.4 ± 2.6%, respectively. Meanwhile, the curative activity of compounds **A1**, **A3**, **A11**, **A12**, **A13**, and **A14** against TMV was comparable to that of COS (34.1 ± 8.9%) at 500 μg/mL, with values of 55.8 ± 3.7%, 58.1 ± 10.0%, 49.9 ± 9.6%, 42.0 ± 5.5%, 42.9 ± 2.2%, and 50.8 ± 7.7%, respectively.

### 2.3. The Mechanism by Which Compound A13 Induces Resistance

#### 2.3.1. Physiological and Biochemical Analysis

*N. tabacum* cv. K326 with the same growth at the six-leaf stage was selected, and compound **A13** was smeared over whole leaves at 500 μg/mL. Solvents (1% tween) and COS were used as the negative (CK) and positive controls (COS), respectively. Tobacco leaves were inoculated with TMV after 24 h, and they were placed, and cultured, in a greenhouse. Four treatments were prepared: CK, CK + TMV, COS + TMV, and A13 + TMV. Tissues were collected on 0, 1, 2, and 3 day after the tobacco leaves were inoculated with TMV for assays on defensive enzyme activities and chlorophyll content. The tissue samples were obtained in triplicate.

##### The Effect on Defensive Enzyme Activity

**Figure 2:**
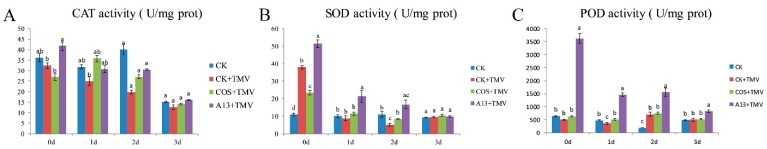
The effect of compound **A13** on CAT (**A**), SOD (**B**), and POD (**C**) activity in tobacco leaves. Bars indicate the mean of three replicates with the standard deviations. Different letters on the bars indicate statistically significant differences in average values by one-way ANOVA (*p* < 0.05).

The changes of various defensive enzyme of tobacco treated with **A13** were investigated and presented in Figure 2. The catalase (CAT) (Figure 2A) activity in the **A13** + TMV treatment group decrease from day 0 to day 3, reaching its minimum on day 3. On day 3, the CAT activity was higher in the **A13** + TMV treatment group than in the other treatment groups. The results showed that compound **A13** can effectively increase the CAT activity in plants. The superoxide dismutase (SOD) (Figure 2B) activity in the **A13** + TMV treatment group was 1.35, 2.18 and 4.67 times higher than that in the COS + TMV, CK + TMV, and CK groups from day 0 to day 3, respectively, and reached its maximum on day 0. The results suggest that compound **A13** can rapidly increase the SOD activity. The phenylalanine ammonia lyase (POD) (Figure 2C) activity in the **A13** + TMV treatment group was higher than that in the CK, CK + TMV, and **COS** + TMV groups from day 0 to day 3, reaching the highest values on day 0. Compound **A13**’s ability to enhance the POD activity in plants, is remarkable, and is superior to that of the COS. The results of the defense enzyme activity test show that **A13** enhances disease tobacco resistance to TMV by increasing its defensive enzyme activity.

##### The Effect on Chlorophyll Contents

**Figure 3:**
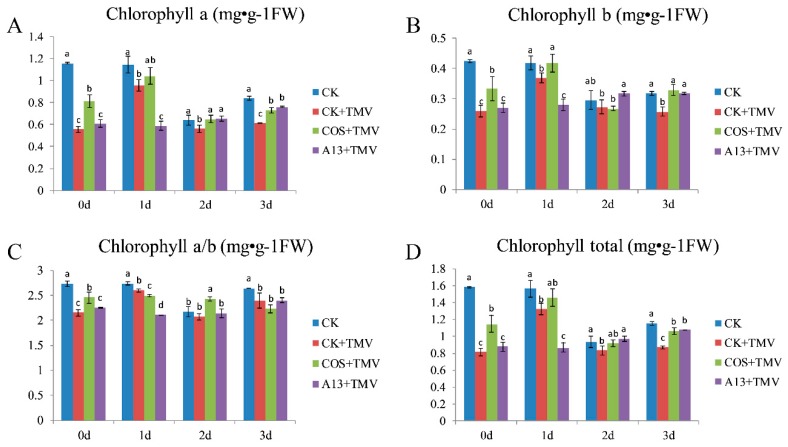
The effects of compound **A13** on the Ca (**A**), Cb (**B**), chlorophyll a/b (**C**), and Ct (**D**) content in tobacco leaves. Bars indicate the mean of three replicates with the standard deviations. Different letters on the bars indicate statistically significant differences in average values by one-way ANOVA (*p* < 0.05).

To study the effect of **A13** on chlorophyll contents, we studied the changes in chlorophyll content (Figure 3), including chlorophyll a (Ca, Figure 3A), chlorophyll b (Cb, Figure 3B), chlorophyll a/b (Figure 3C), and total chlorophyll content (Ct, Figure 3D), in tobacco plants after inoculation with TMV. After the tobacco host was infected with TMV, the Ca, Cb, and Ct in the **A13** + TMV treatment groups showed minimal changes from day 0 and day 1 and then slightly increased on day 2. After the tobacco was infected with TMV, the Ca, Cb, and Ct contents decreased relative to the healthy blank group, however these parameters were higher in the **A13** treatment group than in the CK + TMV treatment group from day 2 to day 3. It is showed that compound **A13** has little effect on the chlorophyll content compared with CK.

##### The Effect on Defense-Related Genes

**Figure 4:**
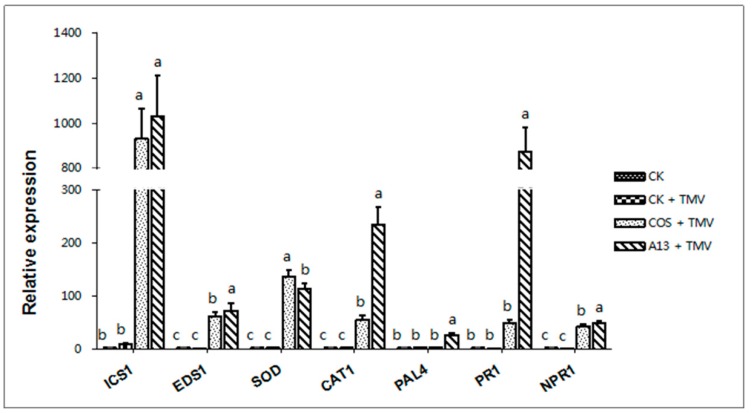
The expression of the defense-related genes was evaluated by RT-qPCR. The relative expression of the defense-related genes, including *ICS1*, *EDS1*, *SOD*, *CAT1*, *PAL4*, *PR1*, and *NPR1,* was highly up-regulated by the **A13** + TMV treatment on day 3. Bars indicate the mean of three replicates with the standard deviations. Different letters on the bars indicate statistically significant differences in average values by one-way ANOVA (*p* < 0.05).

As shown in Figure 4, the defense-related genes, including isochorismate synthase 1 (*ICS1*), enhanced disease susceptibility 1 (*EDS1*), superoxide dismutase (*SOD*), catalase 1 (*CAT1*), phenylalanine ammonia lyase 4 (*PAL4*), pathogenesis-related genes 1 *(PR1*), and nonexpressor of pathogenesis-related genes 1 (*NPR1*), in the **A13** + TMV treatment group were remarkably up-regulated on day 3. Specifically, the expression levels of *ICS1*, *CAT1*, and *PR1* in the **A13** + TMV treatment group were 1034.70, 876.13, and 876.13 times higher than those in the CK treatment group, respectively. The results show that compound **A13** can effectively enhance the activity of the defense enzymes in plants. Notably, the expression levels of *PR1* and *CAT1* in the **A13 +** TMV treatment group were 17.44 and 4.15 times higher than those in the COS + TMV treatment group, respectively. Furthermore, the expression levels of SOD in the **A13** + TMV treatment group was lower than that in the COS + TMV treatment group. The results showed that the compound **A13** can induce the expression of defense-related genes in tobacco, thus achieving antiviral effect.

#### 2.3.2. The Proteomics Analysis of Tobacco’s Response to **A13**

##### The DEP Analysis

**Figure 5:**
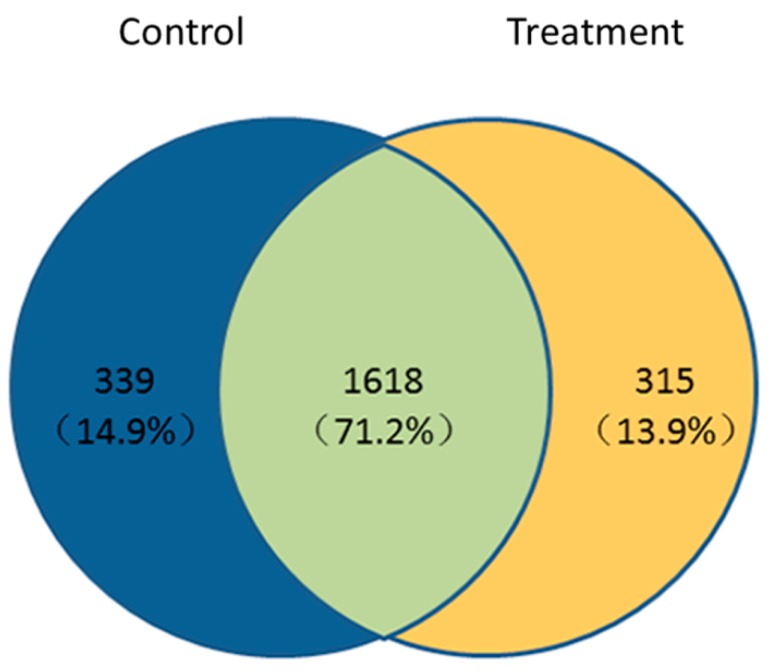
The changed in the proteome distribution between the control (CK + TMV) and treatment (**A13** + TMV) groups, the diagram shows both unique and shared proteins. Blue indicates DEPs were identified in the control, orange indicates DEPs were identified in the treatment, and green indicates DEPs were identified in both treatment groups.

**Figure 6:**
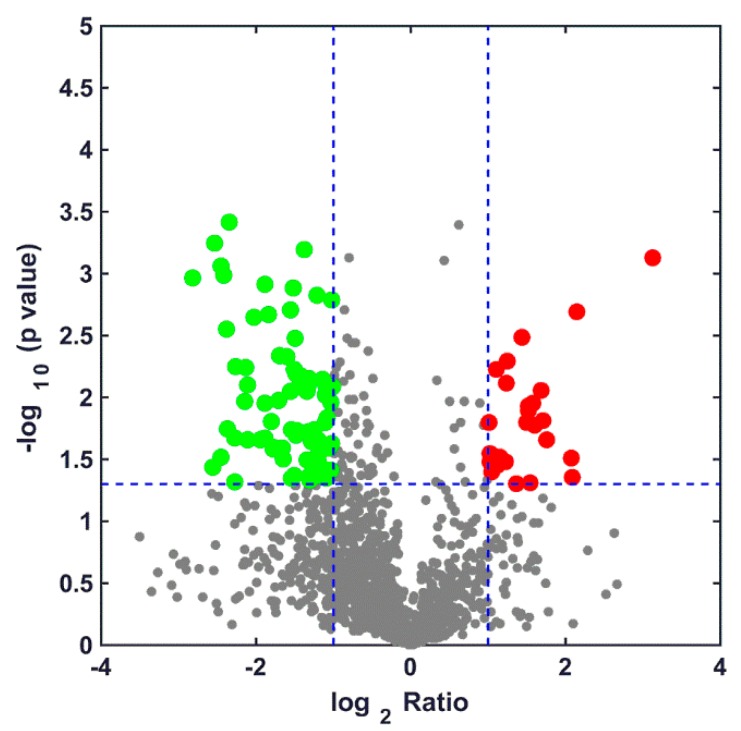
The numbers of identified proteins showing up-regulation and down-regulation in the control and treatment groups. The red spots represent up-regulate proteins, the blue dots down-regulated proteins, and the grey spots represent unchanged proteins in both treatment groups. The Log_2_ Ratio indicates that the ratio between the treatment groups and the control groups takes a natural logarithm. The longitudinal coordinates indicate the magnitude of differences in the protein level. Fold change > 2, *p* < 0.05.

To study the effect of the **A13** treatment on the proteins in the TMV-inoculated tobacco, we detected and identified total protein samples by using label-free LC−MS/MS on day 3. We used the iBAQ algorithm of the label-free protein spectrum technique to quantify the protein in tobacco leaves from the **A13** + TMV treatment and control groups. As shown in Figure 5, we identified 1933 and 1957 proteins in the **A13** + TMV and CK + TMV treatment groups, respectively (Table S1). The volcano plot in Figure 6 shows the DEP levels. A total of 1618 (71.2%) proteins was identified in both treatment groups, and 315 (13.9%) and 339 (14.9%) DEPs were identified in TMV + **A13** and CK + TMV, respectively (Table S1). Among the DEPs, 26 were upregulated and 77 were downregulated in the TMV + **A13** treatment group (fold change > 2, *p* < 0.05).

##### Differential Protein Gene Ontology (GO) Slim Analysis

**Figure 7:**
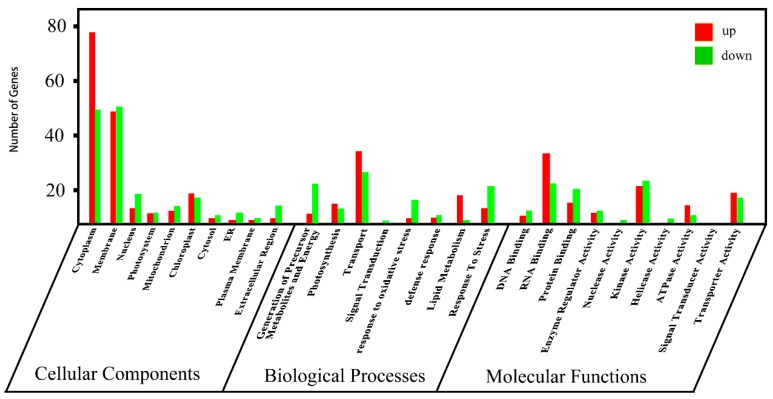
Cellular components, biological processes, and molecular functions involving DEPs in **A13** + TMV versus CK + TMV.

Figure 7 shows the results of a GO slim analysis to compare different **A13**-induced proteins (*p* ≤ 0.05). The GO-annotated map of **A13** + TMV versus CK + TMV shows that the DEPs involved in cellular components (Figure 7) were significantly enriched in 10 GO terms, including membrane, cytoplasm, photosystem, mitochondrion, and chloroplast. The DEPs in the biological processes (Figure 7) were remarkably enriched in eight GO-terms, mainly including photosynthesis, transport, lipid metabolism, stress response, defense response, and response to oxidative stress. The DEPs involved in molecular functions (Figure 7) were significantly enriched in nine GO terms, including RNA binding, DNA binding, protein binding, kinase activity, transporter activity, and ATPase activity. Among the 654 DEPs, 26 DEPs were upregulated, including glutamate dehydrogenase, defensin, cytochrome, photosystem I P700 chlorophyll and apoprotein A1, PABP, ATP synthase, elongation factors, and magnesium (Mg)–protoporphyrin chelatase. The results indicate that **A13** can change the physiological and biochemical characteristics of tobacco, thereby achieving disease resistance.

##### Kyoto Encyclopedia of Genes and Genomes (KEGG) Pathways

**Figure 8:**
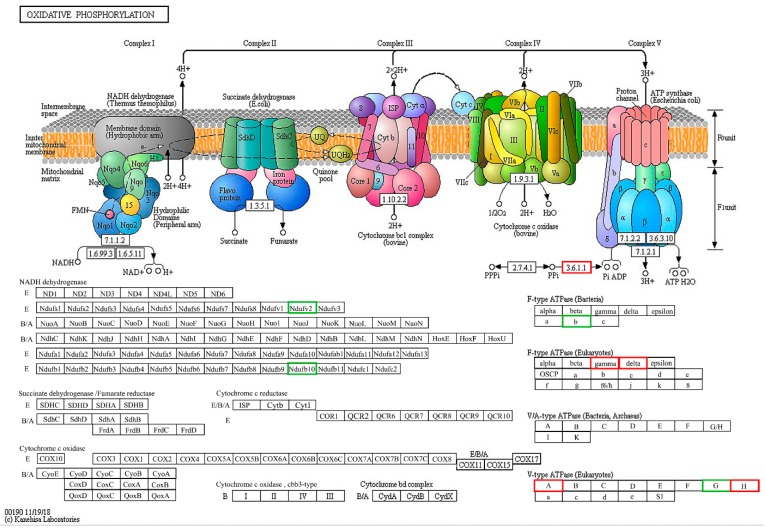
A KEGG map of the Oxidative phosphorylation pathway of DEPs in **A13** + TMV versus CK + TMV. The boxes with red frames correspond to the up-regulation of DEPs in the **A13** + TMV sample. The boxes with green frames correspond to the down-regulation of DEPs in the **A13** + TMV sample.

To study the potential link between DEPs and biological functions, we used the KEGG to identify potential pathways for different proteins in the treatment groups (**A13** + TMV versus CK + TMV). Different proteins were mapped using the KEGG database (*p* < 0.05). Oxidative phosphorylation (pathway ID, map00190) was found to be the main enrichment pathway, which includes 11 specific proteins, including the TMV capsid proteins. As shown in Table 2 and Figure 8, oxidative phosphorylation is an important pathway for different proteins. Five up-regulated and four down-regulated genes expressed proteins that regulate oxidative phosphorylation. The results indicate that compound **A13** enhances tobacco’s resistance to viral invasion by altering the up-regulation and down-regulation of differentially expressed proteins in the oxidative phosphorylation pathway.

## 3. Discussion

Resistance in plants is significantly associated with defensive enzymes, such as SOD, POD, CAT, and PAL [5]. The antioxidant defense mechanism of plants can protect plant cells from reactive oxygen species (ROS)-induced oxidative damage while influencing the photosynthetic state of plants [27]. CAT promotes the decomposition of H_2_O_2_ to prevent ROS-induced damage to plant cells [28]. POD induces the synthesis of SA, lignin, and antitoxin in plants, activates the salicylic acid (SA) pathway in plants, promotes cell wall thickening, inhibits the proliferation of pathogens, and causes plants to elicit an immune response [29]. The results of the defense enzyme activity test showed that **A13** activates its own defense system and increases disease resistance by increasing the defense enzyme activity of tobacco.

It is well-known that photosynthesis is a basic life process that green plants use to support their growth, development, and reproduction. Chlorophyll is required to maintain this vital feature [30]. In this study, we found the effect of compound **A13** on the chlorophyll content in tobacco is little compared with CK.

During a pathogen infection, the protein encoded by the defense gene participates in the immune response of the plant to the pathogen, thereby protecting the plant from the pathogen [31]. In plants, salicylic acid (SA) acts as an endogenous signaling molecule that induces hypersensitivity and a systemic acquired resistance to increase a plant’s disease resistance while also inducing some related resistance, and the expression of defense genes. A plant’s salicylic acid signaling pathway can regulate the production of salicylic acid, which is related to expression of the defense genes *ICS1*, *EDS1*, *SOD*, *CAT1*, *PAL4*, *PR1*, and *NPR1* [32,33]. These defense genes can induce the development of disease resistance [34,35,36]. SA can induce plant resistance, whereas *ICS1* and *EDS1* play an important role in the synthesis of SA [37,38]. The expression levels of *ICS1*, *CAT1*, and *PR1* in the **A13** + TMV treatment group indicate that compound **A13** can effectively enhance the activity of defense enzymes in plants and promote the accumulation of SA, which activates the systemic acquired resistance in a plants and is resistant to pathogens.

Oxidative phosphorylation is an important biochemical process in plants that provides energy for their life activities. This process is divided into substrate-level phosphorylation and oxidative phosphorylation. Oxidative phosphorylation is accompanied by ATP formation during biological oxidation and is regulated by key proteins, such as ATP synthase, cytochrome, and NADH dehydrogenase. The differential proteins inorganic pyrophosphatase (*Ppa*), soluble inorganic pyrophosphatase (*Ppa1*), ATP synthase gamma chain (*ATPeF1G*), ATP synthase delta chain (*ATPeF1D*), V-type proton ATPase catalytic subunit A (*ATPeV1A*), and V-type proton ATPase subunit H-like (*ATPeV1H*) were up-regulated (**A13** + TMV versus CK + TMV). Of which, *Ppa* catalyzes the decomposition of pyrophosphate into phosphoric acid, which is important for maintaining the reduction of cellular levels [39]. Furthermore, *Ppa1* controls the cellular level of inorganic pyrophosphate [40]. ATP synthase complexes of mitochondria and chloroplasts drive ADP to ATP conversion through transmembrane site gradients [41]. *ATPaF1G*, *ATPeF1D*, *ATPeV1A*, and *ATPeV1G* are ATP-related enzymes encoding genes that promote ADP to ATP conversion in plants to provide energy for a plant’s metabolism. It can be seen from the down-regulation of the ATP synthase subunit b protein that compound **A13** can accelerate the decomposition of ATP to provide energy, thereby preventing plants from resisting pathogen invasion. In summary, **A13** can enhance a plant’s immune response and resistance to TMV.

## 4. Materials and Methods

### 4.1. Inhibitory Activity Against TMV

#### 4.1.1. Purification of TMV

According to Gooding’s method [42], the upper leaves of *N. tabacum* L. that had been inoculated with TMV were ground with a silicate buffer and filtered with a double-layer de-esterase. The filtrate was centrifuged at 1000 rpm, treated twice with EG, and then centrifuged again. The entire experiment was conducted at 4 °C. The absorbance values were estimated at 260 nm by ultraviolet spectrophotometry.

Virus concentration (mg/mL) = (A_260_ × dilution ratio)/E^0.1%^1cm^260nm^, where E represents the extinction coefficient for TMV; and E^0.1%^1cm^260nm^ is 3.1

#### 4.1.2. The Curative Activities of the Compounds against TMV In Vivo

Leaves from the same *N. tabacum* L. plants were selected. TMV was inoculated on the leaves at a concentration of 6 × 10^−3^ mg/mL, and the leaves were uniformly covered with silicon carbide in advance. Then, using a brush, the compound solution was smeared on the left side of the blade and the right side was smeared with the solution that was used as a control. Tobacco leaves were inoculated with the virus for 3–4 days to record the number of lesions [19]. The measurements were tested in triplicate.

#### 4.1.3. The Protective Activities of the Compounds against TMV In Vivo

Leaves from the same *N. tabacum* L. plants were selected. Using a brush, we smeared the compound solution on the left side of the blade and the control on the right side. One day later, TMV was inoculated on the leaves at a concentration of 6 × 10^−3^ mg/mL, and the leaves were uniformly cove red with silicon carbide in advance. The tobacco leaves were inoculated with the virus for 3–4 days to record the number of lesions [19]. The measurements were tested in triplicate.

### 4.2. Mechanism of Inducing Resistance in Plants

#### 4.2.1. Determination of Defensive Enzyme Activities

Using a previously described method [43], the activities of SOD, POD, and CAT were measured and calculated with enzyme assay reagent kits (Suzhou Comin Bioengineering Institute, Suzhou, China), according to manufacturer’s instructions. The measurements were tested in triplicate.

#### 4.2.2. Chlorophyll Content

The chlorophyll content of each test sample was measured four times every day in accordance with a previously described method [19]. Each treated test sample was cut into small (50 mg) pieces using a hole puncher, each weighing 50 mg while avoiding the midrib and placed in a 5 mL mixed solution (V_85% ethanol_: V_85% acetone_ = 1:1). The sample was homogenized and incubated at 35 °C for 0.5 h and then centrifuged at 6500 rpm for 15 min. The absorption spectra of C_a_ and C_b_ at the wavelengths of 663 and 645 nm, respectively, were recorded in a reference solvent. The measurements were tested in triplicate. C_a_, C_b_, and the total chlorophyll content (Ct) were calculated using the following formula:

C_a_ (mg/L) = 9.784OD_663_ − 0.990OD_645_

C_b_ (mg/L) = 21.426OD_645_ − 4.650OD_663_

C_t_ (mg/L) = Ca + Cb = 5.134OD_663_ + 20.643OD_645_

Quantification (mg g^−1^ fresh weight) was calculated as follows:

Q = CV/1000W(1)

where C represents the concentration (mg/L); V represents the volume of solvent (mL); and W represents the sample’s fresh weight (g).

#### 4.2.3. Determination of the Relative Expression of Defense Genes 

Total RNA was extracted using a Trizol reagent kit (Sangon Biotech, Shanghai, China) according to the manufacturer’s instructions. RNA was reverse-transcribed using a cDNA kit (Sangon Biotech, Shanghai, China) in accordance with manufacturer’s instructions. The experiments were performed in 10 μL of reaction volume, which was analyzed using SYBR Premix Ex TaqII (Sangon) and an iCycleriQ multi-color real-time PCR Detection System (Bio-Rad, California, CA, USA). Table 3 shows the primer sequence information. Gene expression was normalized using β-actin as an internal control. The relative copy numbers of the genes were calculated in accordance with a previously described method [44]. The measurements were tested in triplicate.

#### 4.2.4. Differentially Expressed Protein Data Analysis

##### Tobacco Protein Extraction

All of the tobacco protein was extracted in accordance with a previously described method [45]. A tobacco leaf sample (1.0 g) was ground with nitrogen to homogenize the liquid, and the extract was suspended in 5 mL of low-temperature buffer (0.7 M sucrose, 0.5 M Tris-HCl, 0.1 M KCl, 50 mM diamine tetra acetic acid, 40 mM dithiothreitol, pH 7.5). After allowing the sample to stand for 10 min, an equal volume of the same buffer was added. The sample was agitated at 4 °C for 30 min. After 10 min of centrifugation, the supernatant was transferred to a new tube, in which 5 volumes of 100 mM ammonium acetate in methanol were added, and then stored at −20 °C for 12 h. The sample was centrifuged for 20 min (rotation speed: 5000 rpm, temperature: 4 °C), and the precipitate was collected and washed with 20% of 80% acetone. The precipitate was air-dried and dissolved in 150 μL of rehydration solution (8 M urea, 0.1 M Tris, 10 mM DTT, pH 8.5) for 1 h at 37 °C. The protein concentration was determined by using the Bradford method, and 100 μg of protein solution was collected. An equal volume of 55 mM iodoacetamide was added to the protein solution, which was incubated in a dark room for 30 min at room temperature. Then, it was centrifuged with 3 kDa Millipore water for 20 min (rotation speed: 5000 rpm, temperature: 4 °C). The protein solution was washed with diluent rehydration solution with the concomitant dissolution of 100 μL of Milli-Q water. The sample was centrifuged for 20 min (rotation speed: 5000 rpm, temperature: 4 °C). The collected polypeptide solution was air-dried and dissolved in 50 μL of HPLC-grade H_2_O, containing 0.1% formic acid (FA) for liquid chromatography–tandem mass spectrometry (LC-MS/MS). The measurements were tested in triplicate.

##### LC-MS/MS Analysis

We analyzed peptides by using a triple time-of-flight (TOF) 5600 mass spectrometer (Foster City, CA, USA). A peptide solution (8 μL) was injected in full-loop mode, and 1% acetonitrile (ACN) and 0.1% FA in water were equilibrated on a Chrom XP Trap column (Nano LC TRAP Column, 3 μm C18-CL, 120 Å, 350 μm × 0.5 mm, Foster City, CA, USA). The sample was washed for 10 minutes at a flow rate of 300 nL/min on reversed-phase chromatography (Nano LC C18, 3 μm C18-CL, 75 μm × 15 cm, Foster City, CA, USA) with a linear gradient formed by mobile phase A (5% ACN, 0.1% FA) and mobile phase B (95% ACN, 0.1% FA). The sample was washed again at a flow rate of 300 nL/min for 20 min. Peptides were eluted by the Triple TOF 5600 MS, which uses a data-dependent model with an automatic switch between TOF−MS and product ion acquisition by Analyst (R) Software (TF1.6) (Milwaukee City, WI, USA). β-Galactosidase was used to calibrate each pair of samples for 10 min of elution and 30 min of identification.

##### Proteomics Data Analysis

We used MaxQuant [46] version 1.5.2.8 by the Andromeda search engine, based on the tobacco proteome that was downloaded from UniProtto, analyze and quantify the LC-MS/MS data. The results of the MaxQuant analysis included an initial search with an initial mass tolerance of 20 ppm, respectively and were used for mass recalibration [47]. We searched for methionine oxidation, N-terminal acetylation, and cysteine methylation. The initial mass tolerance was presented in the main Andromeda search by precursor mass and fragment mass, with the values of 6 and 20 ppm. The peptide was seven amino acids to two miscleavages long, and the false discovery rate was set to 0.01. Using the minimum of the two ratio counts, we conducted label-free markerless quantitation to determine the normalized protein intensity [47]. The iBAQ algorithm was employed to rank the absolute abundances in individual samples [48]. Protein tables were filtered by eliminating identifications of common contaminants and reverse a database. An unpaired *t*-test of the iBAQ data of two-samples was used to identify DEPs between the treatment and control groups.

##### Bioinformatics and Annotations

The Uniprot software (http://www.uniprot.org/) was used to annotate the classification of DEPs by GO on the KEGG. GO items lacking the corresponding annotations were removed from the protein list, and the IDs listed for the proteins were mapped at the biological process, cellular component, and molecular function levels [49]. DEPs (expression levels > 2-fold) were mapped to the GO database (http://www.geneontology.org/), and the protein amount for each GO item was calculated. The label-free proteomics was used as the target list. The background list was generated by downloading the GO database.

#### 4.2.5. Statistical Analysis

To produce true and reliable data, we repeated each measurement three times thrice and completely arranged the results in a random design. SPSS version 16.0 (SPSS, Chicago, IL, USA) was used to analyze the experimental data. ANOVA and Tukey’s multiple comparison were used to determine significant difference (*p* < 0.05).

## 5. Conclusions

Taken together, this study shows that novel curcumin analogs bearing the quinazoline moiety have favorable inhibitory activity against TMV in vivo at 500 μg/mL. In particular, compound **A13** exhibited excellent protective activity, with the value of 70.4 ± 2.6%, which was better than that of **COS** (49.0 ± 5.9%). This protective activity of compound **A13** is due to its ability to induce disease resistance in plants by influencing defense-related enzymes, defense-related genes, and photosynthesis. In addition, compound **A13** can induce stress responses and oxidative phosphorylation. So, compound **A13** can induce plant disease resistance in plants and may be considered to be a new plant immune activator for controlling viruses in plants. Our data provide scientific evidence to support the further investigation of plant immune activators.

## Figures and Tables

**Table 1:** The antiviral activities of the target compounds against TMV in vivo.

Compound	R_1_	R_2_	Protection Activity ^a^ (%)	Curative Activity ^a^ (%)
**A-1**	7-Br	4-OCH_3_	39.7 ± 4.3	55.8 ± 3.7
**A-2**	7-Br	2-Cl	37.3 ± 5.0	18.8 ± 7.6
**A-3**	7-Br	4-F	50.6 ± 2.8	58.1 ± 10.0
**A-4**	7-Br	3,4-diOCH_3_	39.4 ± 1.9	20.2 ± 2.3
**A-5**	7-Br	4-CH_3_	27.4 ± 2.5	33.2 ± 1.9
**A-6**	7-Br	4-Br	46.5 ± 1.6	32.9 ± 7.1
**A-7**	7-Br	H	17.2 ± 6.2	9.3 ± 5.4
**A-8**	7-Br	4-Cl	47.3 ± 9.3	20.4 ± 1.7
**A-9**	7-Br	4-SCH_3_	48.8 ± 3.3	27.8 ± 8.0
**A-10**	6-Cl	4-SCH_3_	32.2 ± 7.2	34.0 ± 2.5
**A-11**	6-Cl	2,6-Cl	38 ± 1.3	49.9 ± 9.6
**A-12**	6-Cl	4-Cl	66.5 ± 3.0	42.0 ± 5.5
**A-13**	6-Cl	4-OCH_3_	70.4 ± 2.6	42.9 ± 2.2
**A-14**	6-Cl	2-Cl	37.9 ± 5.6	50.8 ± 7.7
**A-15**	6-Cl	3,4-diOCH_3_	47.6 ± 9.8	9.4 ± 1.5
**A-16**	6-Cl	4-Br	25.9 ± 4.2	38.1 ± 8.4
**A-17**	6-Cl	4-F	32.3 ± 9.1	37.4 ± 6.0
**A-18**	6-Cl	4-CH_3_	41.5 ± 8.7	13.9 ± 3.5
**A-19**	6-Cl	H	43.6 ± 4.7	20.0 ± 8.3
**A-20**	6-Cl	2-F	53.8 ± 3.0	21.9 ± 3.8
COS ^b^			49.0 ± 5.9	34.1 ± 3.8

^a^ The average of three replicates at 500 μg/mL. ^b^ The commercial anti-plant virus agent was used as the control. The ± values represent standard deviation.

**Table 2:** Differentially expressed proteins that are involved in the oxidative phosphorylation pathway.

ID	Protein Name	Gene Name	Regulated
Q43798	Inorganic pyrophosphatase (EC 3.6.1.1)	*Ppa*	Up
A0A1S4DBR1	soluble inorganic pyrophosphatase PPA1-like	*Ppa1*	Up
A0A1S4A0E0	ATP synthase gamma chain	*ATPeF1G*	Up
A0A1S4CSA5	ATP synthase delta chain	*ATPeF1D*	Up
A0A097BTV9	V-type proton ATPase catalytic subunit A	*ATPeV1A*	Up
A0A1S3Z6E6	V-type proton ATPase subunit H-like	*ATPeV1H*	Up
A0A1S4AX40	ATP synthase subunit b	*atpF*	Down
A0A1S4BUC0	NADH dehydrogenase [ubiquinone] 1 beta subcomplex subunit 10-A-lik	*NDUFV2*	Down
A0A1S4B7I9	V-type proton ATPase subunit	*ATPeV1G*	Down
A0A1S4BUC0	CP12 (Chloroplast protein 12)	*NDUFB10*	Down
Q7T699	Capsid protein, (Tobacco mosaic virus)	/	/

**Table 3:** The primer sequences and reaction condition used in RT-qPCR.

Gene Name	Forward Primer	Reverse Primer
*ICS1*	5′-CAGCGCTGGCCTTGGA-3′	5′-GGAGGTGGGTTGGATTTCAA-3′
*EDS1*	5′-GGCTCGAGTATGCCCTGAAG-3′	5′-CTTGCCCAGAAACATGATTCC-3′
*SOD*	5′-CGACACTAACTTTGGCTCCCTAGA-3′	5′-GGTTCCTCTTCTGGGAATAGACGT-3′
*CAT1*	5′-CAACTTCCTGCTAATGCTCCAA-3′	5′-TGCCTGTCTGGTGTGAATGA-3′
*PAL4*	5′-CTCGGCCCTCAGATCGAA-3′	5′-CCGAGTTGATCTCCCGTTCA-3′
*PR1*	5′-ATGGGATTTGTTCTCTTTTCACA-3′	5′-TTAGTATGGACTTTCGCCTCT-3′
*NPR1*	5′-GGCGAGGAGTCCGTTCTTTAA-3′	5′-TCAACCAGGAATGCCACAGC-3′

*ICS1*: Isochorismate synthase 1; *EDS1*: Enhanced Disease Susceptibility 1; *SOD*: Superoxide Dismutase; *CAT1*: catalase 1; *PAL4*: Phenylalanine Ammonia Lyase 4; *PR1*: Pathogenesis-Related genes 1; *NPR1*: Nonexpressor of Rathogenesis-Related genes

## Supplementary Materials

Supplementary materials can be found at http://www.mdpi.com/1422-0067/19/12/4065/s1.

## Abbreviations

TMV	Tobacco mosaic virus
COS	Chitosan oligosaccharide
DEPs	Differentially expressed protein
KEGG	Kyoto Encyclopedia of Genes and Genomes
