# Supplementary material for: Induced Resistance Mechanism of Novel Curcumin Analogs Bearing a Quinazoline Moiety to Plant Virus
Source: Int J Mol Sci. 2018 Dec 15;19(12):4065. doi: 10.3390/ijms19124065 (PMC6321402; doi:10.3390/ijms19124065)
Supplement: Supplementary file 1 [file ijms-19-04065-s001.zip › ijms-371229 supporting data.pdf]

# Supplementary Information

## Induced Resistance Mechanism of Novel Curcumin Analogs Bearing a Quinazoline Moiety in Plant Virus

### 1. Synthesis of the Target Compound

#### *1.1. Chemistry*

All solvents and reagents were purchased from commercial companies without further purification. All solvents are analytically pure, which were purified and dried before use according to standard methods. On GF254 analytical silica gel analyzed by thin layer chromatography (TLC), divided by column chromatography using silica gel (100–200 mesh), the melting point of the compound is measured by a WRX-4 microscopic melting point meter (Shanghai Yice Instrument Co., China). The samples were dissolved in $CDCl_3$ solution using tetramethylsilane as an internal standard, and NMR spectra were recorded on a Bruker Ascend™ 400M spectrometer. High-resolution mass spectrometer (HRMS) Data Collected on Thermo Scientific Q Exactive (Thermo, MO, USA).

#### *1.2. General Procedure for the Preparation of Target Compounds **A1** to **A20***

The synthetic route for target compounds **A1**–**A20** is illustrated in Scheme 1. Raw materials containing 2-aminobenzoic acid were heated at reflux with formamide for 7–8 h to obtain quinazolinone. Using DMF as catalyst, $SOCl_2$ and quinazolinone were heated at reflux for 8–9 h to get 4-chloroquinazoline. 4-Hydroxybenzaldehyde and acetone were stirred for 10–12 h to prepare (*E*)-4-(4-hydroxyphenyl)-but-3-en-2-one with 50% NaOH as the catalyst. With NaOH as the catalyst and ethanol as the solvent, intermediates (*E*)-4-(4-hydroxyphenyl)-but-3-en-2-one and aromatic aldehydes were stirred for 3–4 h under room temperature. To the system, 10% HCl was added to adjust the pH to 5–6, which precipitated a yellow solid. With $K_2CO_3$ as the acid-binding agent, intermediate 4-chloroquinazoline and **a-4** in $CH_3CN$ were stirred at 40–50 °C for 5–10 hours. The progress of the reaction was monitored by TLC. After completion of the reaction, a yellow turbid system was obtained, poured into 100 mL of water to precipitate a solid, which was filtered off with suction to give a solid. Then, it was recrystallized from ethyl acetate. The target compounds **A1**–**A20** were obtained with a yield of 30.0% to 75.0%.

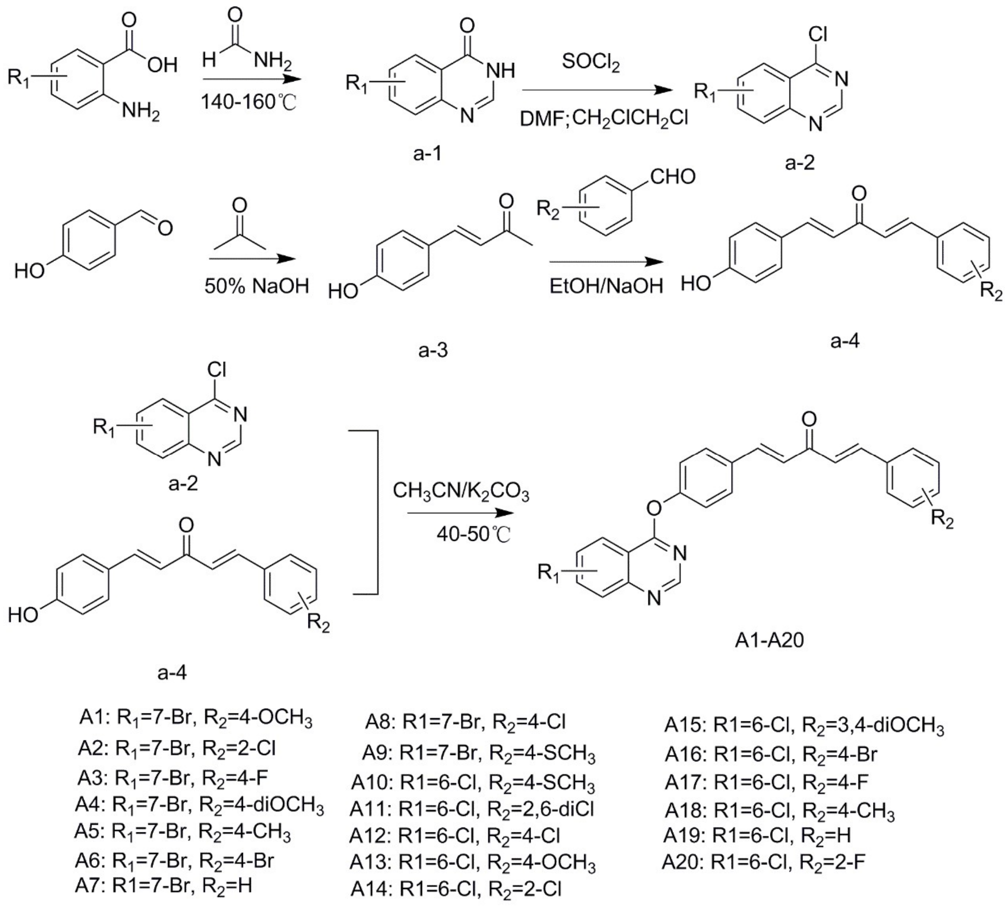


**Scheme 1.** Synthesis route of the target compounds.

## 2. The Characterization Data of the Target Compound

The characterization data of the target compound including $^{1}H$ NMR, $^{13}C$ NMR, $^{19}F$ NMR, and HRMS were shown as below.

Data for (1E,4E)-1-(4-methoxyphenyl)-5-(4-((7-bromoquinazolin-4-yl)oxy)phenyl) penta-1,4-dien-3-one (**A1**). Yellow solid; m.p. 183–185 °C; yield, 56.2%; $^{1}H$ NMR (400 MHz, $CDCl_3$) δ 8.77 (s, 1H, Qu-2-H), 8.23 (d, J = 10.7, 5.3 Hz, 2H, Qu-6,8-H), 7.76 (m, J = 16.4, 9.7, 4.9 Hz, 5H, Qu-5-H, $CH_3O$-Ph-3,5-H, C=O-C-CH), 7.59 (d, J = 8.7 Hz, 2H, O-Ph-3,5-H), 7.33 (d, J = 8.6 Hz, 2H, $CH_3O$-Ph-2,6-H), 7.08 (d, J = 15.9 Hz, 1H, C=O-CH), 6.96 (m, J = 12.3, 9.1 Hz, 3H, C=O-CH), 3.86 (s, 3H, -$OCH_3$); $^{13}C$ NMR (101 MHz, $CDCl_3$) δ: 188.70 (s), 166.81(s),161.80 (s), 155.21 (s), 153.62 (s), 152.63 (s), 143.45 (s), 141.58 (s), 133.15 (s), 131.48 (s), 130.65 (s), 130.26 (s), 129.85 (s), 129.21 (s), 127.51 (s), 125.99 (s), 124.99 (s), 123.40 (s), 122.49 (s), 115.08 (s), 114.53 (s), 55.48 (s); HRMS (ESI): Calculated for $C_{26}H_{20}BrN_2O_3$ $[M + H]^+$: 487.0651, found: 487.0643.

Data for (1E,4E)-1-(2-chlorophenyl)-5-(4-((7-bromoquinazolin-4-yl)oxy)phenyl) penta-1,4-dien-3-one (**A2**). Yellow solid; m.p. 143.6-145.7 °C; yield, 30.0%; $^{1}H$ NMR (400 MHz, $CDCl_3$) δ 8.78 (s, 1H, Qu-2-H), 8.24 (dd, *J* = 11.6, 5.3 Hz, 2H, Qu-6,8-H), 8.15 (d, *J* = 16.1 Hz, 1H, Qu-5-H), 7.80 (m, 1H, Cl-Ph-CH=), 7.76 (d, *J* = 8.8 Hz, 4H,Cl-6-H, O-Ph-CH=, O-Ph-3,5-H), 7.45 (m, 1H, Cl-4-H), 7.34 (m, *J* = 6.7, 2.0 Hz, 4H, O-Ph-2,6-H, Cl-Ar-3-H), 7.15 – 7.02 (m, 2H, Cl-Ph-5-H, Cl-Ph-C=CH); $^{13}C$ NMR (101 MHz, $CDCl_3$) δ: 188.68 (s), 166.76 (s), 155.17 (s), 153.77 (s), 152.60 (s), 142.49 (s), 139.21 (s), 135.44 (s), 133.06 (s), 132.88 (s), 131.47 (s), 131.27 (s), 130.63 (s),

130.33 (s), 129.95 (s), 129.20 (s), 128.16 (s), 127.72 (s), 127.17 (s), 125.17 (s), 124.96 (s), 122.53 (s), 115.03 (s); HRMS (ESI): Calculated for $C_{25}H_{17}BrClN_2O_2$ [M + H]$^+$: 491.0156, found: 491.0148.

Data for (1E,4E)-1-(4-fluorophenyl)-5-(4-((7-bromoquinazolin-4-yl)oxy)phenyl) penta-1,4-dien-3-one (**A3**). Yellow solid; m.p. 186.7-189.6 °C; yield, 35%; $^1$H NMR (400 MHz, $CDCl_3$) δ 8.78 (s, 1H, Qu-2-H), 8.23 (dd, *J* = 9.8, 5.3 Hz, 2H, Qu-6,8-H), 7.80 (m, 2H, O-Ph-C=H, F-Ph-C=H), 7.75 (m, 2H, F-Ph-3,5-H), 7.70 (s, 1H, Qu-5-H) ), 7.62 (m, *J* = 5.1, 3.5 Hz, 2H, O-Ph-3,5-H), 7.34 (d, *J* = 8.7 Hz, 2H, F-Ph-2,6-H), 7.11 (d, *J* = 8.6 Hz, 2H, 2CH-C=O), 7.06 – 6.99 (m, 2H,O-Ph-2,6-H); $^{13}$C NMR (101 MHz, $CDCl_3$) δ: 188.49 (s), 166.75 (s), 155.16 (s), 153.74 (s), 152.61 (s), 142.18 (d, *J* = 9.4 Hz), 132.91 (s), 131.47 (s), 131.03 (s), 130.63 (s), 130.33 (d, *J* = 8.5 Hz), 129.89 (s), 129.20 (s), 125.77 (s), 125.04 (d, *J* = 19.8 Hz), 122.52 (s), 116.31 (s), 116.09 (s), 115.02 (s); $^{19}$F NMR (376 MHz, $CDCl_3$) δ -108.92 (s); HRMS (ESI): Calculated for $C_{25}H_{17}BrFN_2O_2$ [M + H]$^+$: 475.0452, found: 475.0429.

Data for (1E,4E)-1-(3,4-dimethoxyphenyl)-5-(4-((7-bromoquinazolin-4-yl)oxy)phenyl) penta-1,4-dien-3-one (**A4**). Yellow solid; m.p. 166.0-168.0 °C; yield, 35.6%; $^1$H NMR (400 MHz, $CDCl_3$) δ 8.78 (s, 1H, Qu-2-H ), 8.23 (dd, *J* = 12.1, 5.2 Hz, 2H, Qu-6,8-H ), 7.80 – 7.70 (m, 5H, Qu-5-H, O-Ph-CH=, $CH_3$O-Ph-C<u>H</u>=, O-Ph-3,5-H), 7.33 (d, *J* = 8.6 Hz, 2H, 2H, $CH_3$O-Ph-2,5-<u>H</u> ), 7.22 (dd, *J* = 8.0, 1.5 Hz, 1H, $CH_3$O-Ph-6-<u>H</u>), 7.14 (dd, *J* = 17.2, 8.6 Hz, 2H, C<u>H</u>=CO=C<u>H</u>), 6.93 (dd, *J* = 17.0, 12.1 Hz, 2H, O-Ph-2,6-H), 3.95 (d, *J* = 6.4 Hz, 6H, 2-$OCH_3$); $^{13}$C NMR (101 MHz, $CDCl_3$) δ: 188.68 (s), 166.86 (s), 155.25 (s), 153.66 (s), 152.65 (s), 151.58 (s), 149.36 (s), 143.79 (s), 141.74 (s), 133.16 (s), 131.55 (s), 130.69 (s), 129.94 (s), 129.28 (s), 127.75 (s), 125.74 (s), 125.05 (s), 123.81 (s), 123.41 (s), 122.56 (s), 115.10 (s), 111.19 (s), 109.90 (s), 56.13 (s), 56.04 (s); HRMS (ESI): Calculated for $C_{27}H_{22}BrN_2O_4$ [M + H]$^+$: 517.0757, found: 517.0750.

Data for (1E,4E)-1-(4-methylphenyl)-5-(4-((7-bromoquinazolin-4-yl)oxy)phenyl) penta-1,4-dien-3-one (**A5**). Yellow solid; m.p. 184.0-186.0 °C; yield, 52.0%;$^1$H NMR (400 MHz, $CDCl_3$) δ 8.78 (s, 1H, Qu-2-H ), 8.23 (dd, *J* = 12.1, 5.2 Hz, 2H, Qu-6,8-H ), 7.80 – 7.70 (m, 5H, Qu-5-H, O-Ph-CH=, $CH_3$O-Ph-C<u>H</u>=, O-Ph-3,5-H), 7.33 (d, *J* = 8.6 Hz, 2H, 2H, $CH_3$O-Ph-2,5-<u>H</u> ), 7.22 (dd, *J* = 8.0, 1.5 Hz, 1H, $CH_3$O-Ph-6-<u>H</u>), 7.14 (dd, *J* = 17.2, 8.6 Hz, 2H, C<u>H</u>=CO=C<u>H</u>), 6.93 (dd, *J* = 17.0, 12.1 Hz, 2H, O-Ph-2,6-H), 3.95 (d, *J* = 6.4 Hz, 6H, 2-$OCH_3$); $^{13}$C NMR (101 MHz, $CDCl_3$) δ: 188.68 (s), 166.86 (s), 155.25 (s), 153.66 (s), 152.65 (s), 151.58 (s), 149.36 (s), 143.79 (s), 141.74 (s), 133.16 (s), 131.55 (s), 130.69 (s), 129.94 (s), 129.28 (s), 127.75 (s), 125.74 (s), 125.05 (s), 123.81 (s), 123.41 (s), 122.56 (s), 115.10 (s), 111.19 (s), 109.90 (s), 56.13 (s), 56.04 (s); HRMS (ESI): Calculated for $C_{26}H_{20}BrN_2O_2$ [M + H]$^+$: 471.0630, found: 471.06931.

Data for (1E,4E)-1-(4-bromophenyl)-5-(4-((7-bromoquinazolin-4-yl)oxy)phenyl) penta-1,4-dien-3-one (**A6**). White solid ; m.p. 184.0-186.0 °C; yield, 72.76%;$^1$H NMR (400 MHz, $CDCl_3$) δ: 8.78 (s, 1H, Qu-2-H), 8.25 (d, *J* = 8.8 Hz, 1H, Qu-8-H), 8.22 (d, *J* = 1.8 Hz, 1H, Qu-6-H), 7.79 (dd, *J* = 8.7, 2.0 Hz, 2H, CH=C-C=O-C=CH), 7.75 (d, *J* = 8.4 Hz, 2H, Br-Ph-2,6-H), 7.67 (s, 1H, Qu-5-H), 7.57 – 7.55 (m, 2H, Br-Ph-3,5-H), 7.51 – 7.48 (m, 2H, O-Ph-3,5-H), 7.34 (d, *J* = 8.7 Hz, 2H, CH=C=O=CH), 7.10 – 7.04 (m, 2H, O-Ph-2,6-H); $^{13}$C NMR (101 MHz, $CDCl_3$) δ: 188.52 (s), 166.83 (s), 155.23 (s), 153.84 (s), 152.67 (s), 142.42 (s), 142.20 (s), 133.74 (s), 132.92 (s), 132.35 (s), 131.56 (s), 130.70 (s), 130.02 (s), 129.86 (s), 129.29 (s), 125.90 (s), 125.78 (s), 125.03 (s), 124.97 (s), 122.62 (s), 115.09 (s); HRMS (ESI): Calculated for $C_{25}H_{17}Br_2N_2O_2$ [M + H]$^+$: 534.9651, found: 534.9624.

Data for (1E,4E)-1-(4-methylphenyl)-5-(4-((7-bromoquinazolin-4-yl)oxy)phenyl) penta-1,4-dien-3-one (**A7**). Yellow solid; m.p.205.9-206.8 °C; yield, 65.0%; $^1$H NMR (400 MHz, $CDCl_3$) δ 8.78 (s, 1H, Qu-2-H), 8.28 – 8.19 (m, 2H, Qu-6,8-H), 7.77 (dd, *J* = 14.8, 7.7 Hz, 5H, Qu-5-H, O-Ph-C<u>H</u>=, Ph-C<u>H</u>=, Ph-3,5-<u>H</u>), 7.63 (s, 2H, O-Ph-3,5-H ), 7.43 (s, 3H, Ph-3,4,5-H), 7.34 (d, *J* = 7.9 Hz, 2H, C<u>H</u>=CO=C<u>H</u>), 7.10 (d, *J* = 15.9 Hz, 2H, O-Ph-2,6-H); $^{13}$C NMR (101 MHz, $CDCl_3$) δ: 188.76 (s), 166.78 (s), 155.18 (s), 153.68 (s), 152.57 (s), 143.60 (s), 142.08 (s), 134.73 (s), 132.96 (s), 131.49 (s), 130.63 (s),130.66 (s), 129.93 (s), 129.22 (s), 129.03 (s), 128.47 (s), 125.74 (s), 125.44 (s), 124.98 (s), 122.52 (s), 115.02 (s); HRMS (ESI): Calculated for $C_{25}H_{18}BrN_2O_2$ [M + H]$^+$: 457.0546, found: 457.0527.

Data for (1E,4E)-1-(4-chlorophenyl)-5-(4-((7-bromoquinazolin-4-yl)oxy)phenyl) penta-1,4-dien-3-one (**A8**). Yellow solid; m.p. 175.5-176.4 °C; yield, 50.0%; $^1$H NMR (400 MHz, $CDCl_3$) δ: 8.77 (s, 1H, Qu-2-H), 8.23 (dd, *J* = 9.4, 5.3 Hz, 2H, Qu-6,8-H), 7.81 – 7.77 (m, 2H, O-Ph-C<u>H</u>=, Ph-C<u>H</u>=,), 7.75 (d, *J* = 8.5 Hz, 2H, Cl-Ph-3,5-H), 7.70 (d, *J* = 15.9 Hz, 1H, Qu-5-H), 7.56 (d, *J* = 8.5 Hz, 2H, Cl-Ph-2,6-H), 7.40 (d, *J* = 8.5 Hz, 2H, O-Ph-3,5-H), 7.34 (d, *J* = 8.7 Hz, 2H, C<u>H</u>=CO=C<u>H</u> ), 7.06 (dd, *J* = 15.9, 3.4 Hz, 2H, O-Ph-2,6-H); $^{13}$C NMR (101 MHz, $CDCl_3$) δ: 188.43 (s), 166.74 (s), 155.15 (s), 153.78 (s), 152.61 (s), 142.29 (s), 142.04 (s), 136.52 (s), 133.27 (s), 132.86 (s), 131.48 (s), 130.63 (s), 129.91 (s), 129.57 (s), 129.30 (s), 129.11 (s), 125.77 (s), 125.73 (s), 124.94 (s), 122.53 (s), 115.02 (s); HRMS (ESI): Calculated for $C_{25}H_{17}BrClN_2O_2$ [M + H]$^+$: 491.0156, found: 491.0147.

Data for (1E,4E)-1-(4-(methylthio)phenyl)-5-(4-((7-bromoquinazolin-4-yl)oxy)phenyl) penta-1,4-dien-3-one (**A9**). Yellow solid; m.p. 155.3-156.2 °C; yield, 52.0%;$^1$H NMR (400 MHz, $CDCl_3$) δ: 8.78 (s, 1H, Qu-2-H), 8.23 (dd, *J* = 10.3, 5.3 Hz, 2H, Qu-6,8-H), 7.81 – 7.76 (m, 2H, O-Ph-C<u>H</u>=, Ph-C<u>H</u>=), 7.76 – 7.70 (m, 3H, Qu-5-H, O-Ph-3,5-H), 7.54 (d, *J* = 8.4 Hz, 2H, S-Ph-2,6-H), 7.33 (d, *J* = 8.6 Hz, 2H, S-Ph-3,5-H), 7.28 (s, 1H,O-Ph-C=CH), 7.25 (s, 1H,S-Ph-C=CH), 7.06 (t, *J* = 16.2 Hz, 2H,O-Ph-2,6-H), 2.53 (s, 3H,-$SCH_3$); $^{13}$C NMR (101 MHz, $CDCl_3$) δ: 188.60 (s), 166.77 (s), 155.17 (s), 153.66 (s), 152.60 (s), 143.04 (s), 142.52 (s), 141.85 (s), 133.02 (s), 131.46 (s), 131.23 (s), 130.63 (s), 129.86 (s), 128.80 (s), 126.0 (s), 125.85 (s), 124.95 (s), 124.49 (s), 122.48 (s), 15.15 (s); HRMS (ESI): Calculated for $C_{26}H_{20}BrSN_2O_2$ [M + H]$^+$: 503.0423, found: 503.0416.

Data for (1E,4E)-1-(4-(methylthio)phenyl)-5-(4-((6-chloroquinazolin-4-yl)oxy)phenyl)penta-1,4-dien-3-one (**A10**). Yellow solid; m.p. 187.6-190.5 °C; yield, 65.0%;$^1$H NMR (400 MHz, $CDCl_3$) δ: 8.78 (s, 1H Qu-2-H), 8.37 (d, *J* = 2.3 Hz, 1H, Qu-8-H), 7.98 (d, *J* = 9.0 Hz, 1H, Qu-7-H), 7.87 (dd, *J* = 9.0, 2.3 Hz, 1H, Qu-5-H), 7.74 (dd, *J* = 15.3, 10.6 Hz, 4H, O-Ph-3,5-H, 2C=O-C=CH), 7.54 (d, *J* = 8.4 Hz, 2H, S-2,6-H,), 7.34 (d, *J* = 8.6 Hz, 2H,S-3,5-H), 7.26 (d, *J* = 4.0 Hz, 2H, 2C=O-CH), 7.06 (dd, *J* = 17.2, 16.0 Hz, 2H,O-Ph-2,6-H), 2.53 (s, 3H,$SCH_3$); $^{13}$C NMR (101 MHz, $CDCl_3$) δ: 188.38 (s), 165.69 (s), 154.06 (s), 153.41 (s), 150.07 (s), 142.82 (s), 142.29 (s), 141.63 (s), 135.02 (s), 133.32 (s), 132.81 (s), 131.00 (s), 129.60 (s),126.66 (s), 128.58 (s), 125.70 (d, *J* = 15.2 Hz), 124.26 (s), 122.47 (s), 122.24 (s), 116.80 (s), 14.92 (s); HRMS (ESI): Calculated for $C_{26}H_{20}ClSN_2O_2$ [M + H]$^+$: 459.0928, found: 459.0913.

Data for (1E,4E)-1-(2,4-dichlorophenyl)-5-(4-((6-chloroquinazolin-4-yl)oxy)phenyl) penta-1,4-dien-3-one (**A11**). Yellow solid; m.p. 183.0-185.0 °C; yield, 50.0%; $^1$H NMR (400 MHz, $CDCl_3$) δ 8.78 (s, 1H, Qu-2-H), 8.23 (dd, *J* = 10.6, 5.3 Hz, 2H, Qu-7,8-H), 7.82 – 7.73 (m, 5H, Qu-

5-H, Cl-Ph-3,5-H, O-Ph-3,5-H), 7.69 (d, *J* = 15.9 Hz, 1H, Cl-Ph-4-H), 7.56 (d, *J* = 1.8 Hz, 1H, Cl-Ph-CH=), 7.49 (d, *J* = 8.1 Hz, 1H, O-Ph-CH= ), 7.34 (d, *J* = 8.6 Hz, 2H, CH-C=O-CH), 7.10 – 7.04 (m, 2H, O-Ph-2,6-H); $^{13}$C NMR (101 MHz, $CDCl_3$) δ 188.45 (s), 166.76 (s), 155.16 (s), 153.77 (s), 152.58 (s), 142.35 (s), 142.13 (s), 133.67 (s), 132.85 (s), 132.28 (s), 131.50 (s), 130.62 (s), 129.95 (s), 129.79 (s), 129.23 (s) (s), 125.83 (s), 125.70 (s), 124.96 (s), 124.90 (s), 122.55 (s), 115.01 (s); HRMS (ESI): Calculated for $C_{25}H_{16}Cl_3N_2O_2$ [M + H]$^+$: 481.0271, found: 481.0253.

Data for (1E,4E)-1-(4-chlorophenyl)-5-(4-((6-chloroquinazolin-4-yl)oxy)phenyl) penta-1,4-dien-3-one (**A12**). Yellow solid; m.p. 169.1-170.0°C; yield, 60.0%;$^1$H NMR (400 MHz, $CDCl_3$) δ: 8.78 (s, 1H, Qu-2-H,), 8.37 (d, *J* = 2.2 Hz, 1H, Qu-8-H,), 7.99 (d, *J* = 9.0 Hz, 1H, Qu72-H,), 7.88 (dd, *J* = 9.0, 2.3 Hz, 1H, Qu-5-H,), 7.80 – 7.69 (m, 4H, 2C=O-C=CH, Cl-Ph-3,5-H), 7.57 (d, *J* = 8.5 Hz, 2H, Cl-Ph-2,6-H), 7.40 (d, *J* = 8.5 Hz, 2H, O-Ph-3,5-H), 7.34 (d, *J* = 8.6 Hz, 2H,2C=O-CH), 7.07 (dd, *J* = 15.9, 3.7 Hz, 2H, O-Ph-2,6-H); $^{13}$C NMR (101 MHz, $CDCl_3$) δ: 188.52 (s), 165.97 (s), 154.34 (s), 153.83 (s), 150.38 (s), 142.39 (s), 142.13 (s), 136.59 (s), 135.35 (s), 133.64 (s), 133.33 (s), 132.95 (s),130.02, 129.86.129.66 (s), 129.39 (s), 125.84(s), 125.80(s), 122.77(s), 122.59 (s), 117.09 (s); HRMS (ESI): Calculated for $C_{25}H_{17}Cl_2N_2O_2$ [M + H]$^+$: 447.0661, found: 447.0645.

Data for (1E,4E)-1-(4-methoxyphenyl)-5-(4-((6-chloroquinazolin-4-yl)oxy)phenyl)penta-1,4-dien -3-one (**A13**). Yellow solid; m.p. 179–181 °C; yield, 75.0%; $^1$H NMR (400 MHz, $CDCl_3$) δ : 8.78 (s, 1H, Qu-2-H), 8.37 (s, 1H, Qu-5-H), 7.98 (d, J = 8.9 Hz, 1H, Qu-8-H ), 7.87 (d, J = 8.2 Hz, 1H, Qu-7-H), 7.75 (dd, J = 16.7, 10.5 Hz, 4H, 2C=O-C=CH, $CH_3$O-Ph-3,5-<u>H</u>), 7.59 (d, J = 8.3 Hz, 2H, O-Ph-3,5-H), 7.33 (d, J = 8.1 Hz, 2H, $CH_3$O-Ph-2,6-<u>H</u>), 7.09 (d, J = 15.9 Hz, 1H, O-Ph-C=CH), 6.96 (t, J = 11.3 Hz, 3H, $CH_3$O-Ph-C=CH, O-Ph-2,6-H), 3.86 (s, 3H,-$CH_3$); $^{13}$C NMR (101 MHz, $CDCl_3$) δ 188.66 (s), 165.90 (s), 161.71 (s), 154.27 (s), 153.51 (s), 150.24 (s), 143.40 (s), 141.54 (s), 135.22 (s), 133.49 (s), 133.09 (s), 130.22 (s),129.84 (s) ,129.73 (s), 127.40 (s), 125.90 (s), 123.31 (s), 122.68 (s), 122.42 (s), 116.99 (s), 114.45 (s), 55.43 (s); HRMS (ESI): Calculated for $C_{26}H_{19}ClN_2O_3$ [M + H]+: 443.1157, found: 443.1154.

Data for (1E,4E)-1-(2-chlorophenyl)-5-(4-((6-chloroquinazolin-4-yl)oxy)phenyl)penta-1,4- dien-3-one (**A14**). Yellow solid; m.p. 154.3-156.0 °C; yield, 70.0%;$^1$H NMR (400 MHz, $CDCl_3$) δ 8.78 (s, 1H, Qu-2-H), 8.37 (d, *J* = 2.2 Hz, 1H, Qu-8-H), 8.15 (d, *J* = 16.1 Hz, 1H, Qu-7-H), 7.98 (d, *J* = 9.0 Hz, 1H, Qu-5-H), 7.88 (dd, *J* = 9.0, 2.3 Hz, 1H, Cl-Ph-CH=), 7.81 – 7.70 (m, 5H, O-Ph-CH=, O-Ph-3,5-H, Cl-Ph-3,6-H ), 7.46 (dd, *J* = 7.4, 1.8 Hz, 1H, Cl-Ph-5-H), 7.34 (s, 3H, Cl-Ph-C=CH, Cl-Ph-4-H, O-Ph-C=CH), 7.16 – 7.03 (m, 2H, O-Ph-2,6-H); $^{13}$C NMR (101 MHz, $CDCl_3$) δ 188.67 (s), 165.90 (s), 154.27 (s), 153.75 (s), 150.30 (s), 142.49 (s), 139.20 (s), 135.26, 135.44 , 133.55 (s), 132.77, 13129.62 13, 131.26 (s), 130.32 (s), 129.96 (s),129.77 (s), 128.16 (s), 127.72 (s), 127.16 (s), 125.18 (s), 122.69 (s),126.50(s), 117.02 (s); HRMS (ESI): Calculated for $C_{25}H_{22}Cl_2N_2O_4$ [M + H]$^+$: 473.1262, found: 473.1254.

Data for (1E,4E)-1-( 3,4-dimethoxyphenyl)-5-(4-((6-chloroquinazolin-4-yl)oxy)phenyl)penta-1,4- dien-3-one (**A15**). Yellow solid; m.p. 209.3-210.0 °C; yield, 60.0%;$^1$H NMR (400 MHz, $CDCl_3$) δ: 8.78 (s, 1H, Qu-2-H), 8.37 (d, *J* = 2.3 Hz, 1H, Qu-8-H), 7.98 (d, *J* = 9.0 Hz, 1H, Qu-7-H), 7.88 (dd, *J* = 9.0, 2.4 Hz, 1H, Qu-5-H), 7.78 (t, *J* = 6.6 Hz, 2H, C<u>H</u>=C-C=O-C=C<u>H</u>), 7.74 (d, *J* = 2.4 Hz, 2H, O-Ph-3,5-H), 7.34 (d, *J* = 8.6 Hz, 2H, $CH_3$O-Ph-3,6-H), 7.23 (dd, *J* = 8.3, 1.9 Hz, 1H, $CH_3$O-Ph-5-<u>H</u>), 7.16 – 7.09 (m, 2H, =CH-CO-CH), 6.93 (d, *J* = 4.2 Hz, 2H, O-Ph-2,6-H), 3.95 (d, *J* = 6.5 Hz, 6H, 2-$OCH_3$); $^{13}$C NMR (101 MHz, $CDCl_3$) δ: 188.68 (s), 166.00 (s), 154.36 (s), 153.66 (s), 151.61 (s), 150.37 (s), 149.40 (s), 143.76 (s), 141.72 (s), 135.33 (s), 133.62 (s), 133.19 (s), 129.85 (s), 125.93

(s), 127.79 (s), 125.77 (s), 123.84 (s), 123.37 (s), 122.77 (s), 122.53 (s), 117.11 (s), 111.24 (s), 110.00 (s), 56.09 (s), 55.05 (s); HRMS (ESI): Calculated for $C_{27}H_{22}ClN_2O_4$ $[M + H]^+$: 473.1262, found: 473.1254.

Data for (1E,4E)-1-(4-bromophenyl)-5-(4-((6-chloroquinazolin-4-yl)oxy)phenyl)penta-1,4-dien-3-one (**A16**). Yellow solid; m.p. 186.7-188.2 °C; yield, 45.0%; $^1$H NMR (400 MHz, $CDCl_3$) δ: 8.78 (s, 1H, Qu-2-H), 8.37 (d, *J* = 2.2 Hz, 1H, Qu-8-H), 7.98 (d, *J* = 9.0 Hz, 1H, Qu-7-H), 7.88 (dd, *J* = 9.0, 2.3 Hz, 1H, Qu-5-H), 7.72 (dd, *J* = 26.5, 12.1 Hz, 4H, CH=C-C=O-C=CH, Br-Ph-2,6-H), 7.56 (d, *J* = 8.5 Hz, 2H, Br-Ph-3,5-H), 7.49 (d, *J* = 8.5 Hz, 2H, O-Ph-3,5-H), 7.34 (d, *J* = 8.6 Hz, 2H, CH-C=O-CH), 7.08 (dd, *J* = 15.9, 3.1 Hz, 2H, O-Ph-2,6-H); $^{13}$C NMR (101 MHz, $CDCl_3$) δ: 188.45(s), 165.90 (s), 154.26 (s), 153.74 (s), 150.28 (s), 142.36 (s), 135.29 (s), 133.66 (s), 133.56 (s), 132.86 (s), 132.27 (s), 129.97 (s), 129.80 (s), 129.78 (s), 125.83 (s), 125.70(s), 124.90 (s), 122.70 (s), 122.52 (s), 117.01 (s); HRMS (ESI): Calculated for $C_{25}H_{17}BrClN_2O_4$ $[M + H]^+$: 491.0156, found: 491.0141.

Data for (1E,4E)-1-(4-floophenyl)-5-(4-((6-chloroquinazolin-4-yl)oxy)phenyl)penta-1,4- dien-3-one (**A17**). Yellow solid; m.p.166.6-169.8 °C; yield, 54.0%; $^1$H NMR (400 MHz, $CDCl_3$) δ 8.78 (s, 1H, Qu-2-H), 8.37 (d, *J* = 2.1 Hz, 1H Qu-8-H), 7.98 (d, *J* = 9.0 Hz, 1H, Qu-7-H), 7.88 (dd, *J* = 9.0, 2.2 Hz, 1H, Qu-5-H), 7.80 – 7.72 (m, 4H, CH=C-C=O-C=CH, F-Ph-2,6-H), 7.64 – 7.60 (m, 2H, F-Ph-3,5-H), 7.34 (d, *J* = 8.5 Hz, 2H, O-Ph-3,5-H ), 7.13 (d, *J* = 8.5 Hz, 2H, CH-C=O-CH ), 7.08 – 6.99 (m, 2H, O-Ph-2,6-H ); $^{13}$C NMR (101 MHz, $CDCl_3$) δ 188.51 (s), 165.91 (s), 165.36 (s), 162.85 (s), 154.27 (s), 153.69 (s), 150.28 (s), 142.21 (d, *J* = 9.3 Hz), 135.28 (s), 133.55 (s), 132.91 (s), 130.99 (d, *J* = 3.3 Hz), 130.36 (d, *J* = 8.6 Hz), 129.94 (s), 129.77 (s), 125.75 (s), 125.10 (d, *J* = 2.2 Hz), 122.70 (s), 122.51 (s), 117.01 (s), 116.32 (s), 116.10 (s); $^{19}$F NMR (376 MHz, $CDCl_3$) δ -108.91 (s); HRMS (ESI): Calculated for $C_{25}H_{17}FClN_2O_4$ $[M + H]^+$: 431.0957, found: 431.0951.

Data for (1E,4E)-1-(4-methylphenyl)-5-(4-((6-chloroquinazolin-4-yl)oxy)phenyl)penta-1,4-dien-3-one (**A18**). Yellow solid; m.p. 191.0-192.3 °C; yield, 60.0%; $^1$H NMR (400 MHz, $CDCl_3$) δ: 8.78 (s, 1H, Qu-2-H), 8.37 (d, *J* = 2.0 Hz, 1H, Qu-8-H), 7.98 (d, *J* = 9.0 Hz, 1H, Qu-7-H), 7.88 (dd, *J* = 9.0, 2.4 Hz, 1H, Qu-5-H), 7.75 (dd, *J* = 9.8, 6.4 Hz, 4H, CH=C-C=O-C=CH, $CH_3$-Ph-3,5-H), 7.54 (d, *J* = 8.1 Hz, 2H, O-Ph-3,5-H), 7.34 (d, *J* = 8.6 Hz, 2H, $CH_3$-Ph-2,6-H), 7.24 (d, *J* = 8.0 Hz, 2H, CH-C=O-CH), 7.12 – 7.02 (m, 2H, O-Ph-2,6-H), 2.40 (s, 3H, $-CH_3$); $^{13}$C NMR (101 MHz, $CDCl_3$) δ: 188.60 (s), 165.71 (s), 154.08 (s), 153.38 (s), 150.06 (s), 143.46 (s), 141.60 (s), 140.99 (s), 135.04 (s), 133.32 (s), 132.84 (s), 131.78 (s), 129.68 (s), 129.56 (s), 128.28 (s), 125.60 (s), 124.35 (s), 122.49 (s), 122.25 (s), 116.81 (s), 21.37 (s); HRMS (ESI): Calculated for $C_{25}H_{17}FClN_2O_4$ $[M + H]^+$: 427.1135, found: 427.1194.

Data for (1E,4E)-1-phenyl-5-(4-((6-chloroquinazolin-4-yl)oxy)phenyl)penta-1,4- dien-3-one (**A19**). Yellow solid; m.p. 179–181 °C; yield, 75.0%; $^1$H NMR (400 MHz, $CDCl_3$) δ: 8.78 (s, 1H, Qu-2-H), 8.37 (d, *J* = 2.3 Hz, 1H, Qu-8-H), 7.98 (d, *J* = 9.0 Hz, 1H, Qu-7-H), 7.87 (dd, *J* = 9.0, 2.3 Hz, 1H, Qu-5-H ), 7.81 – 7.73 (m, 4H, CH=C-C=O-C=CH, Ph-2,6-H), 7.64 (dd, *J* = 6.6, 2.9 Hz, 2H, O-Ph-3,5-H), 7.42 (dd, *J* = 6.2, 3.8 Hz, 3H, Ph-3,4,5-H), 7.35 (t, *J* = 5.6 Hz, 2H, CH-C=O-CH), 7.10 (dd, *J* = 15.9, 1.4 Hz, 2H, O-Ph-2,6-H); $^{13}$C NMR (101 MHz, $CDCl_3$) δ: 188.82 (s), 165.98 (s), 154.35 (s), 153.73 (s), 150.35 (s), 143.65 (s), 142.14 (s), 135.33 (s), 134.81 (s), 133.62 (s), 133.05 (s), 130.70 (s), 130.00, 129.84, 129.10 (s), 128.54 (s), 125.82 (s), 125.52 (s), 122.78 (s), 122.56 (s), 117.09 (s); HRMS (ESI): Calculated for $C_{25}H_{18}ClN_2O_2$ $[M + H]^+$: 412.0979, found: 431.0927.

Data for (1E,4E)-1-(2-fluorophenyl-5-(4-((6-chloroquinzolin-4-yl)oxy)phenyl)penta-1,4- dien-3-one (**A20**). Yellow solid; m.p. 160.9-161.9 °C; yield, 50.0%; $^{1}H$ NMR (400 MHz, $CDCl_3$,) δ: 8.78 (s, 1H, Qu-2-H), 8.37 (d, *J* = 2.2 Hz, 1H, Qu-8-H), 7.98 (d, *J* = 9.0 Hz, 1H, Qu-7-H), 7.90 – 7.85 (m, 2H, Ph-CH=, Qu-5-H), 7.78 (dd, *J* = 12.1, 10.2 Hz, 3H, O-Ph-CH=, O-Ph-3,5-H), 7.67 – 7.62 (m, 1H, F-Ph-3-H), 7.42 – 7.33 (m, 3H, F-Ph-4,5,6-H), 7.20 (dd, *J* = 15.5, 7.7 Hz, 2H, CH-C=O-CH), 7.15 – 7.07 (m, 2H, O-Ph-2,6-H); $^{13}C$ NMR (101 MHz, $CDCl_3$) δ 188.72 (s), 165.84 (s), 162.87 (s), 160.35 (s), 154.22 (s), 153.65 (s), 150.22 (s), 142.28 (s), 136.03 (d, *J* = 2.5 Hz), 135.19 (s), 133.47 (s), 132.85 (s), 131.89 (d, *J* = 8.8 Hz), 129.91 (s), 129.70 (s), 129.40 (d, *J* = 2.8 Hz), 127.75 (d, *J* = 6.6 Hz), 125.53 (s), 124.52 (d, *J* = 3.6 Hz), 122.64 (s), 122.43 (s), 116.95 (s), 116.36 (s), 116.14 (s); $^{19}F$ NMR (376 MHz, $CDCl_3$) δ: -113.69 (s); HRMS (ESI): Calculated for $C_{25}H_{17}ClFN_2O_2$ $[M + H]^+$: 431.0884, found: 431.0942.

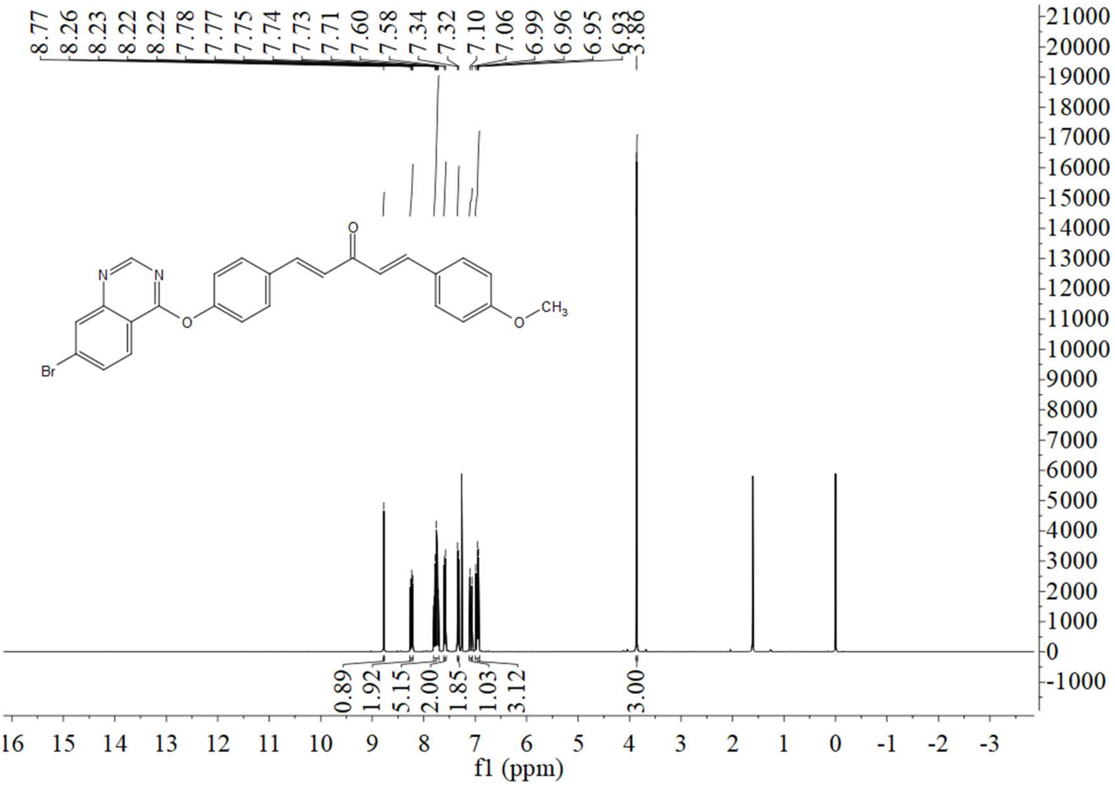


$^{1}H$ NMR of compound **A1.**

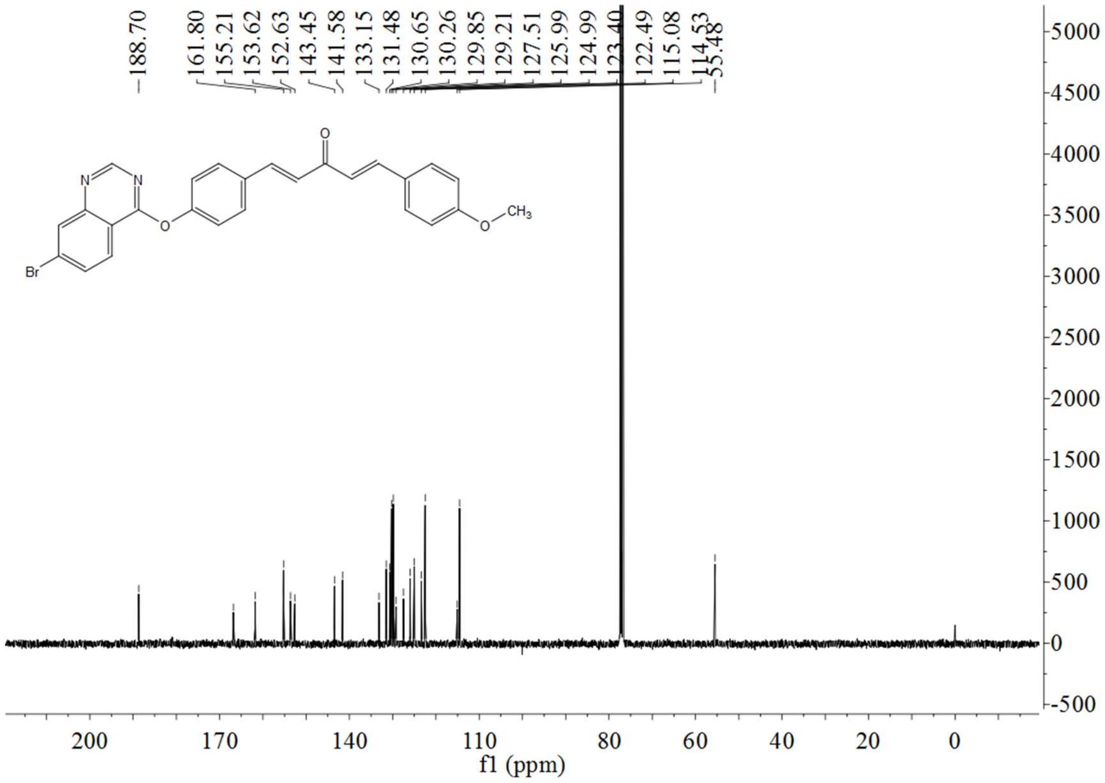


$^{13}$C NMR of compound **A1.**

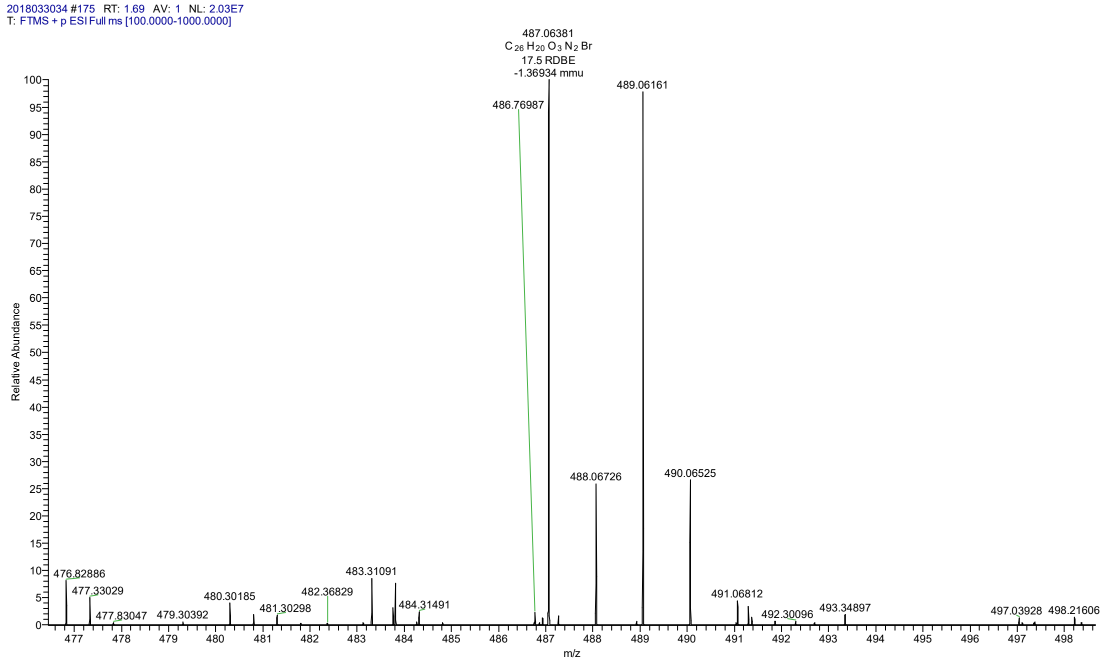


HRMS of compound **A1.**

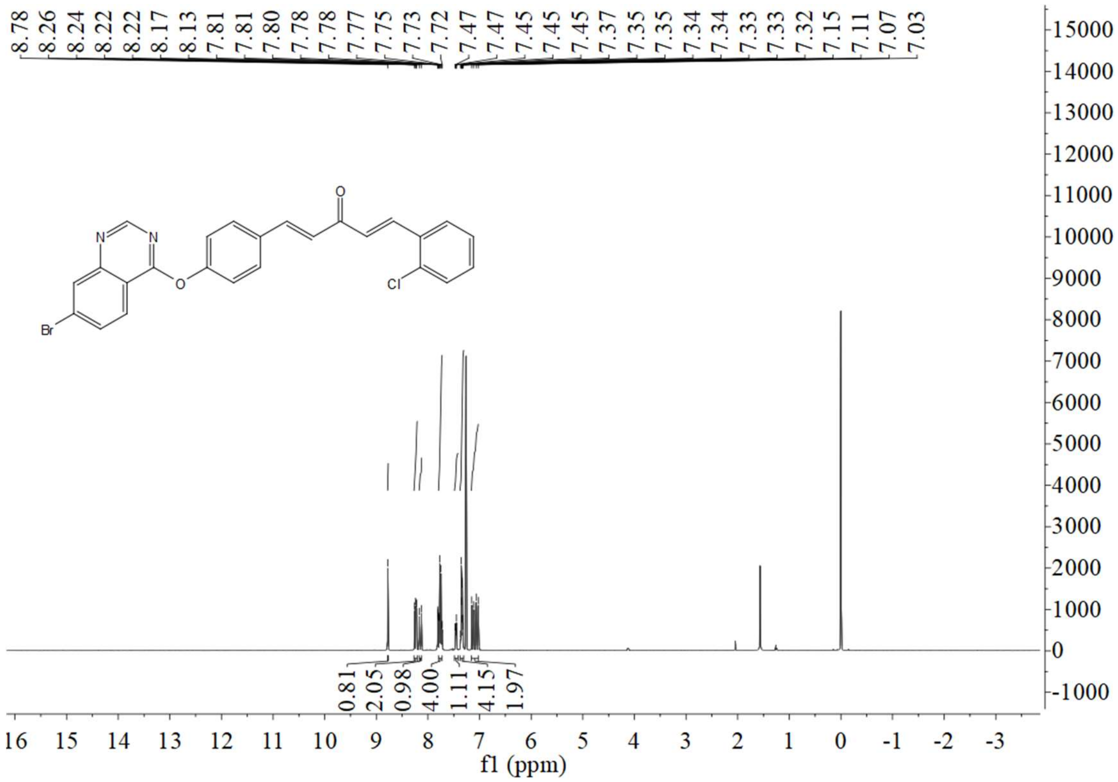


$^{1}$H NMR of compound **A2.**

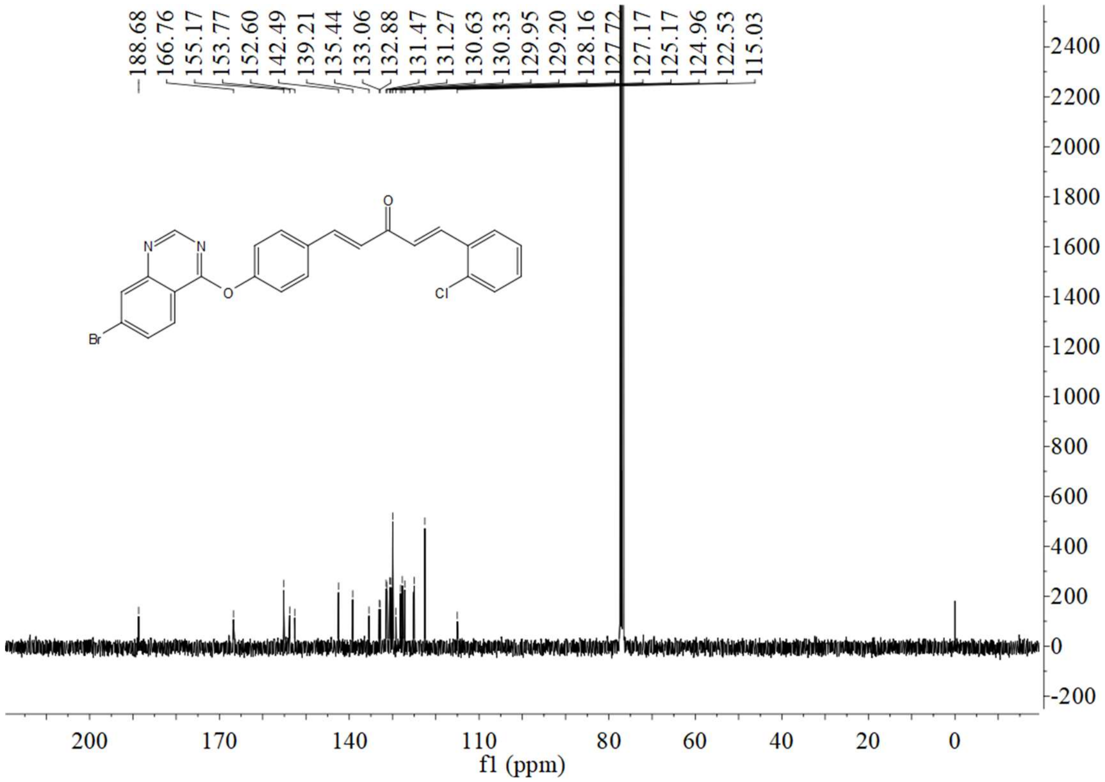


$^{13}$C NMR of compound **A2.**

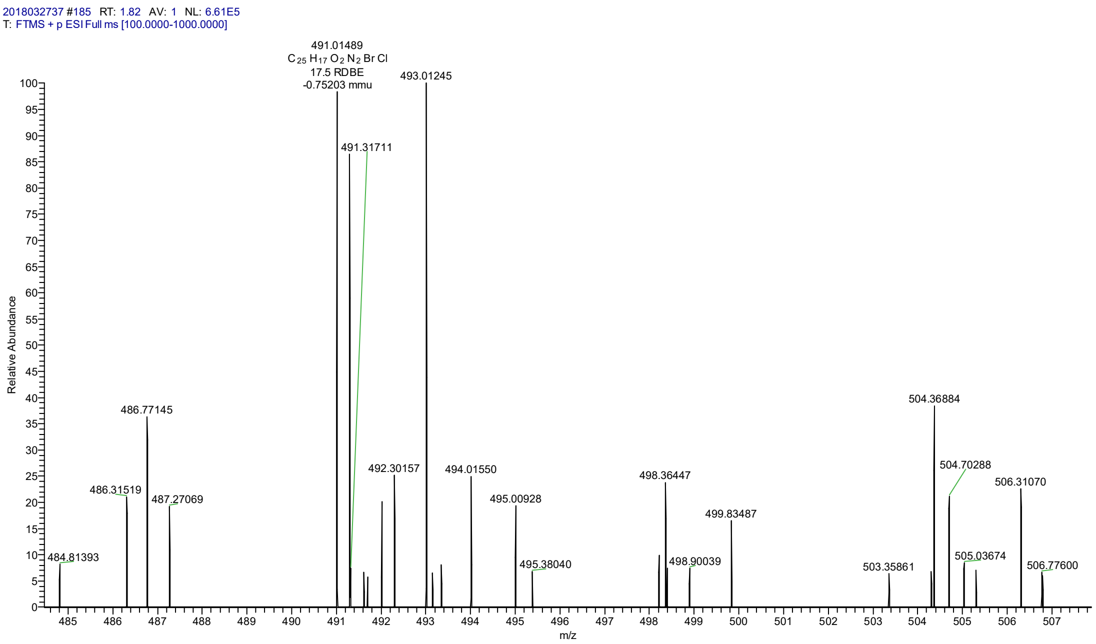


HRMS of compound **A2.**

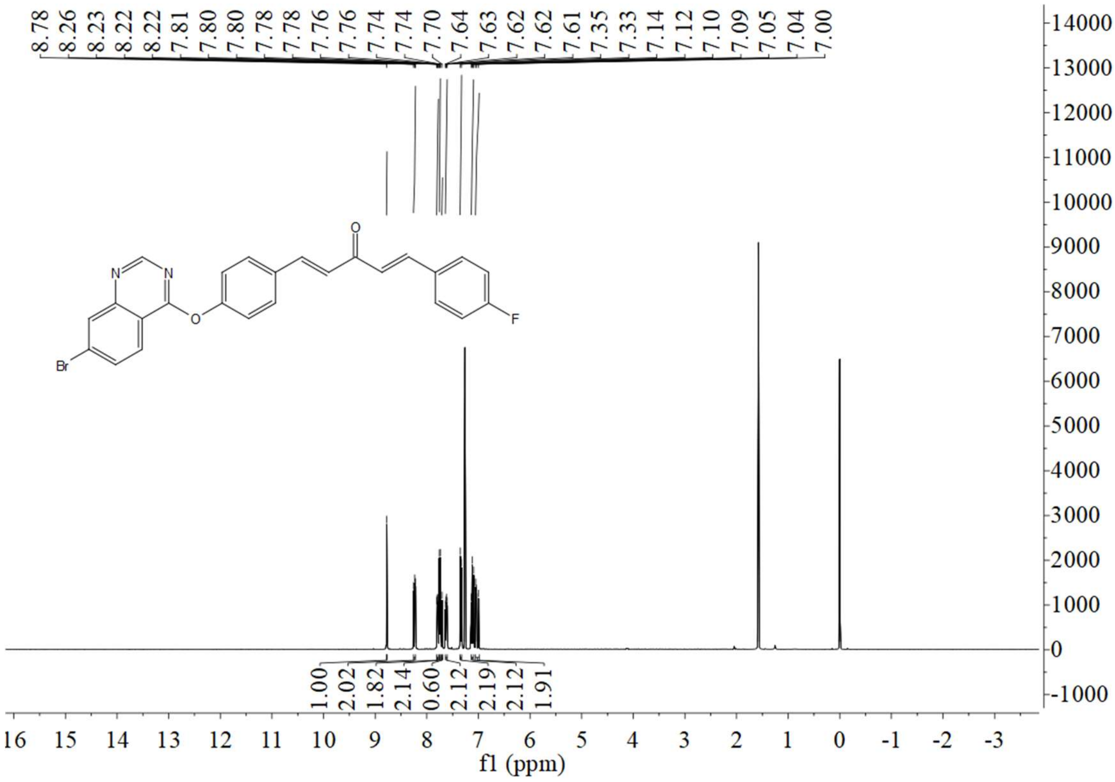


$^{1}$H NMR of compound **A3.**

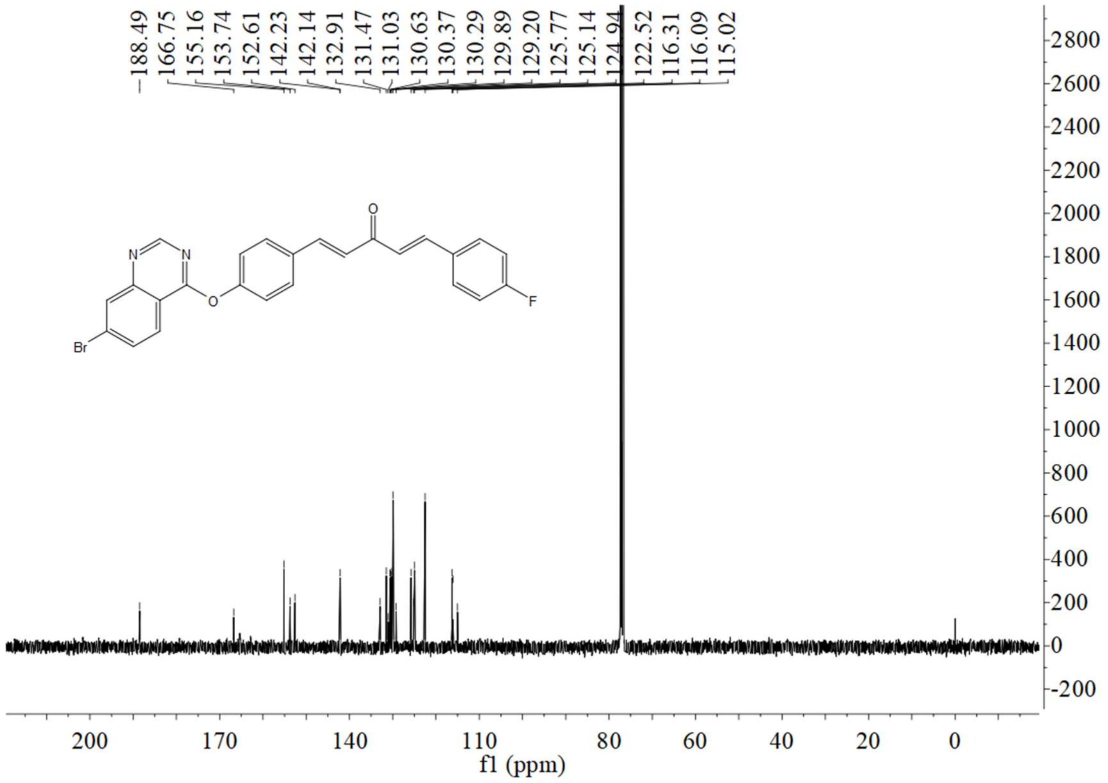

$^{13}$C NMR of compound **A3.**

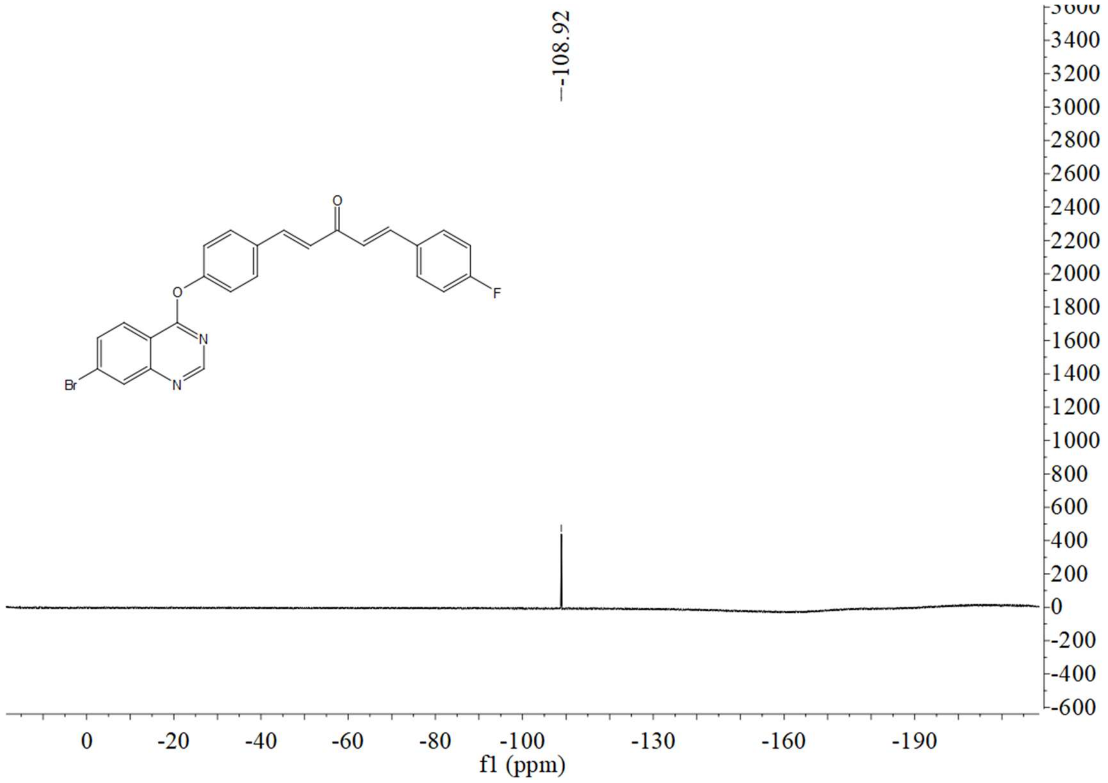

$^{19}$F NMR of compound **A3.**

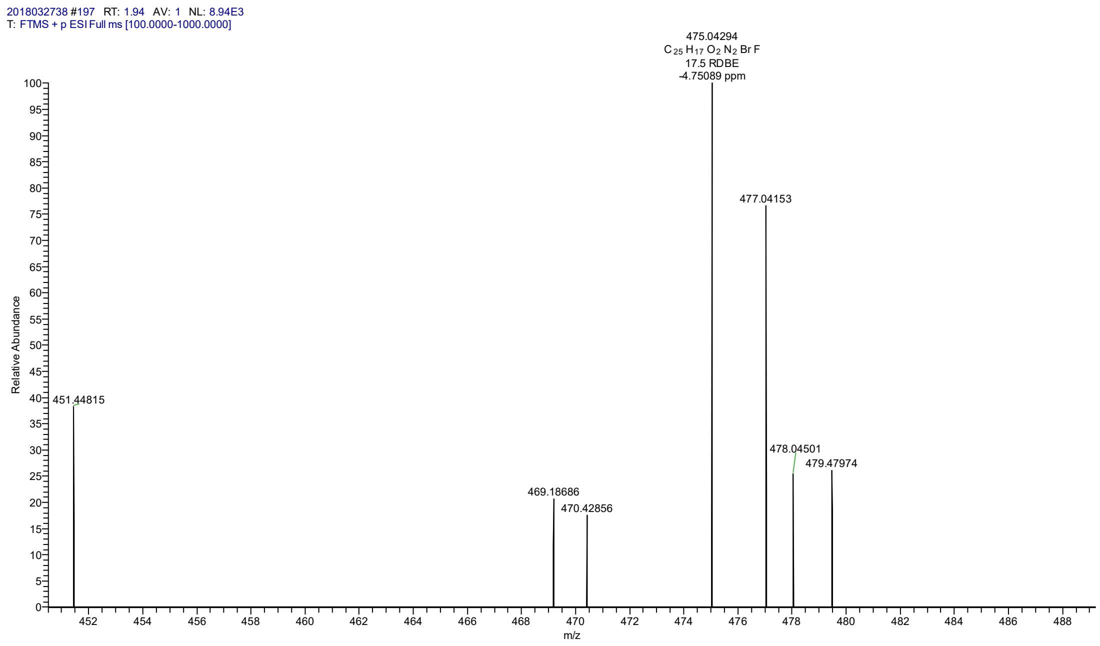


HRMS of compound **A3.**

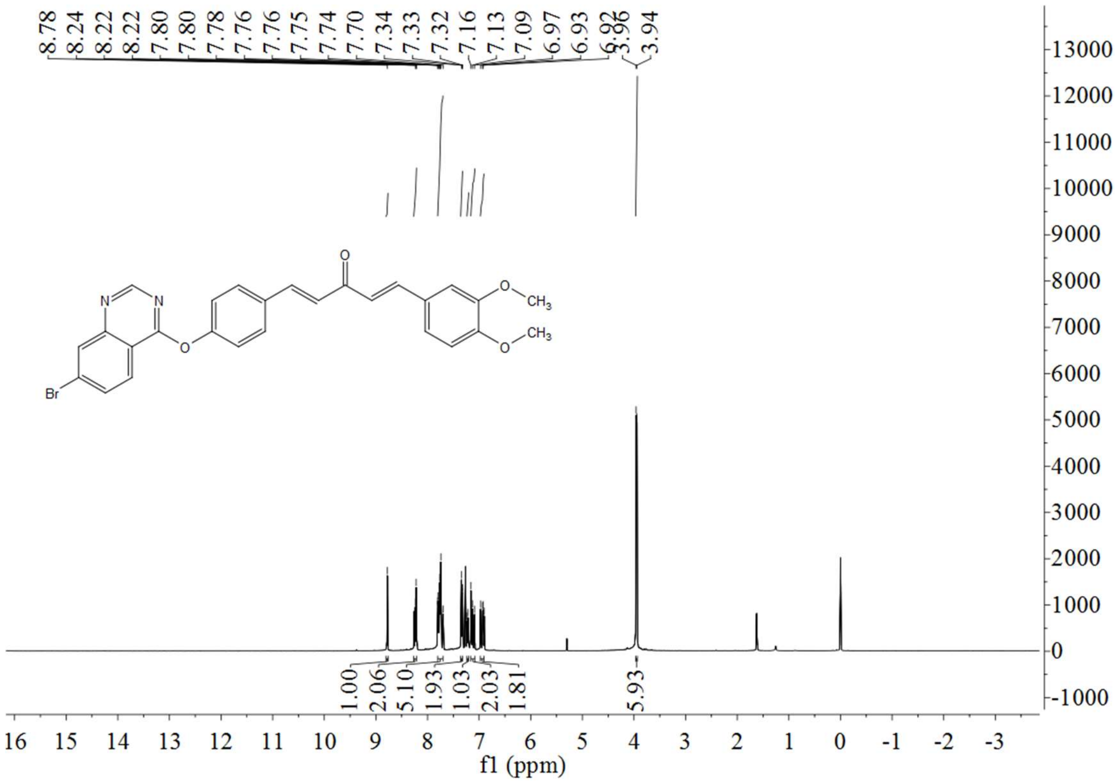


$^{1}$H NMR of compound **A4.**

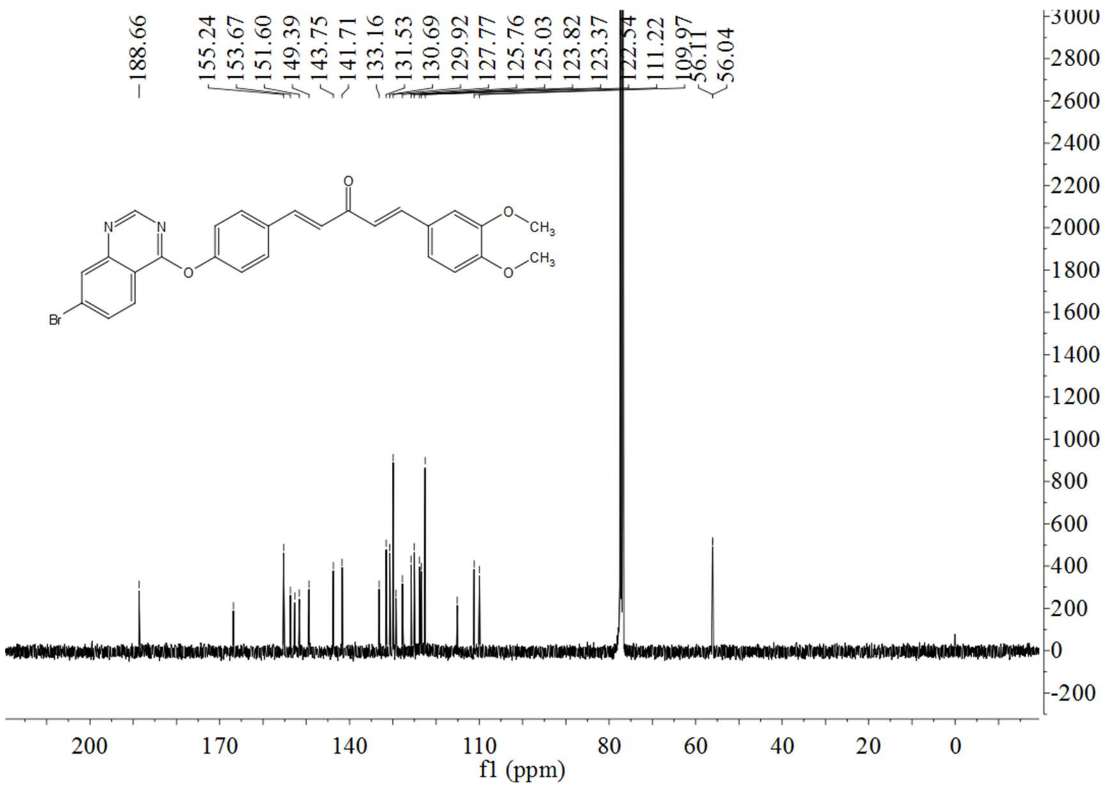


$^{13}$C NMR of Compound **A4.**

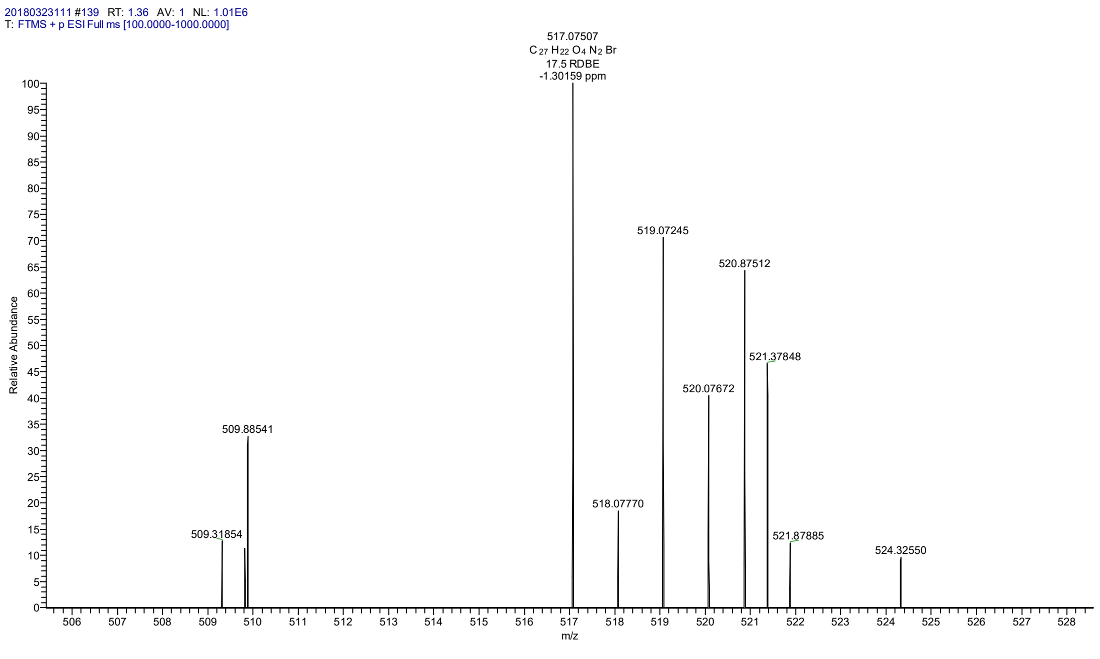


HRMS of compound **A4.**

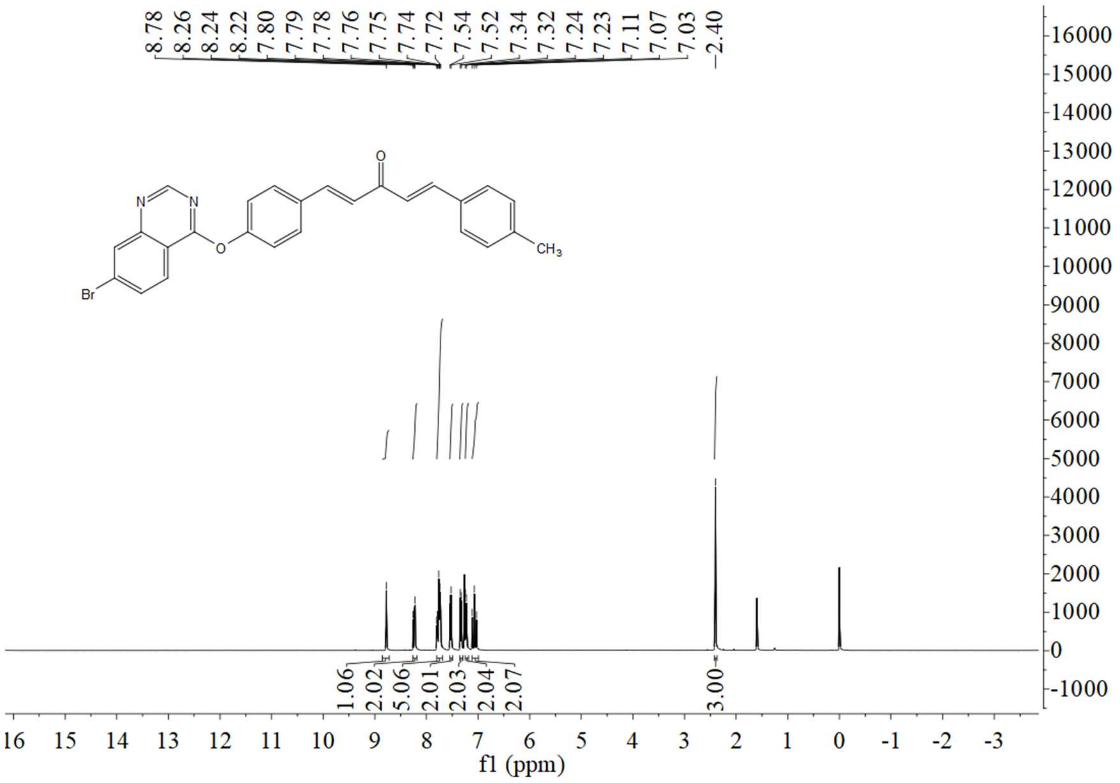

$^{1}$H NMR of compound **A5.**

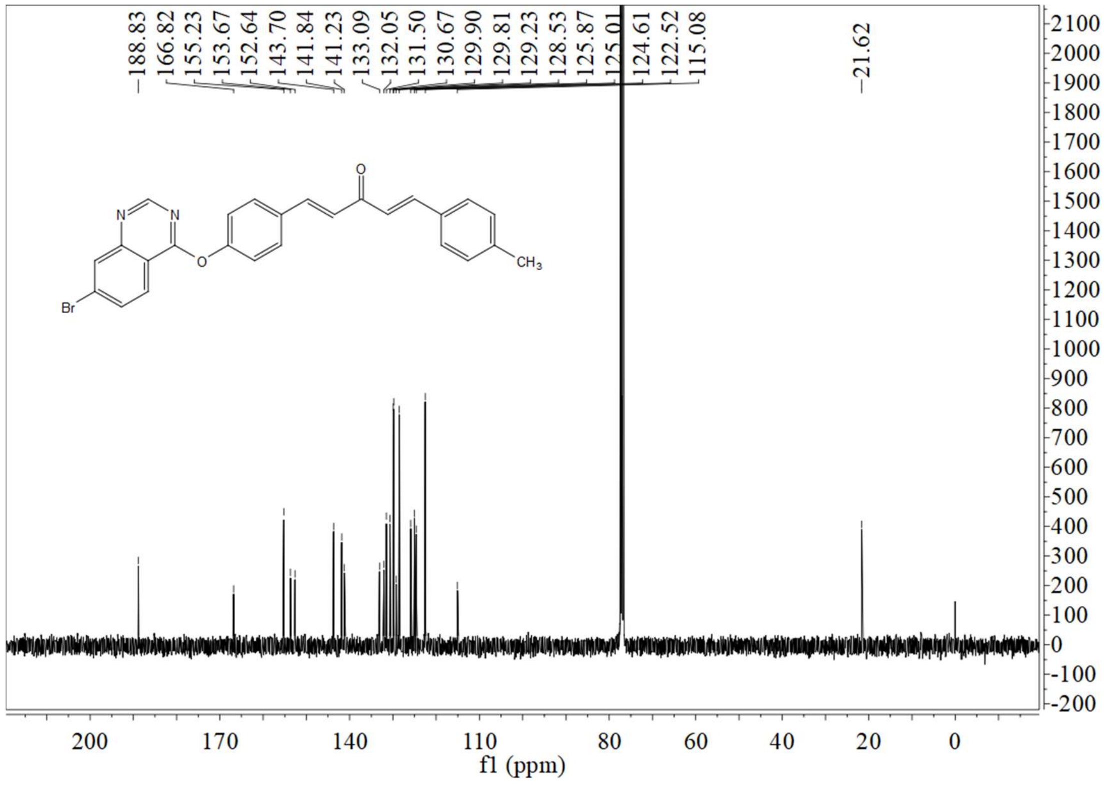

$^{13}$C NMR of compound **A5.**

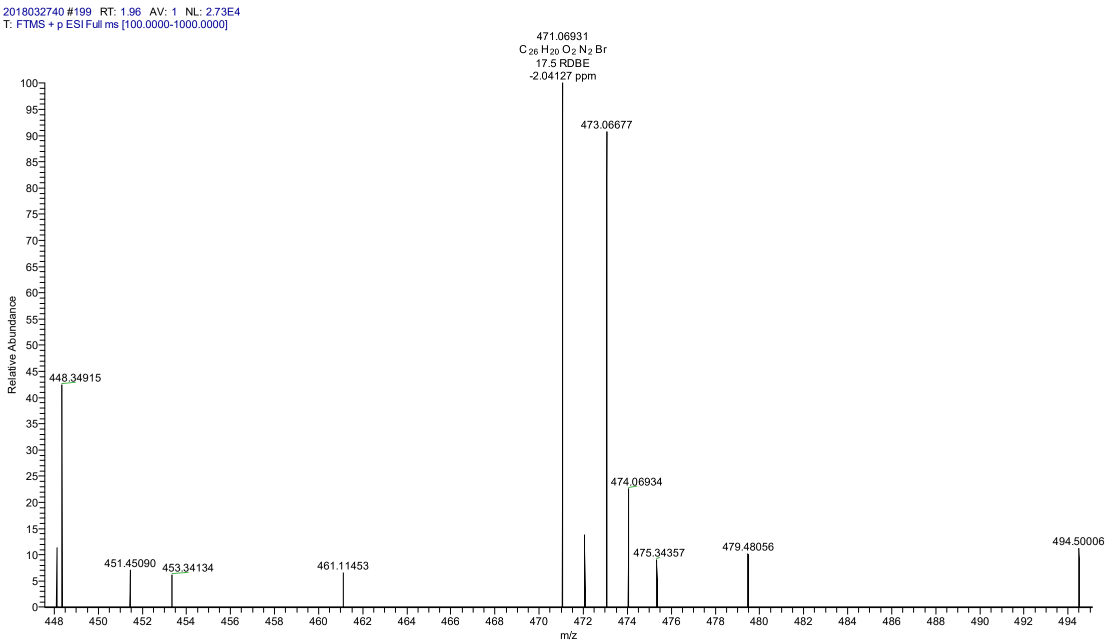


HRMS of compound **A5.**

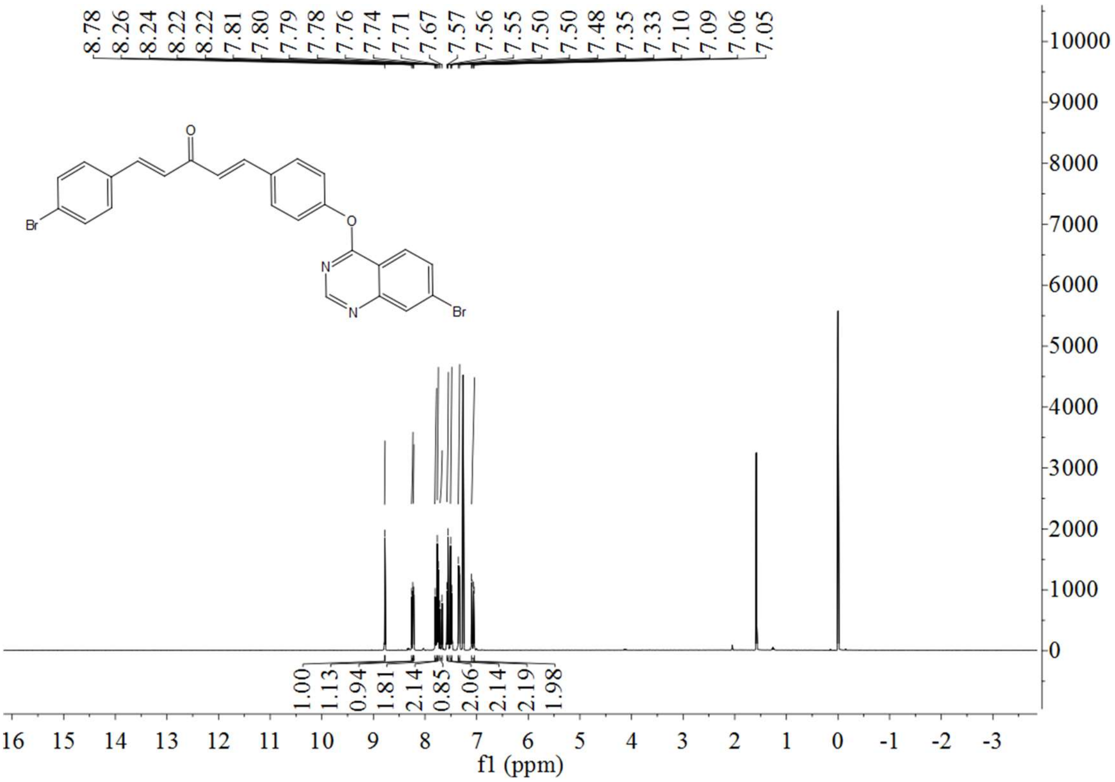


$^{1}$H NMR of compound **A6.**

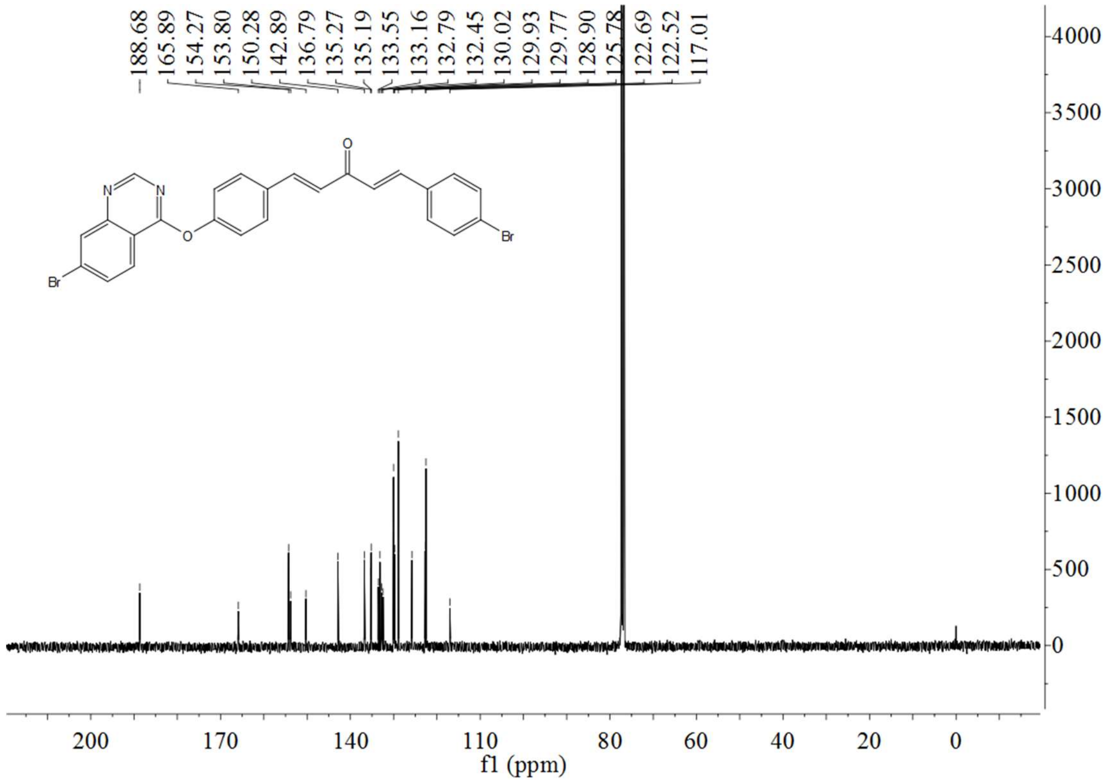


$^{13}$C NMR of compound **A6.**

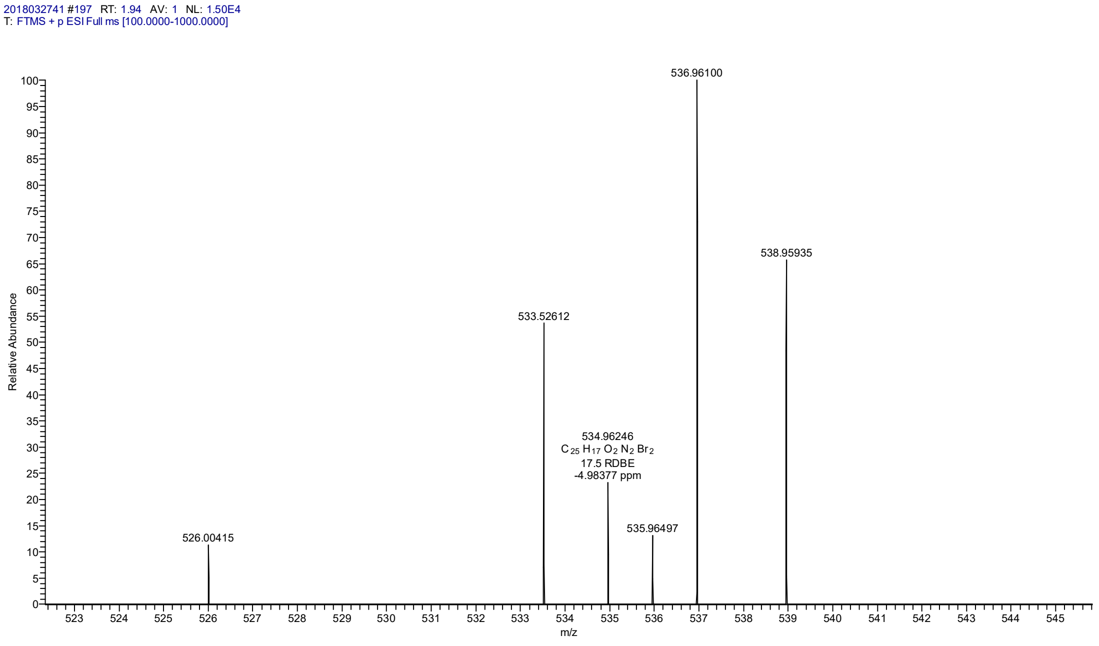


HRMS of compound **A6.**

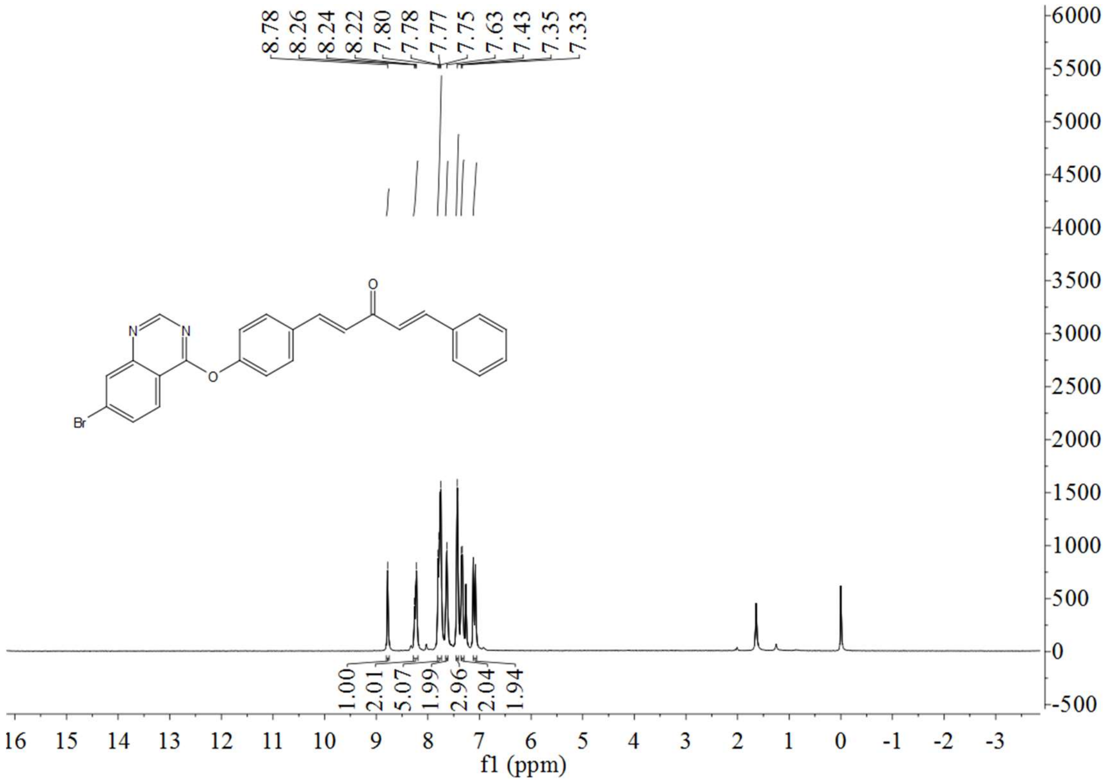


$^{1}$H NMR of compound **A7.**

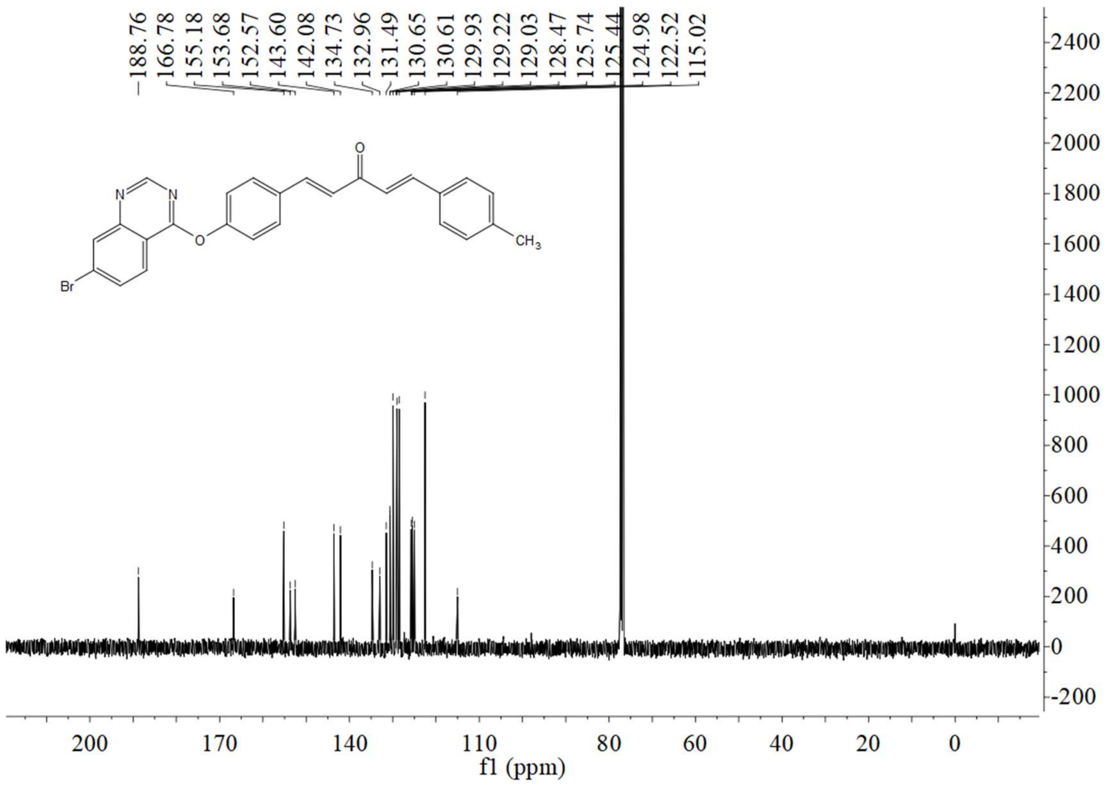


$^{13}$C NMR of compound **A7.**

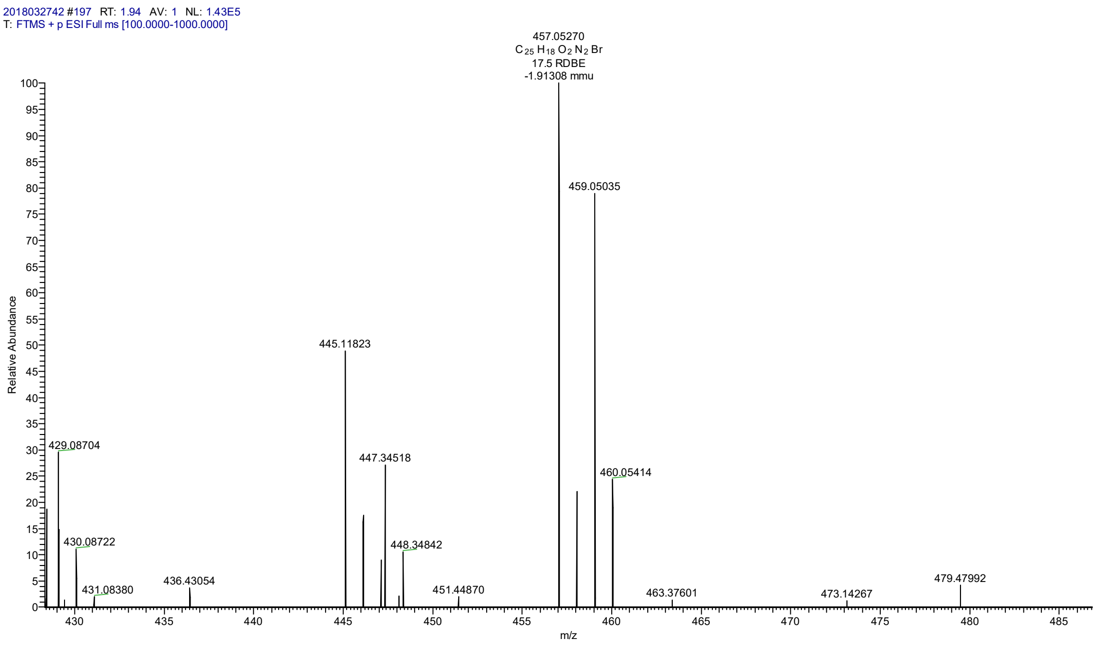


HRMS of compound **A7.**

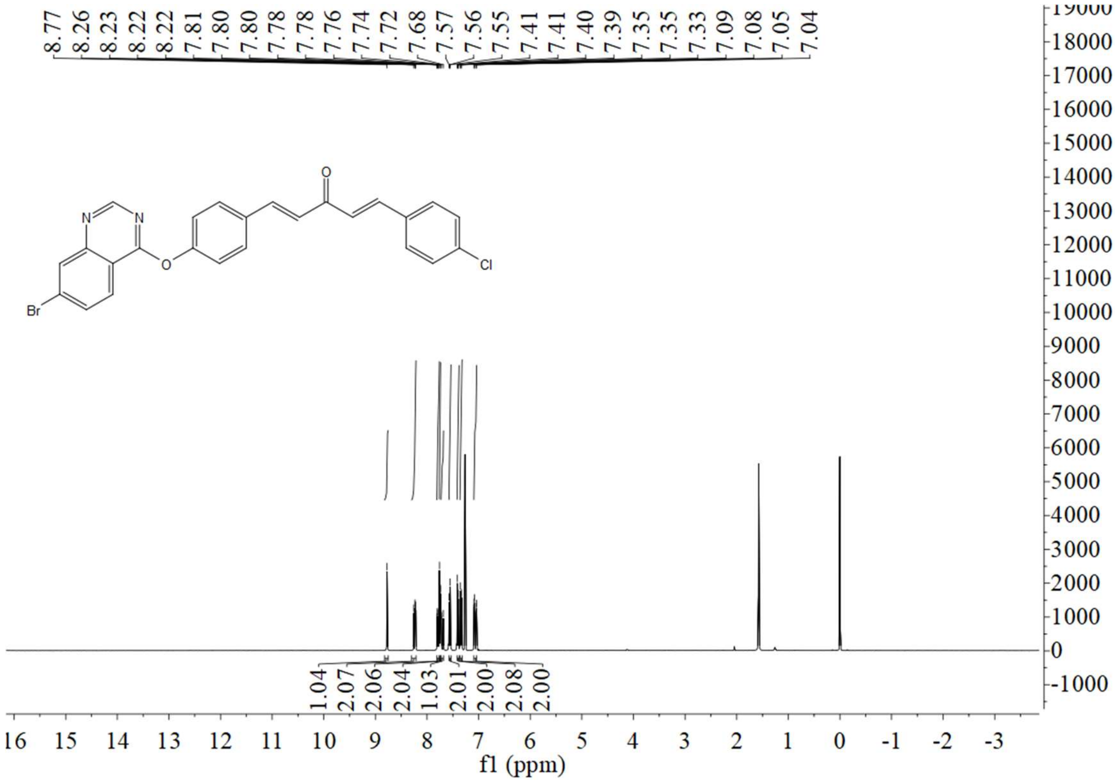


$^{1}$H NMR of compound **A8.**

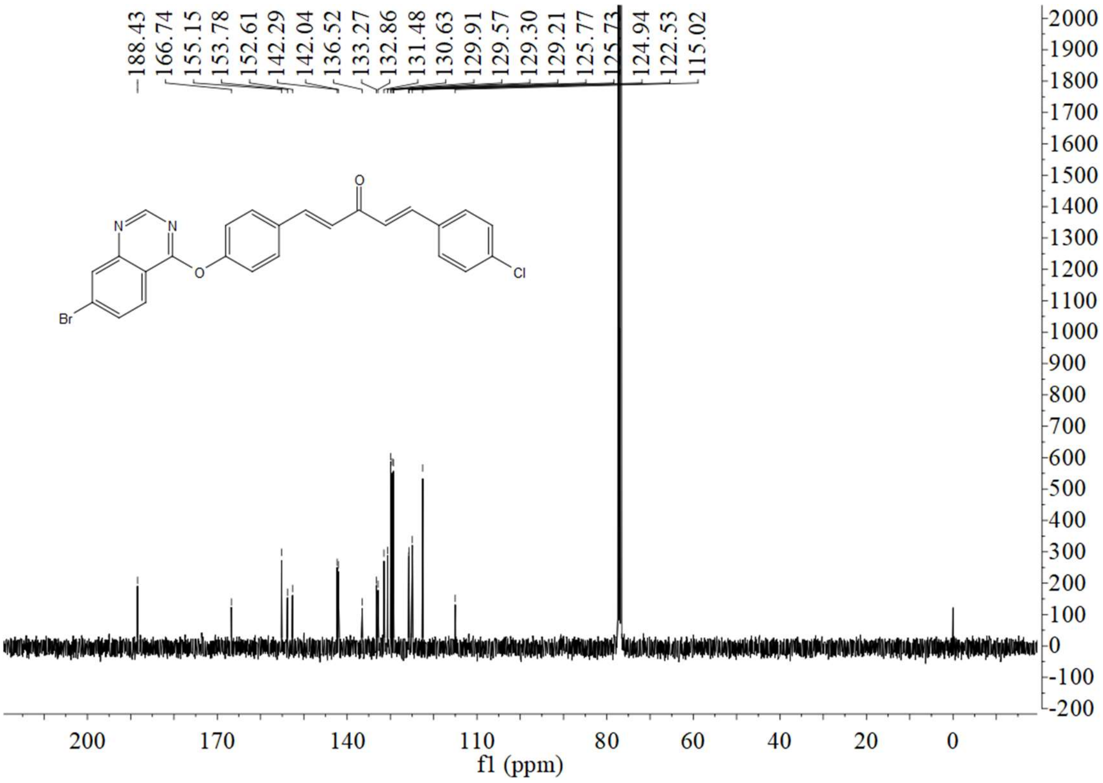


$^{13}C$ NMR of compound **A8.**

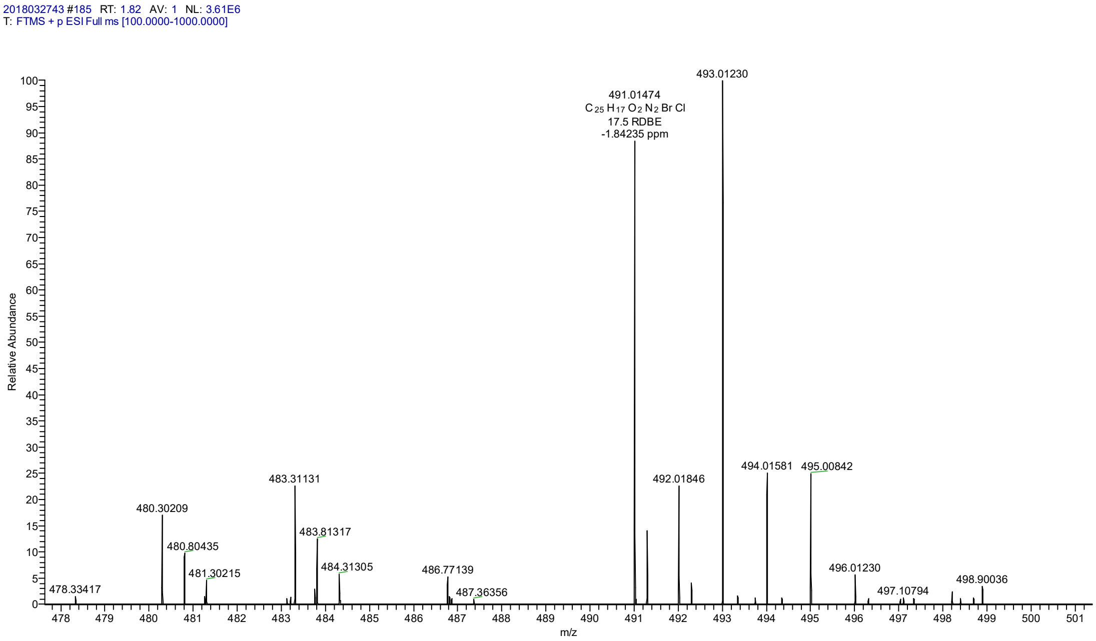


HRMS of compound **A8.**

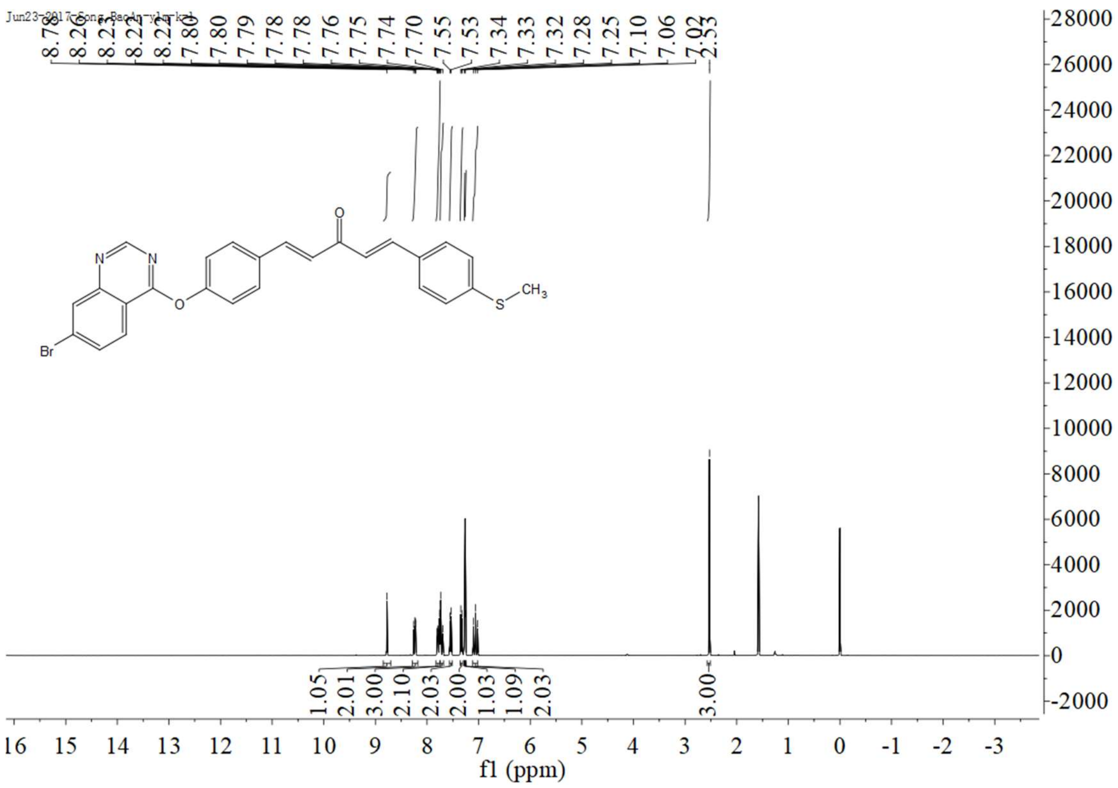

$^{1}$H NMR of compound **A9.**

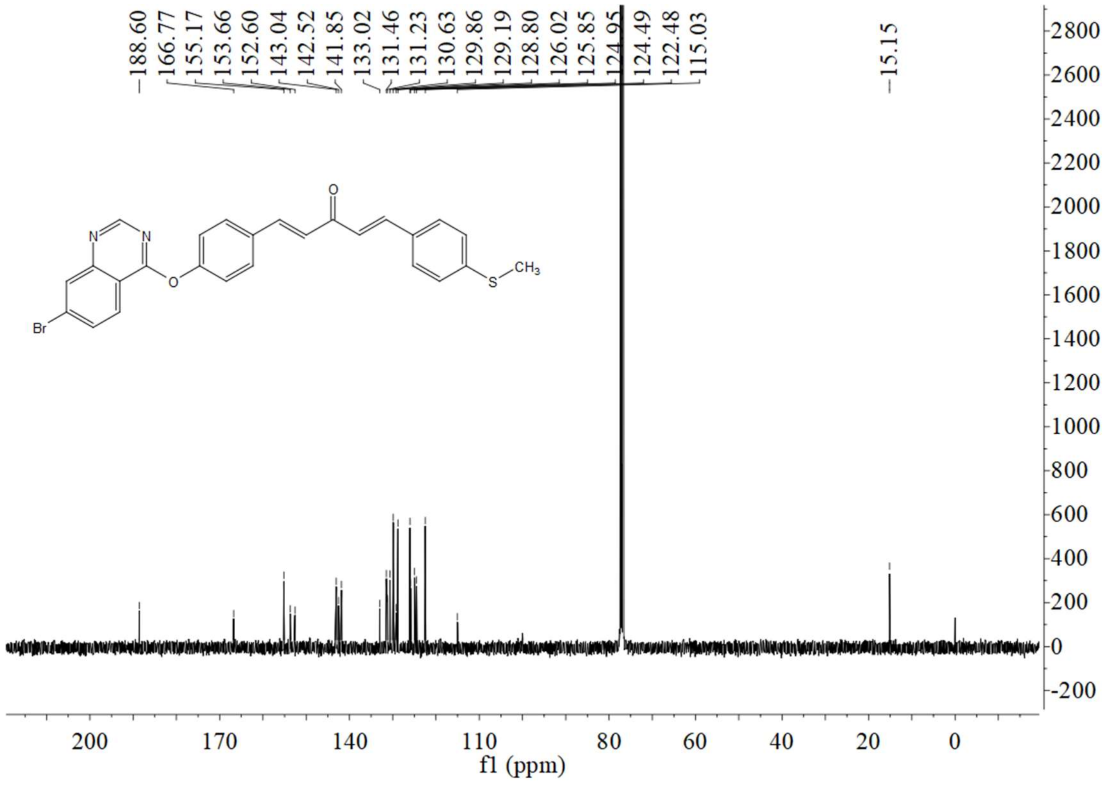

$^{13}$C NMR of compound **A9.**

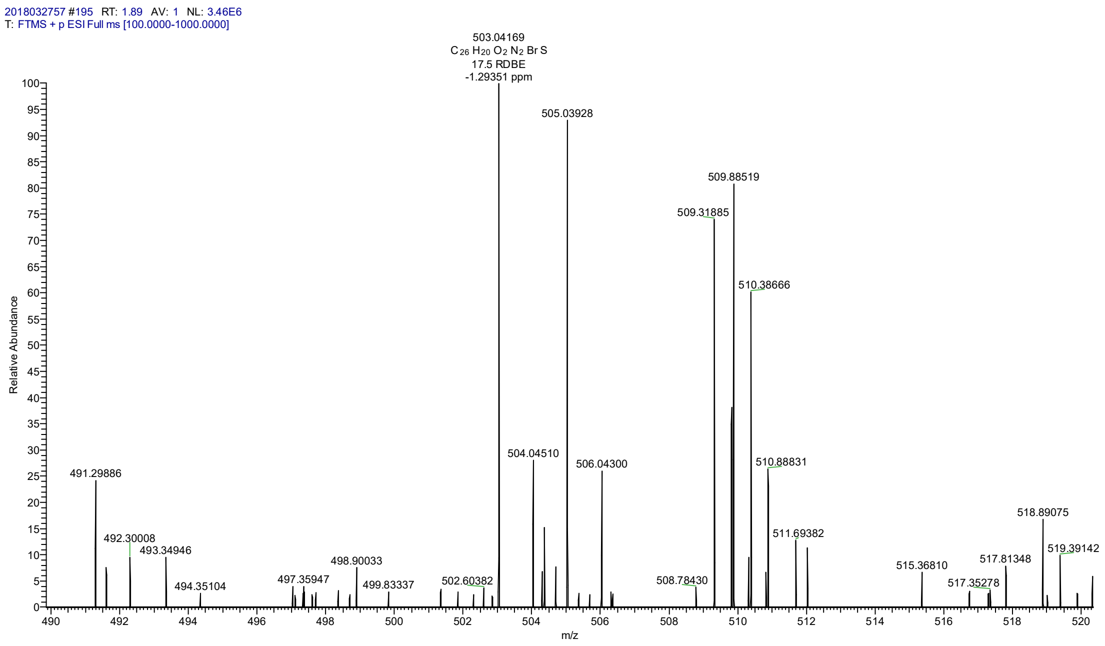


HRMS of compound **A9.**

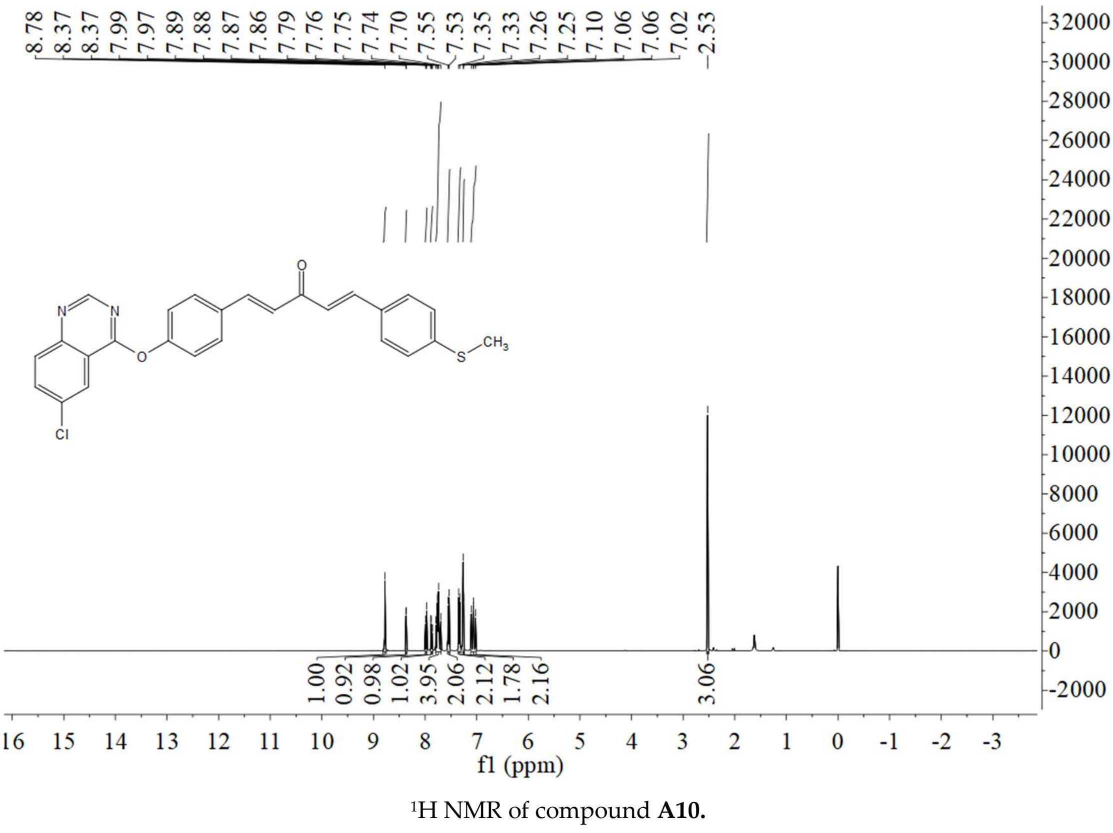


$^{1}$H NMR of compound **A10.**

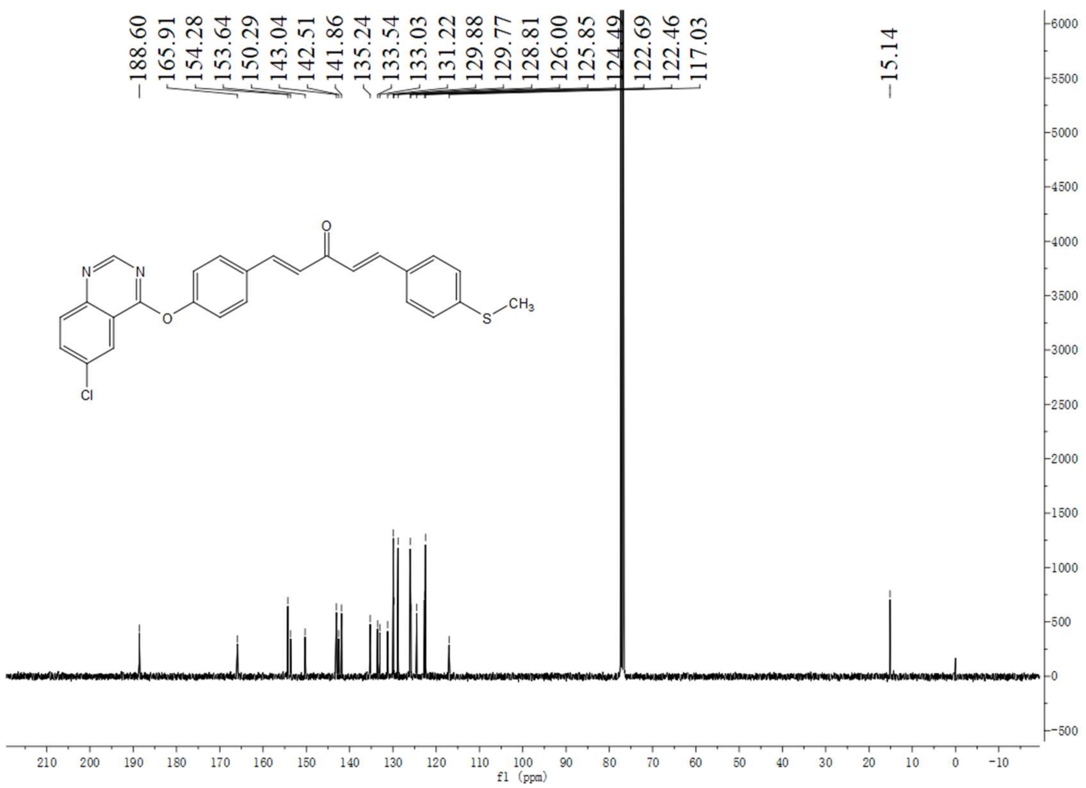


$^{13}$C NMR of compound **A10.**

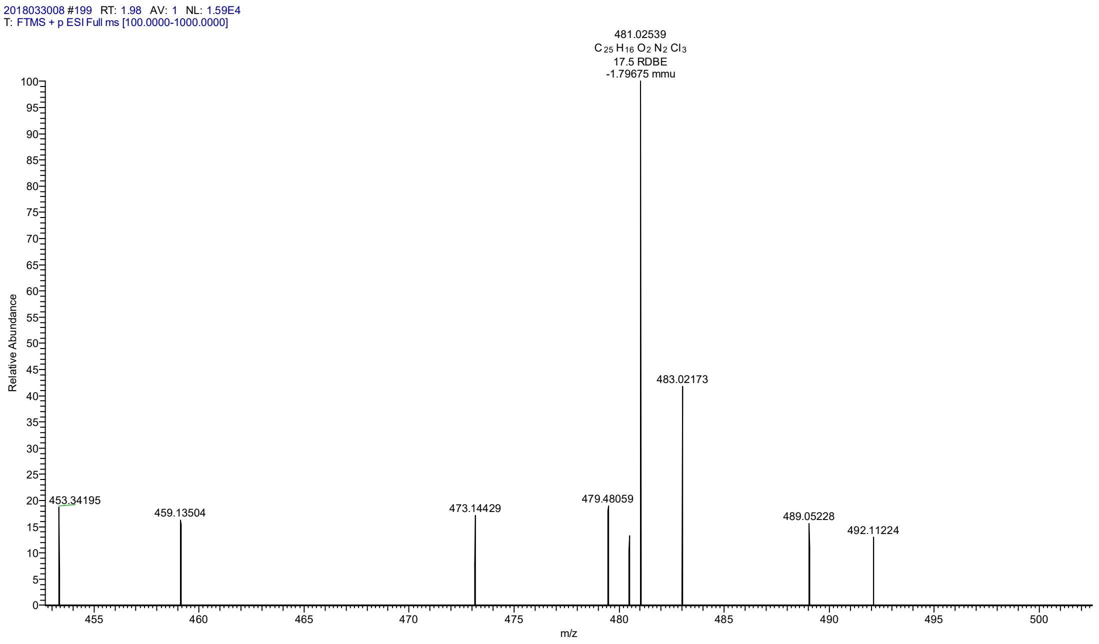


HRMS of compound **A10.**

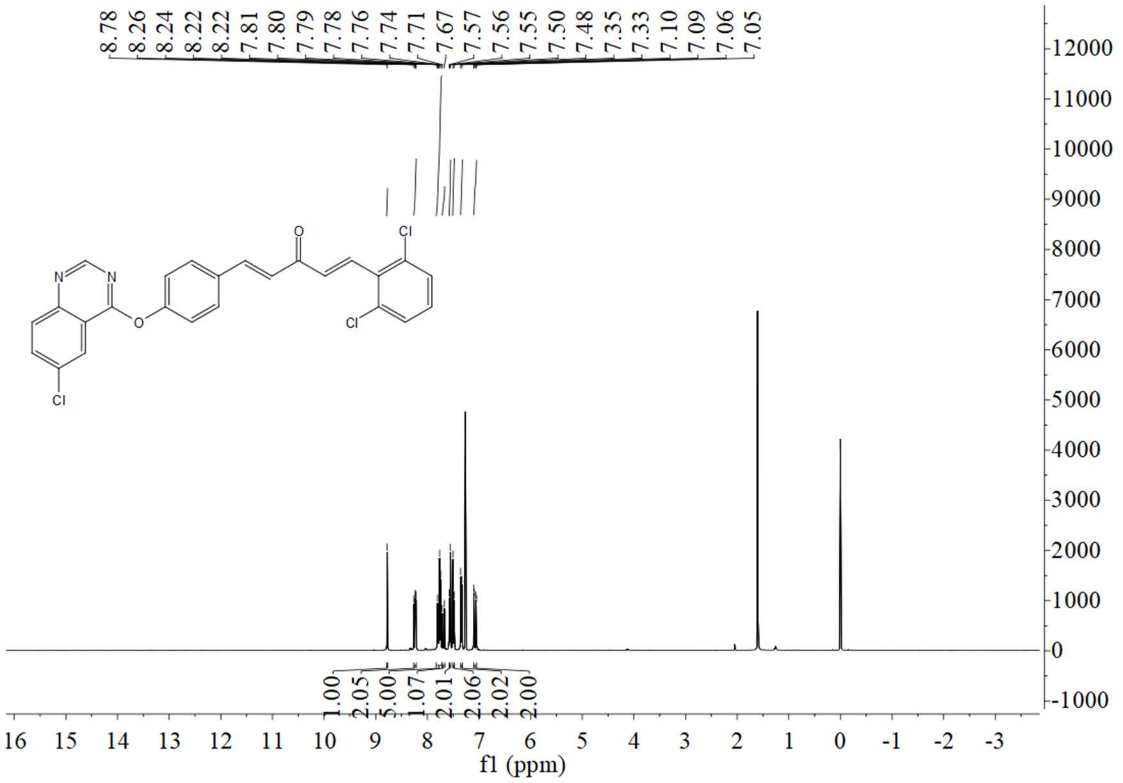


$^{1}$H NMR of compound **A11.**

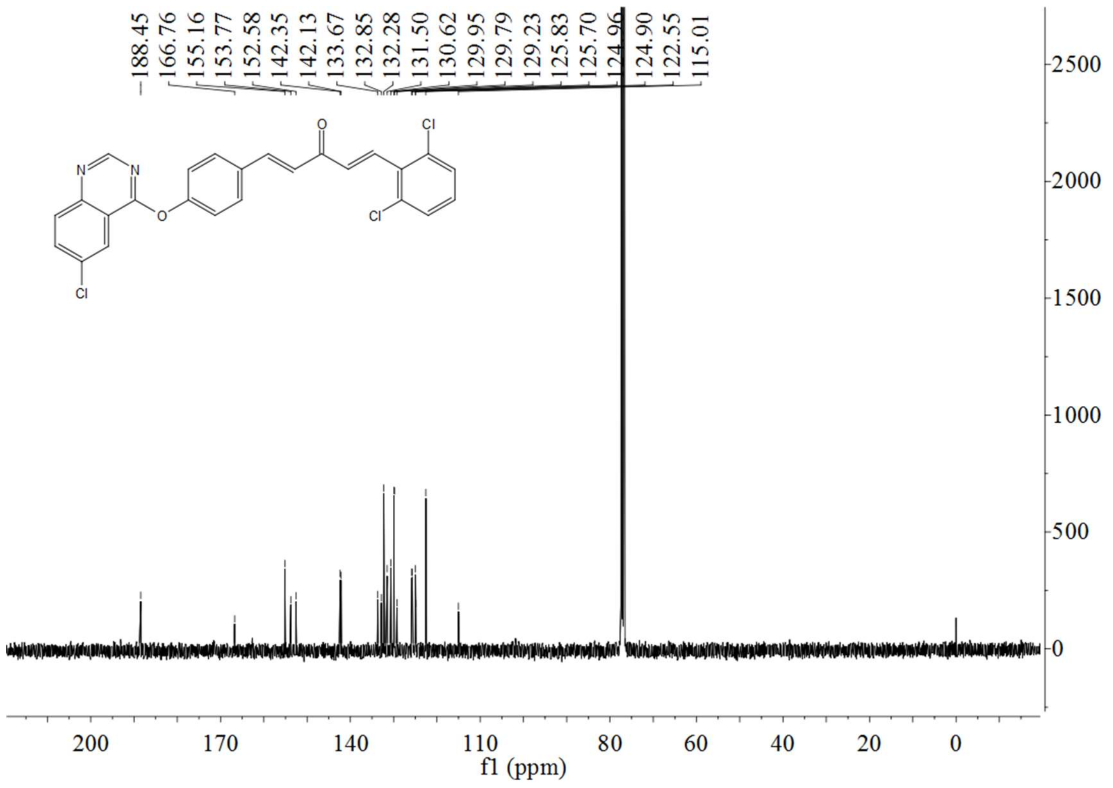


$^{13}$C NMR of compound **A11.**

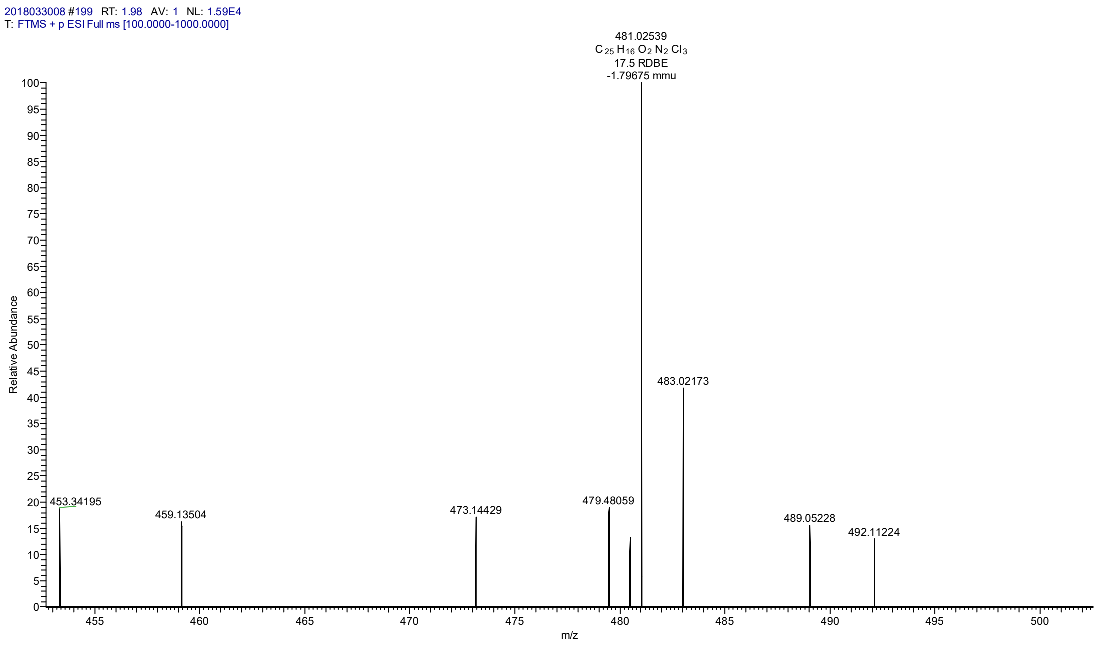


HRMS of compound **A11.**

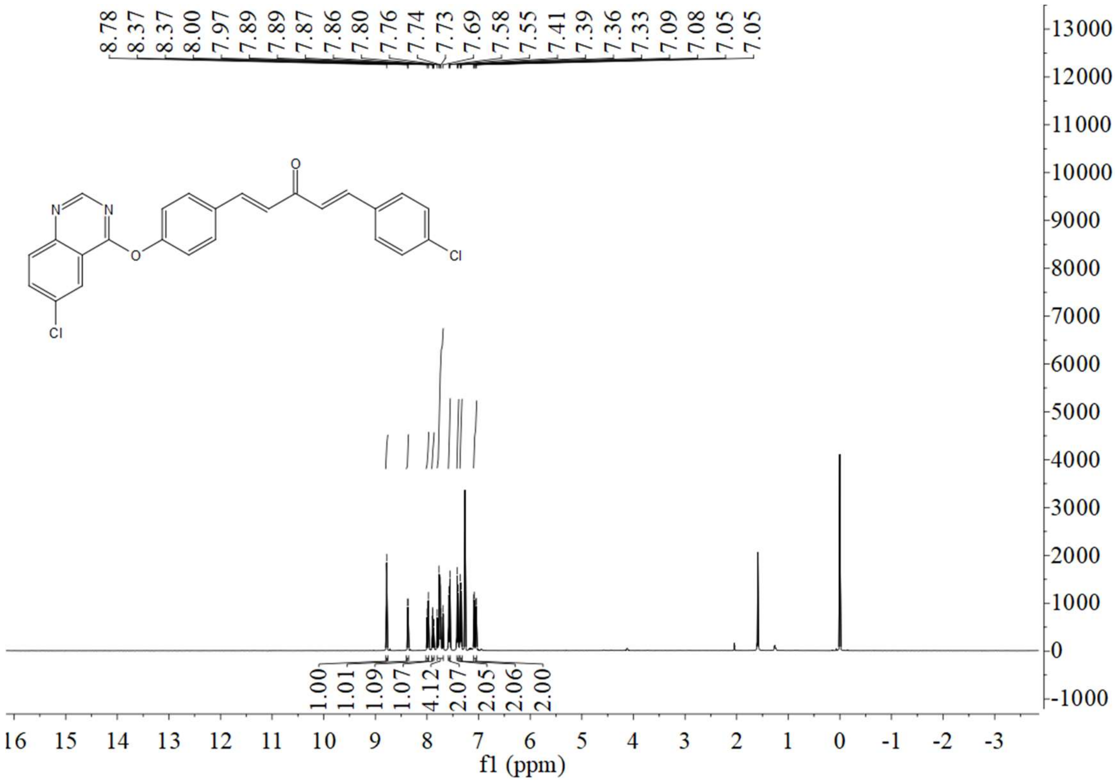


$^{1}$H NMR of compound **A12.**

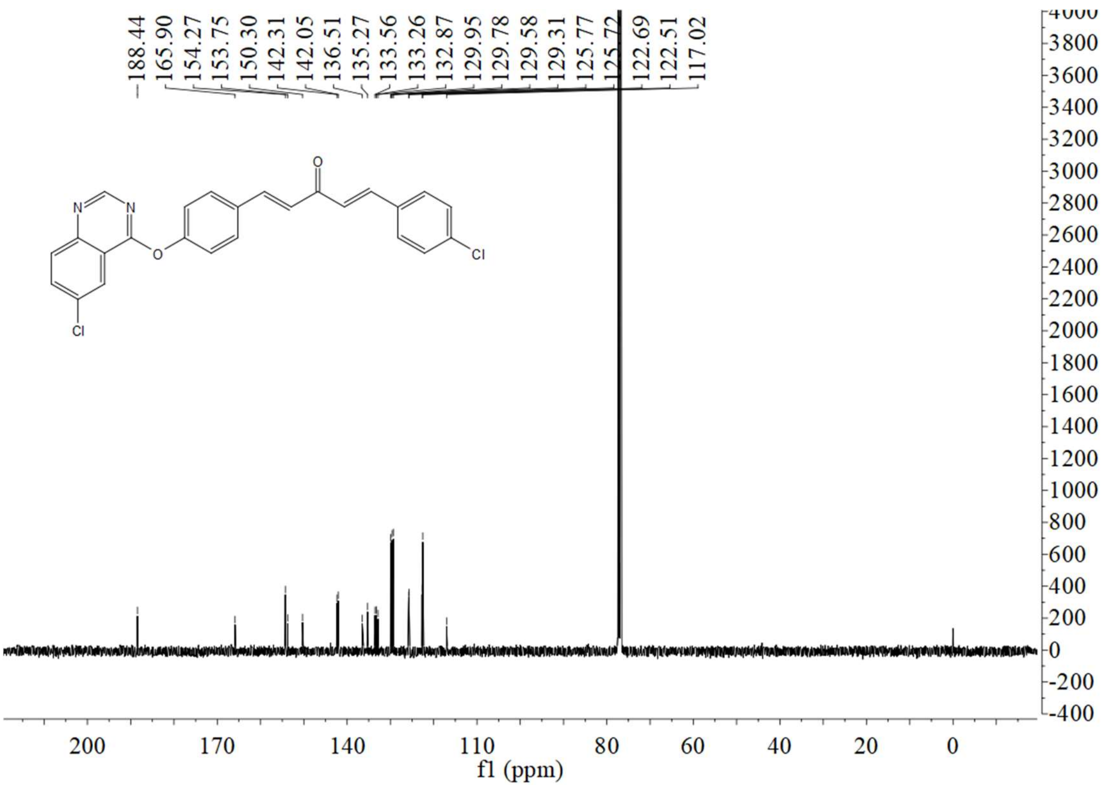


$^{13}C$ NMR of compound **A12.**

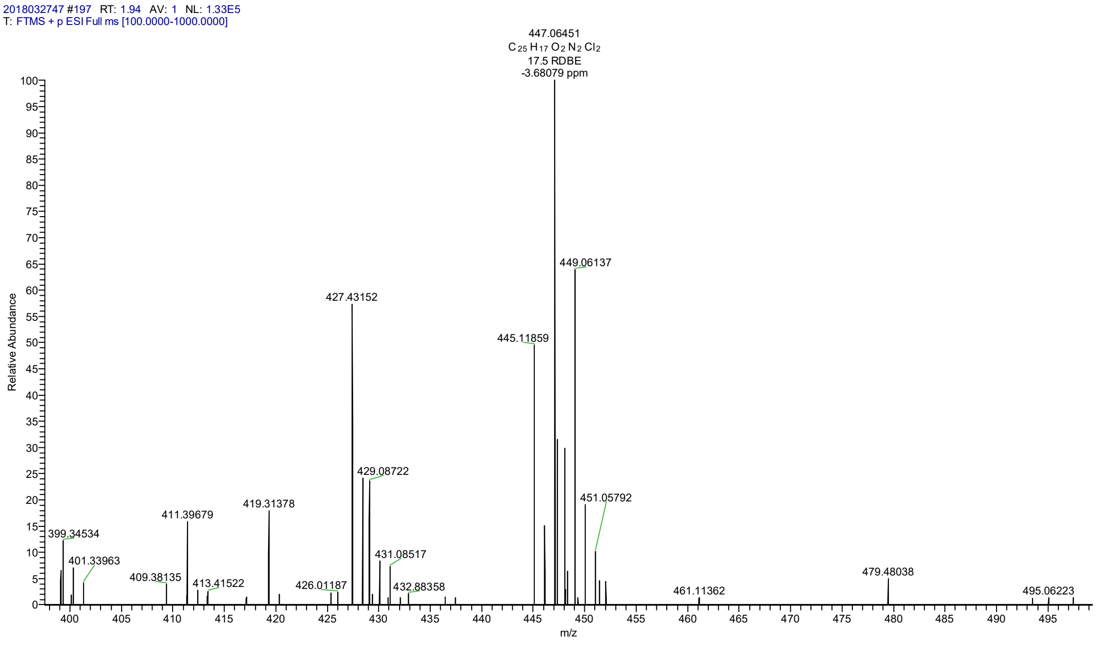


HRMS of compound **A12.**

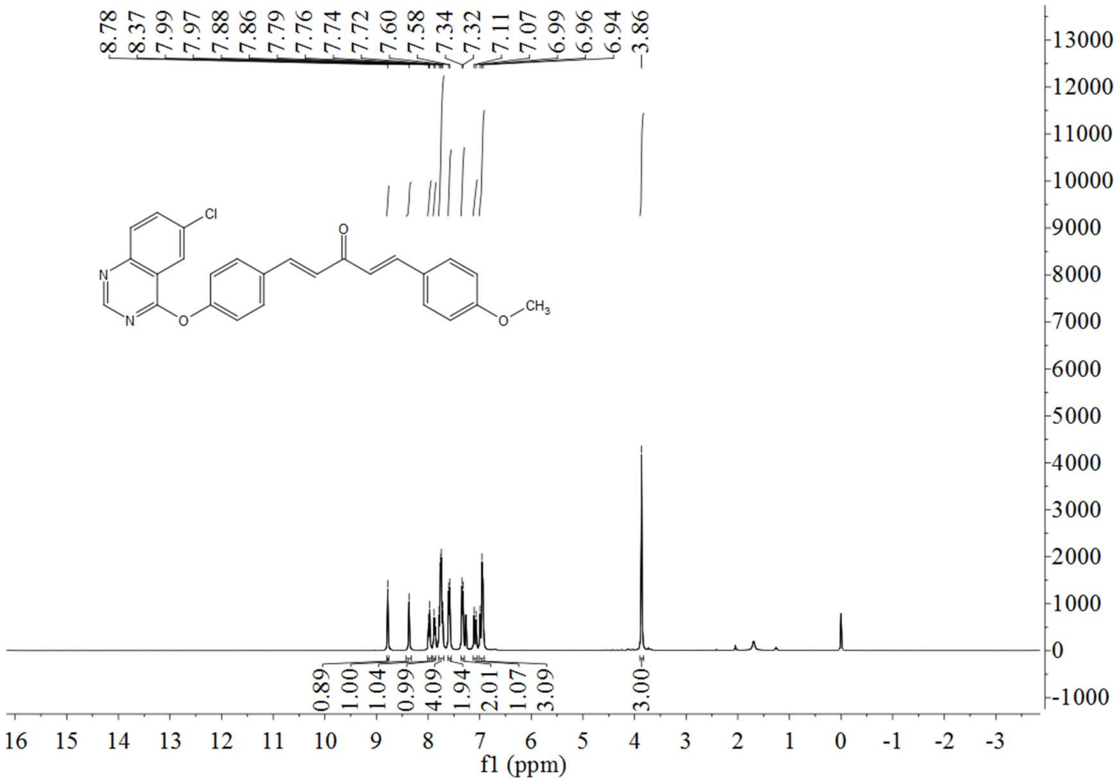

$^{1}$H NMR of compound **A13.**

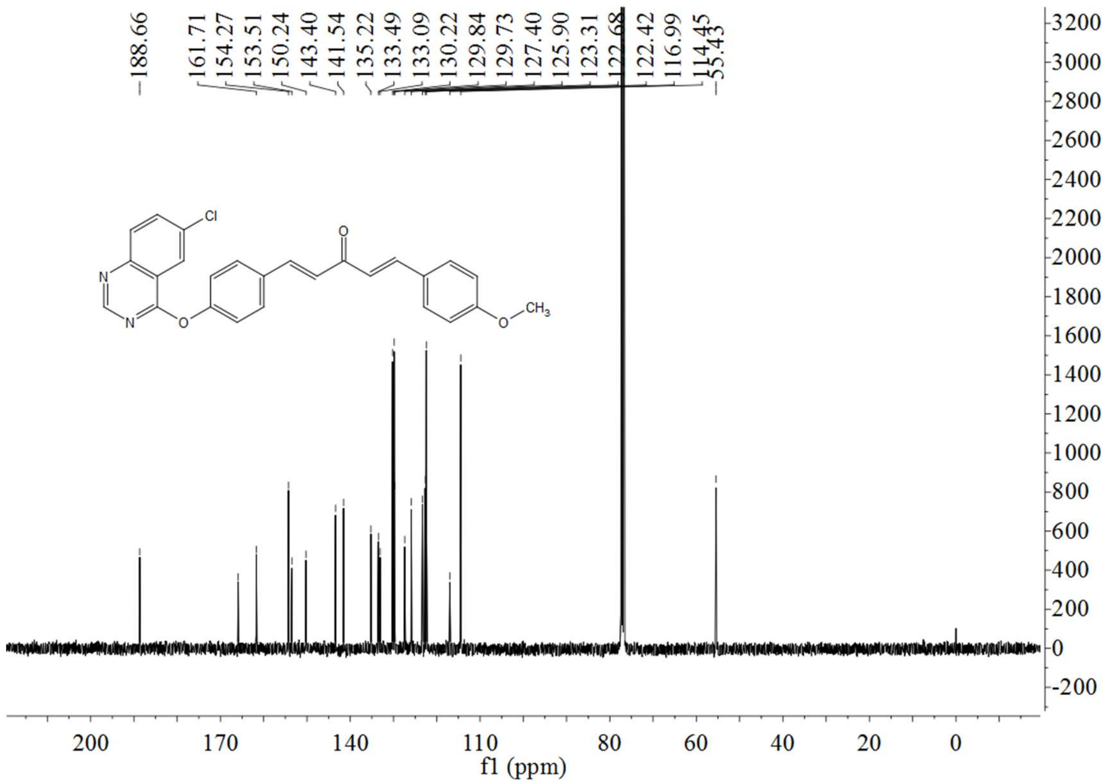

$^{13}$C NMR of compound **A13.**

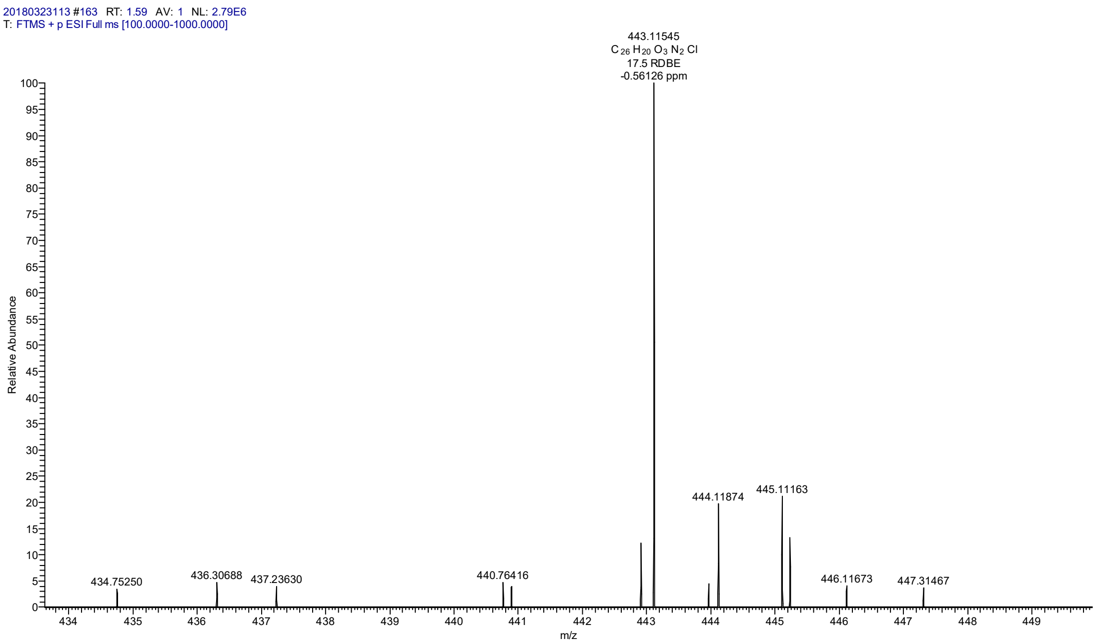


HRMS of compound **A13.**

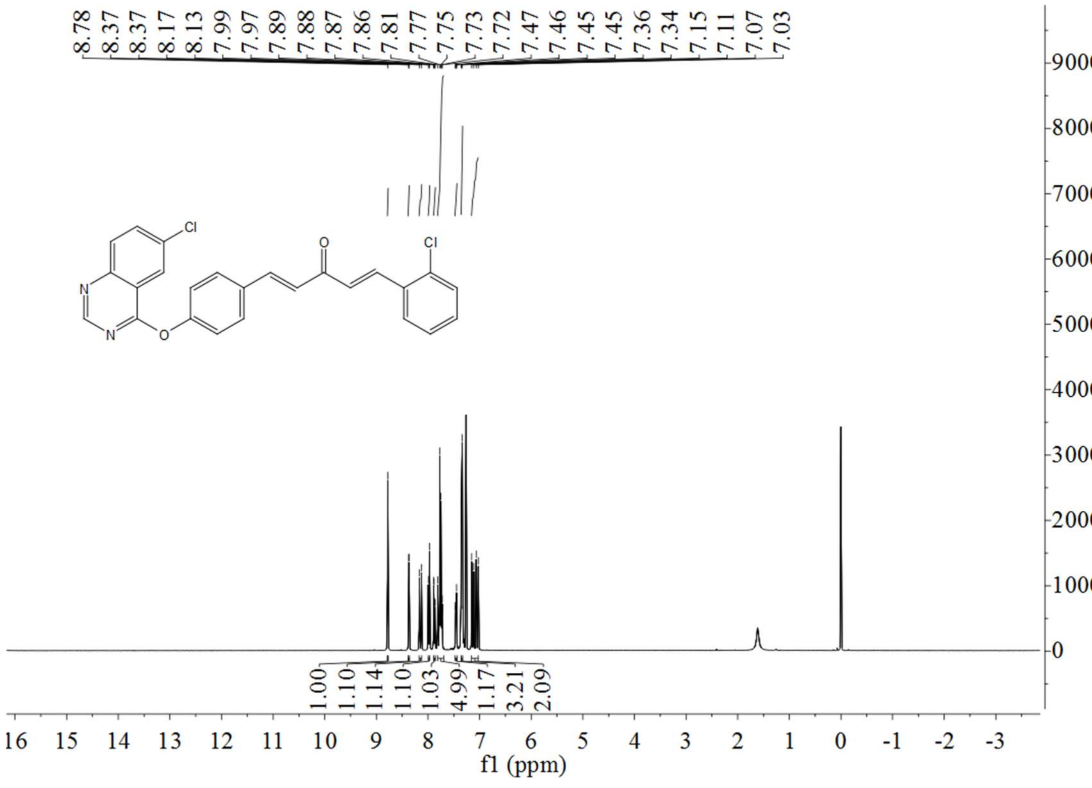


$^{1}$H NMR of compound **A14.**

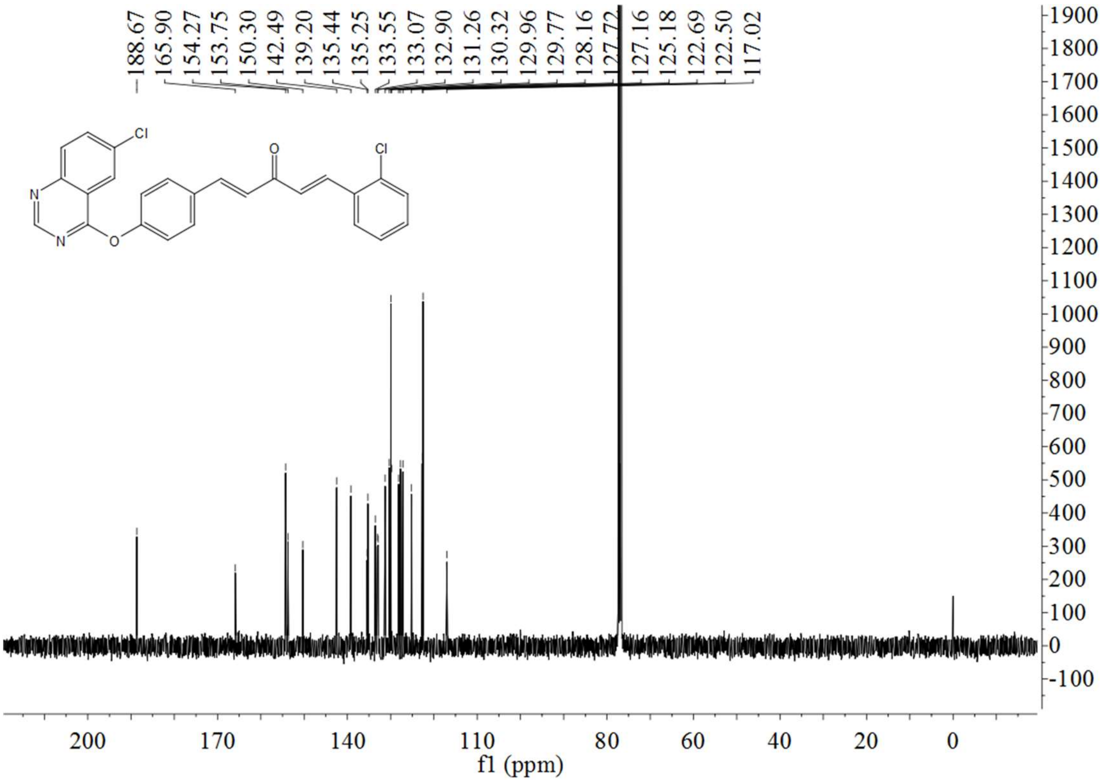


$^{13}$C NMR of compound **A14.**

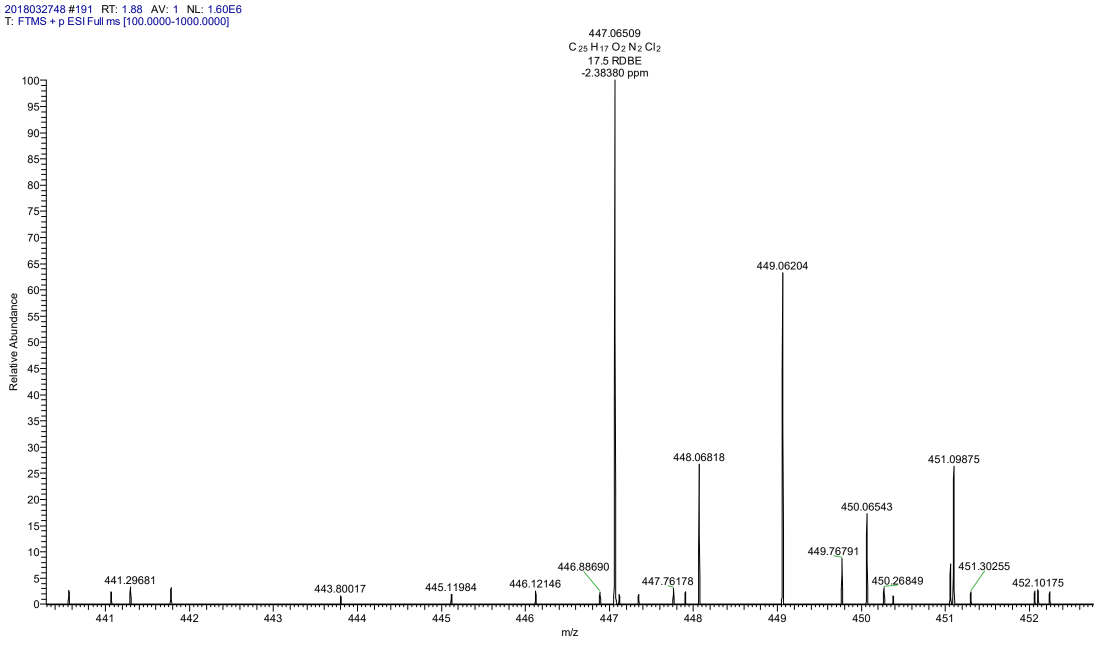


HRMS of compound **A14.**

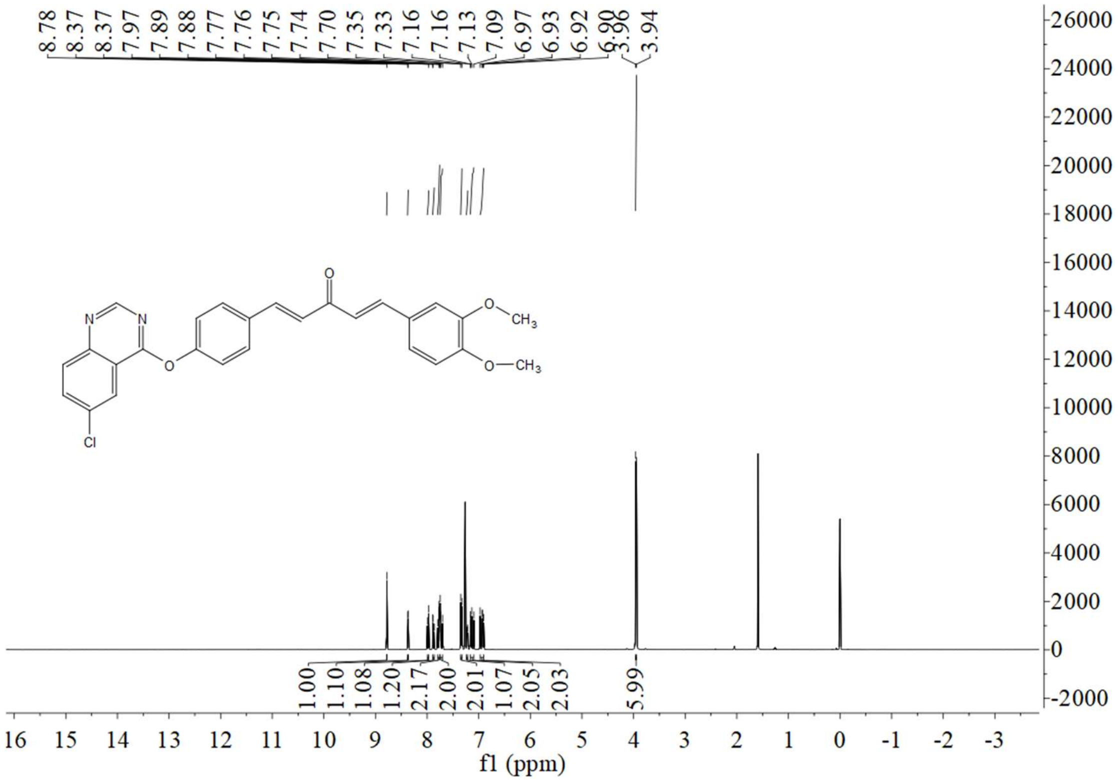

$^{1}$H NMR of compound **A15.**

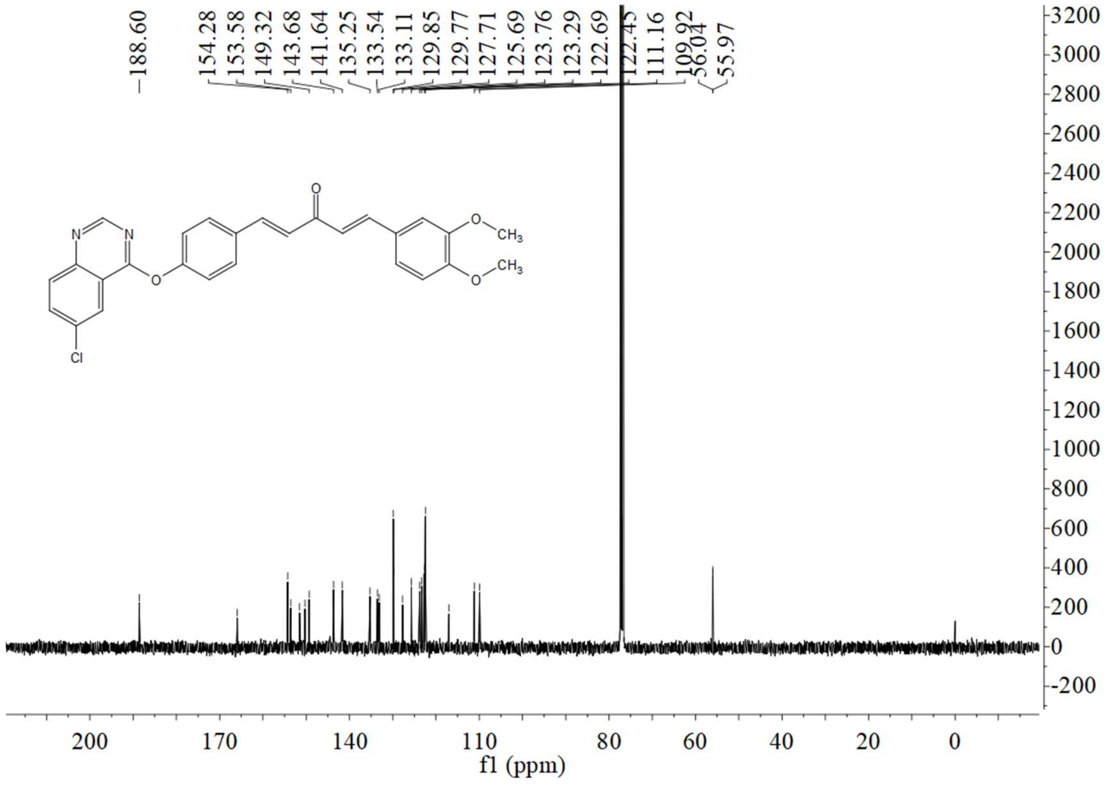

$^{13}$C NMR of compound **A15.**

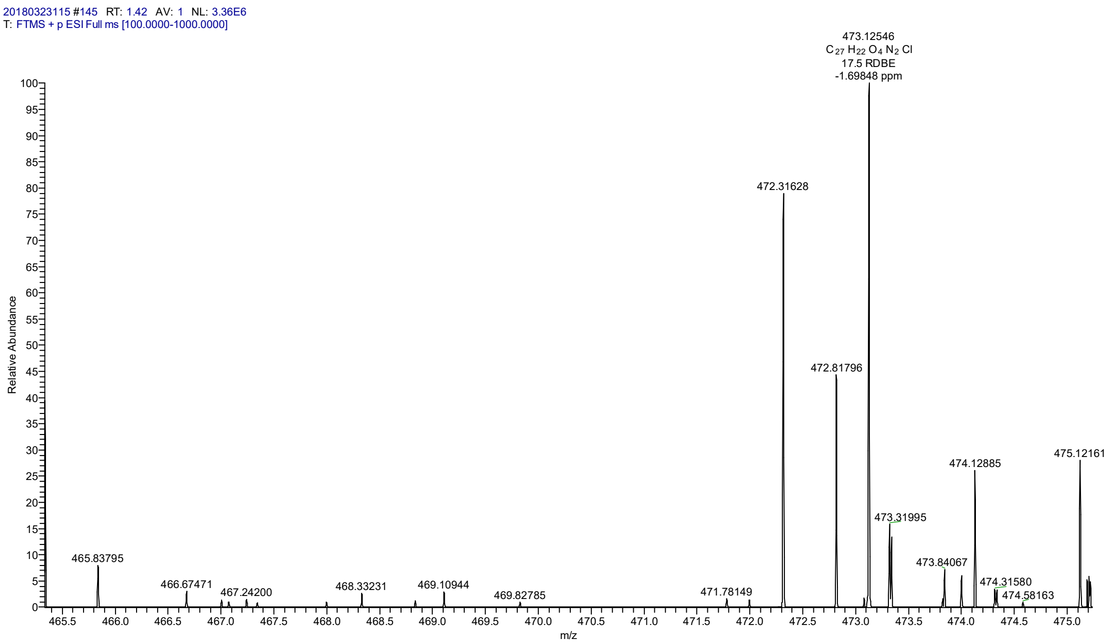


HRMS of compound **A15.**

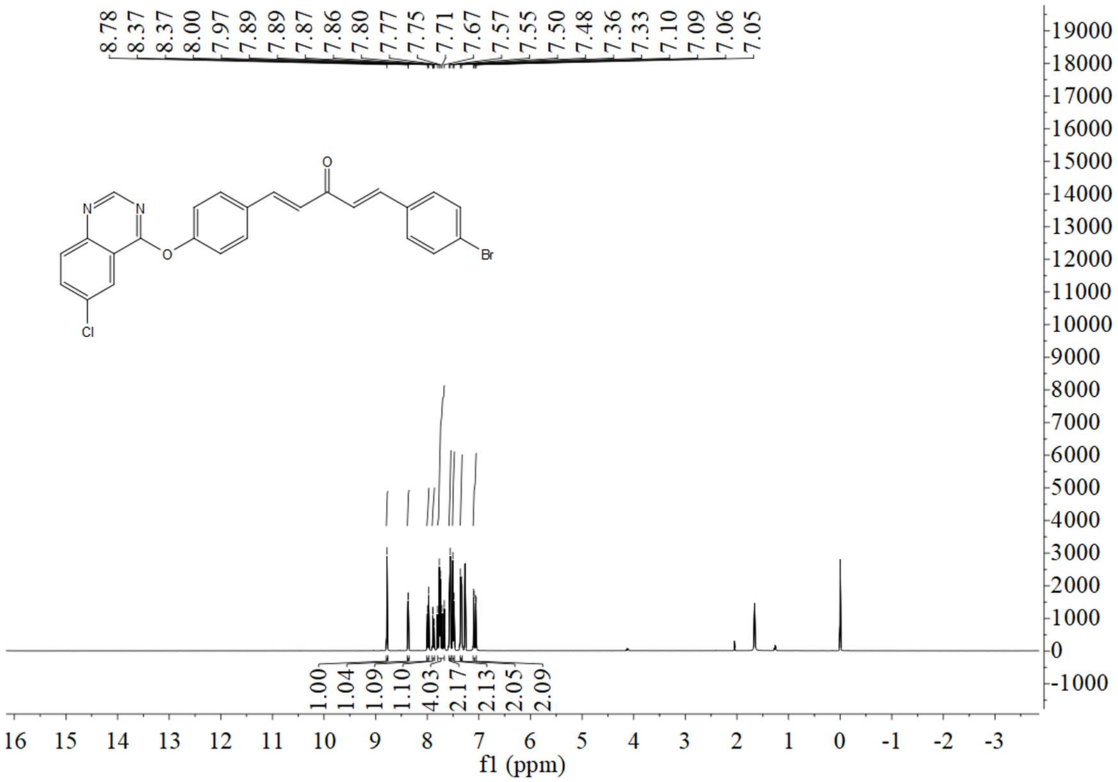


$^{1}$H NMR of compound **A16.**

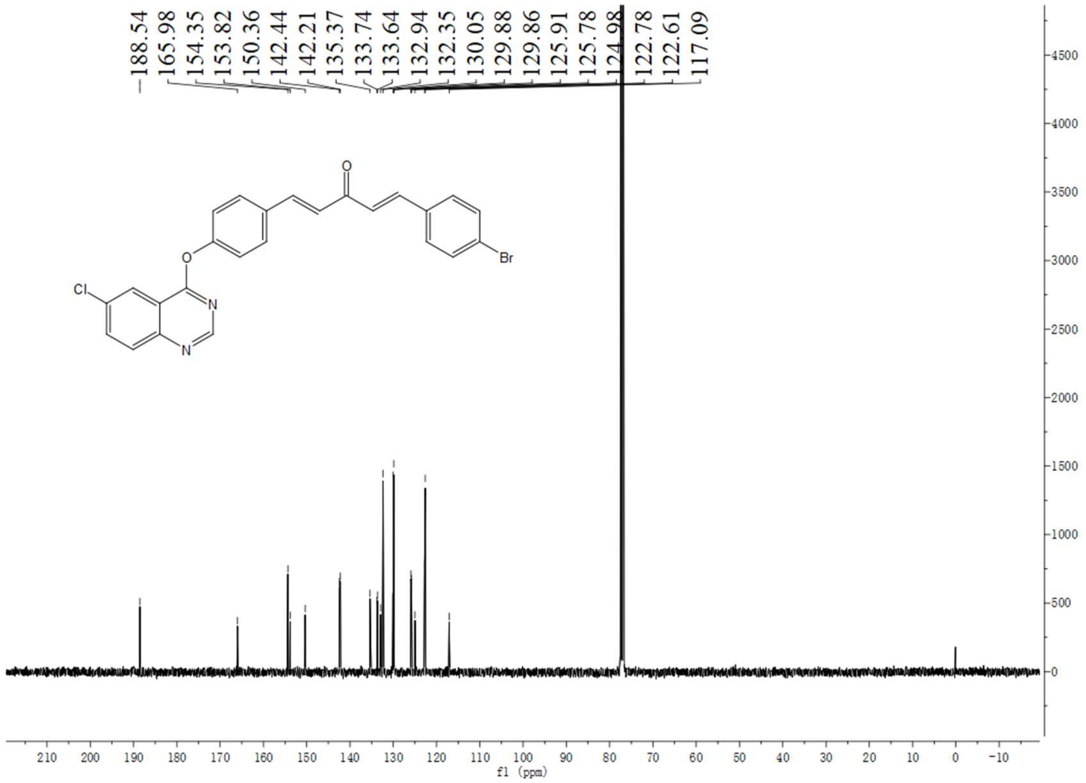


$^{13}$C NMR of compound **A16.**

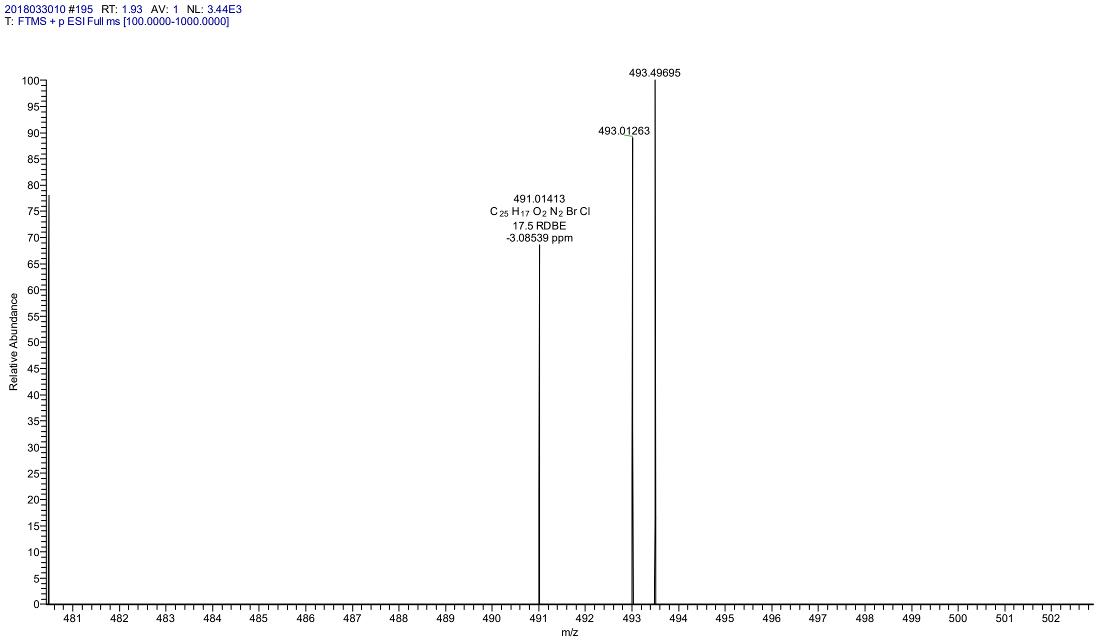


HRMS of compound **A16.**

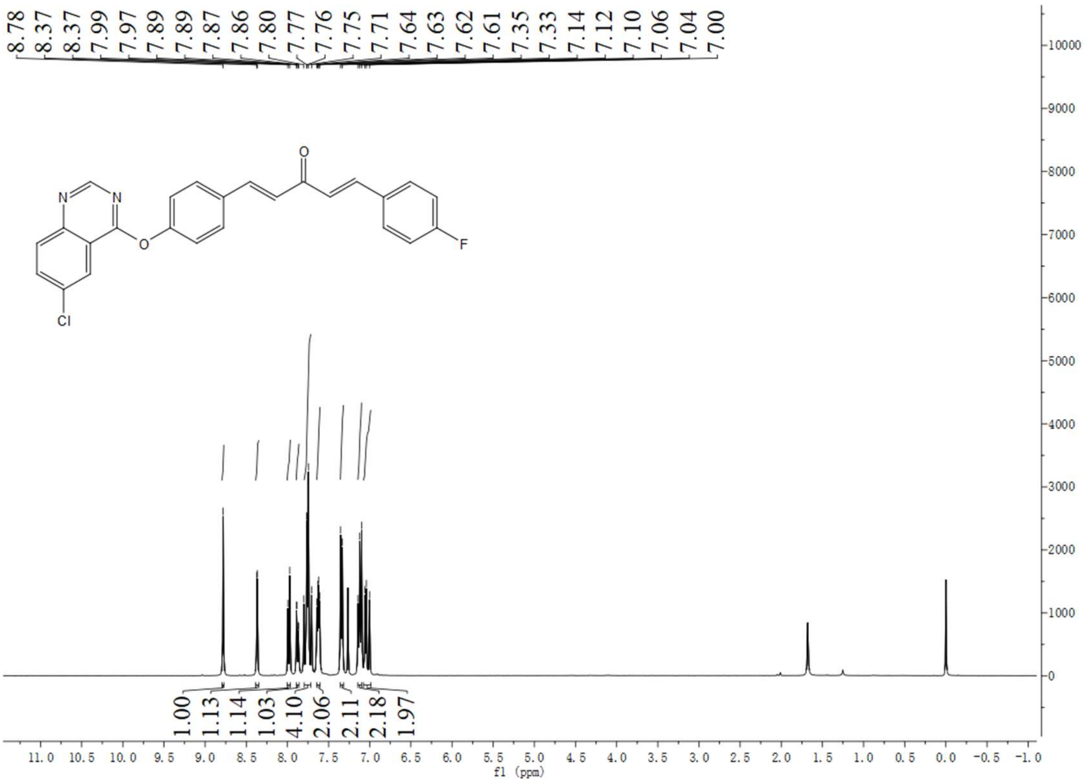

$^{1}$H NMR of compound **A17.**

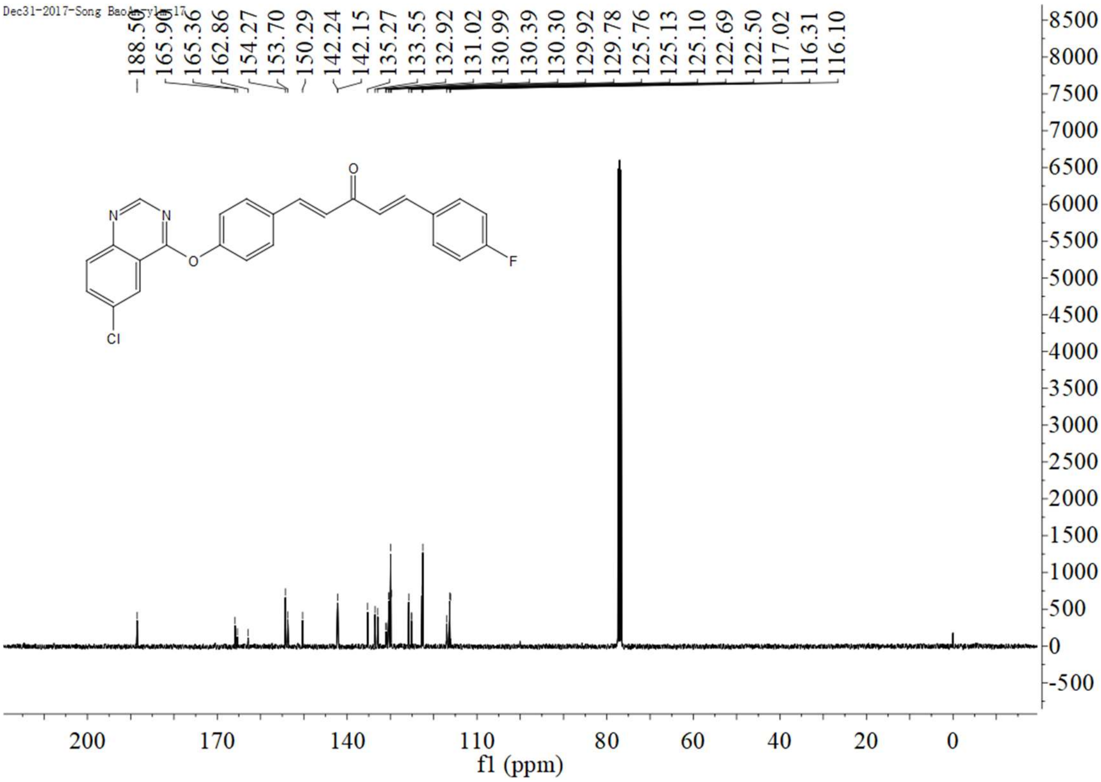

$^{13}$C NMR of compound **A17.**

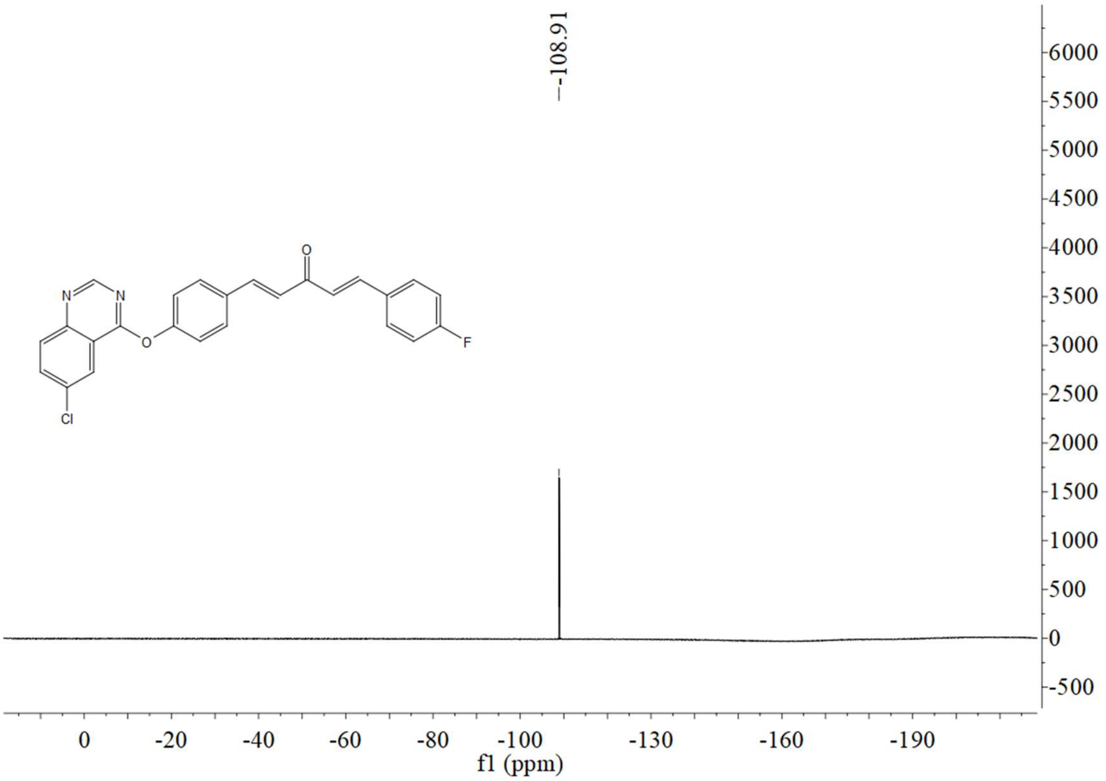


$^{19}$F NMR of compound **A17.**

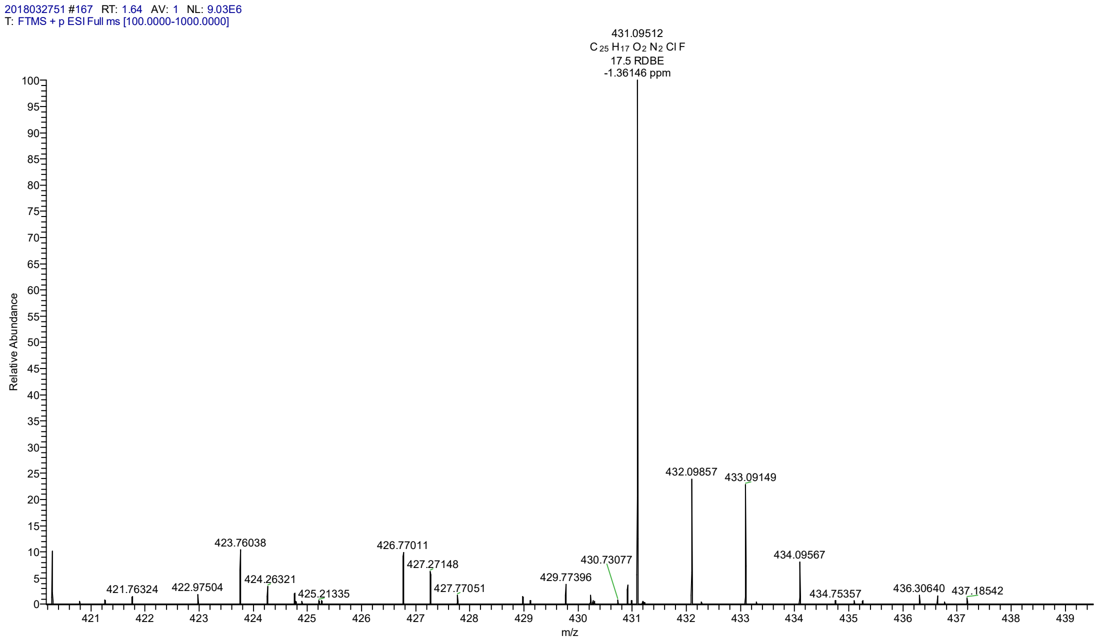


HRMS of compound **A17.**

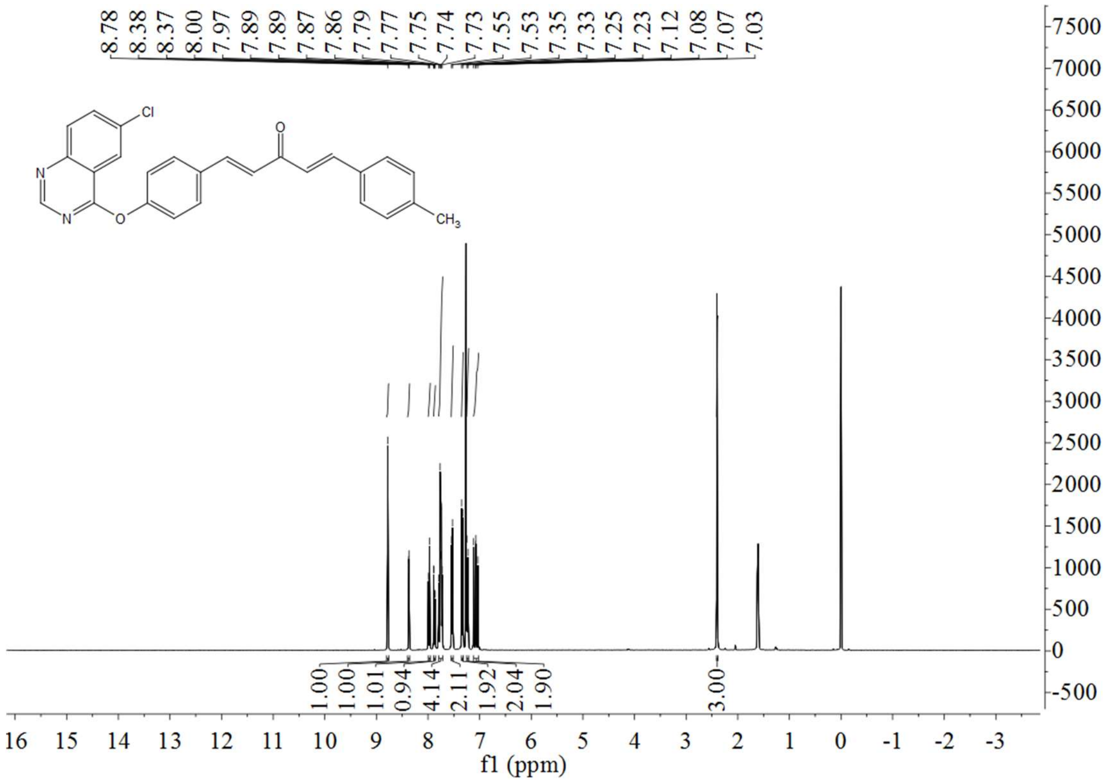

$^{1}$H NMR of compound **A18.**

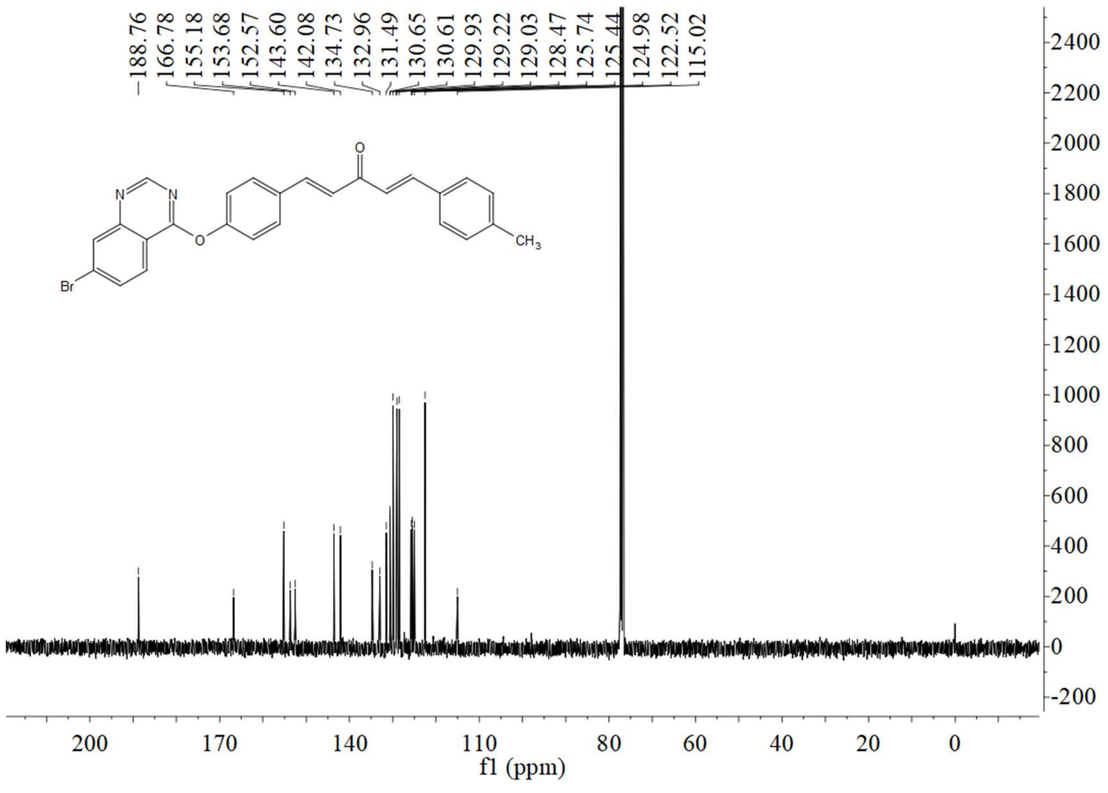

$^{13}$C NMR of compound **A18.**

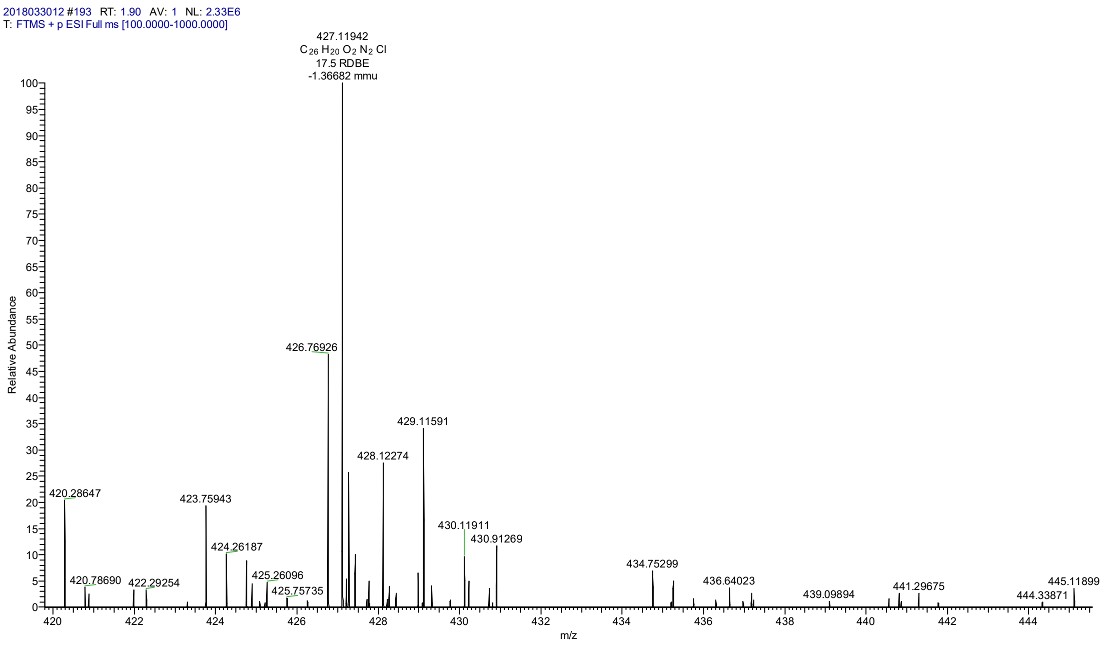


HRMS of compound **A18.**

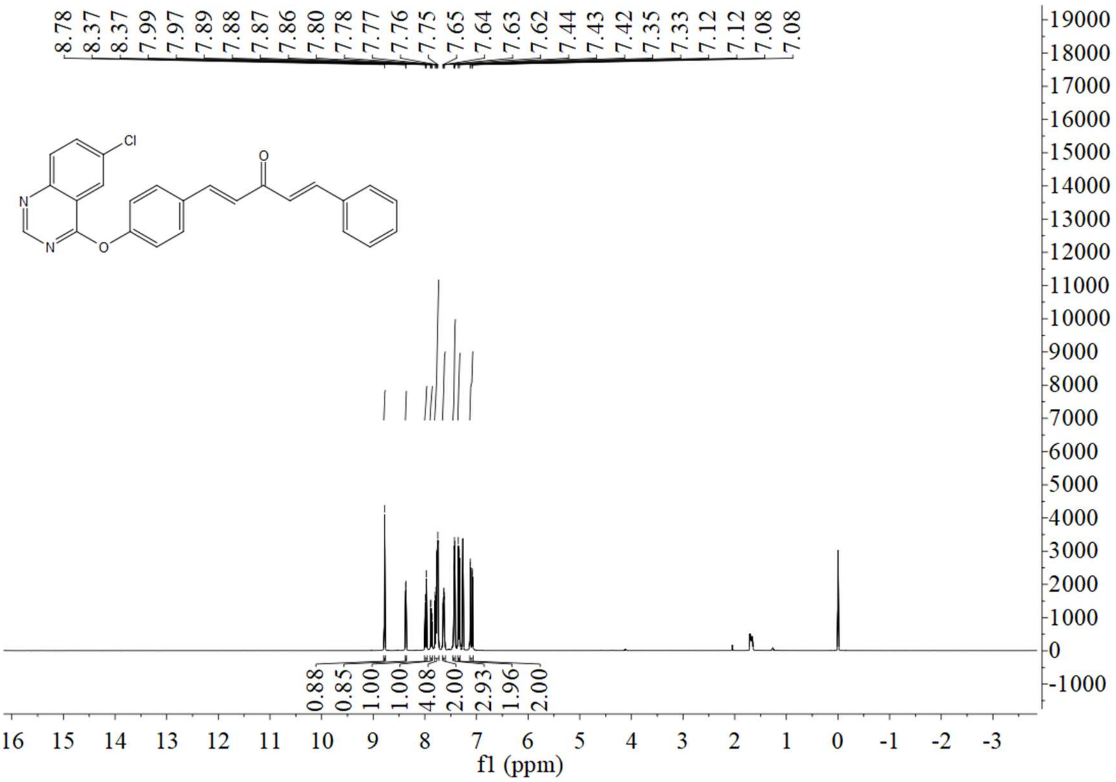


$^{1}$H NMR of compound **A19.**

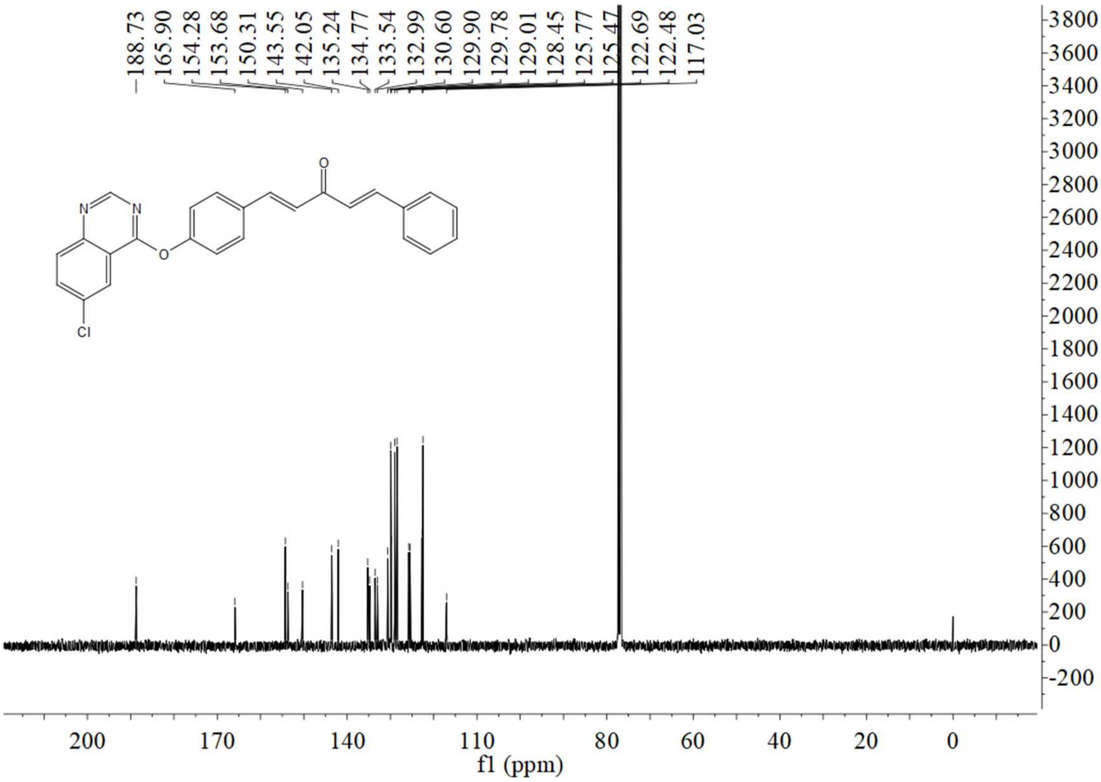


$^{13}C$ NMR of compound **A19.**

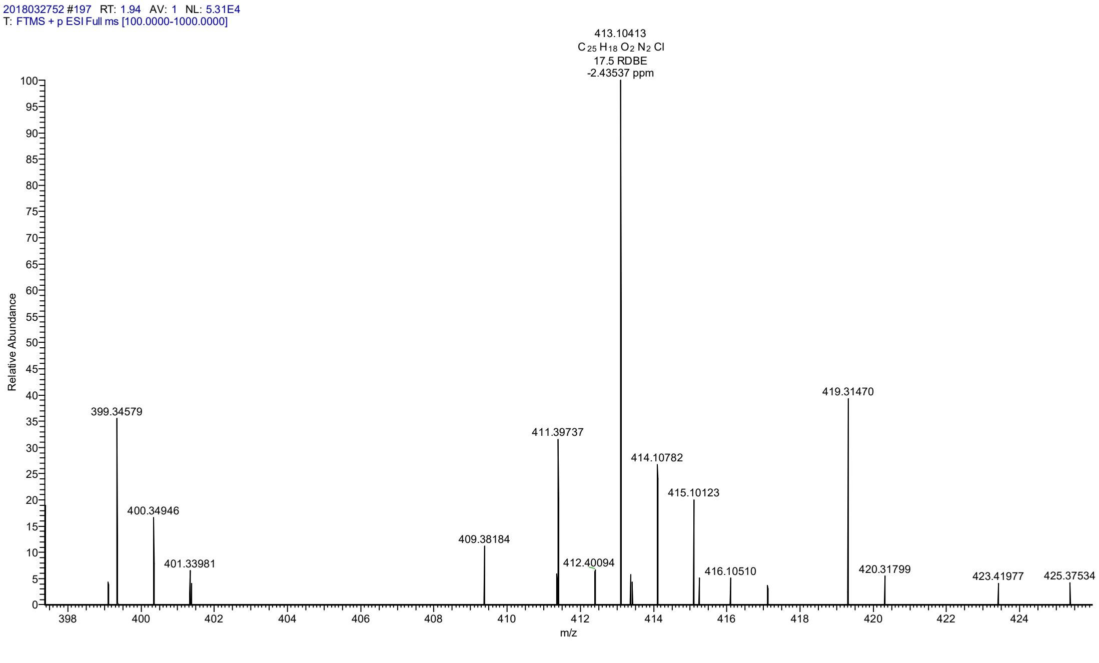


HRMS of compound **A19.**

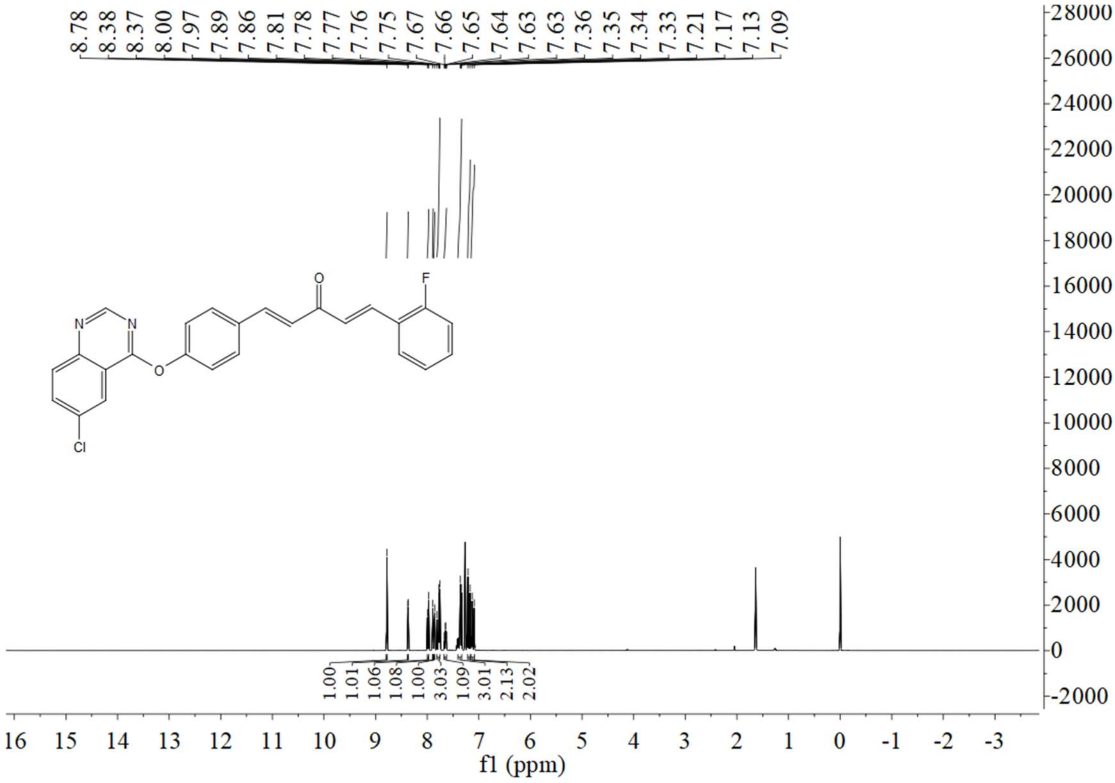

$^{1}$H NMR of compound **A20.**

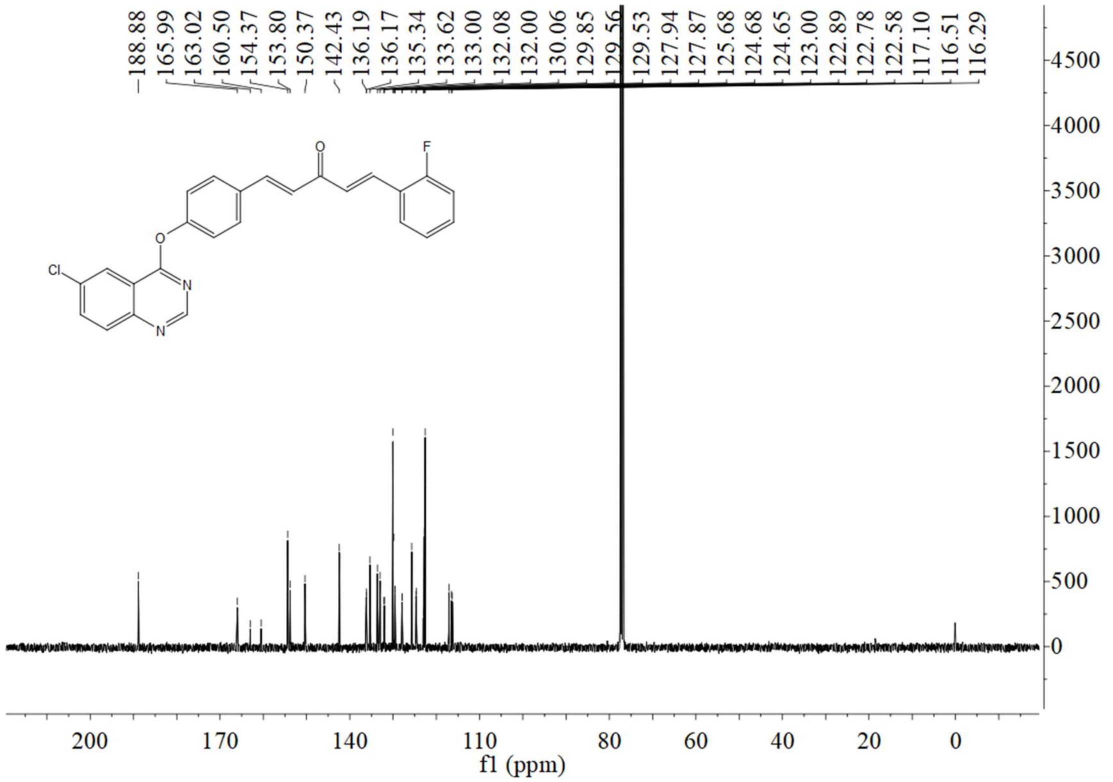

$^{13}$C NMR of compound **A20.**

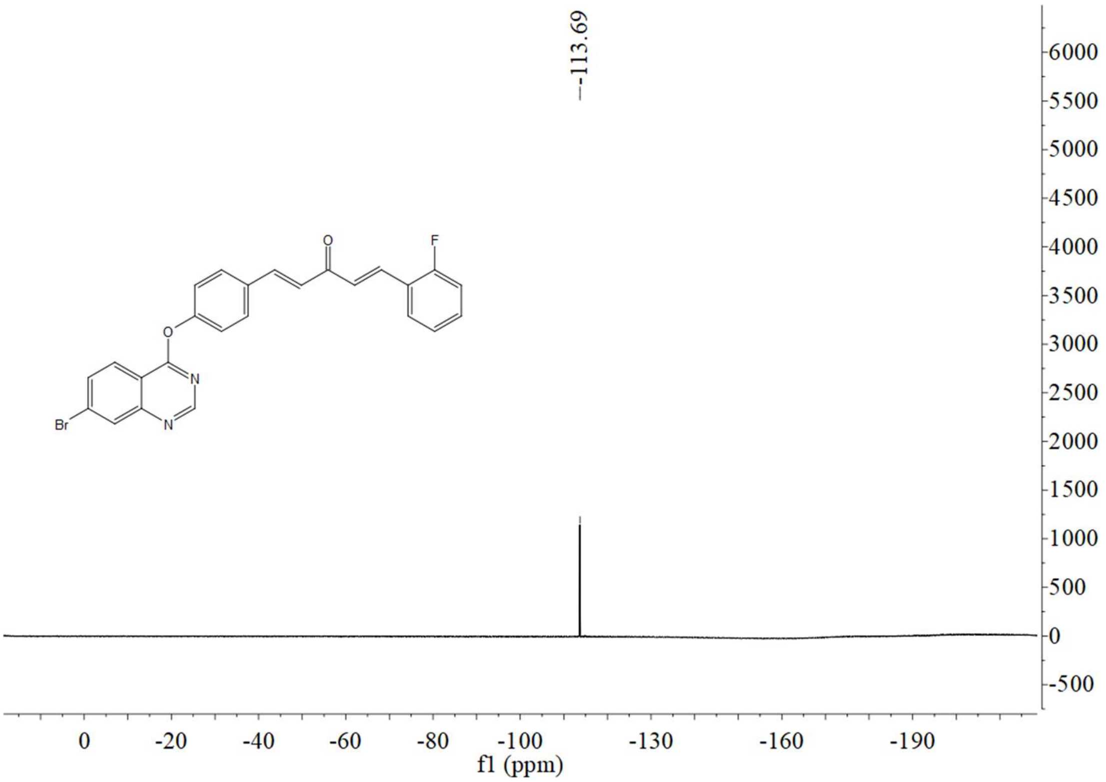


$^{19}$FNMR of compound **A20.**

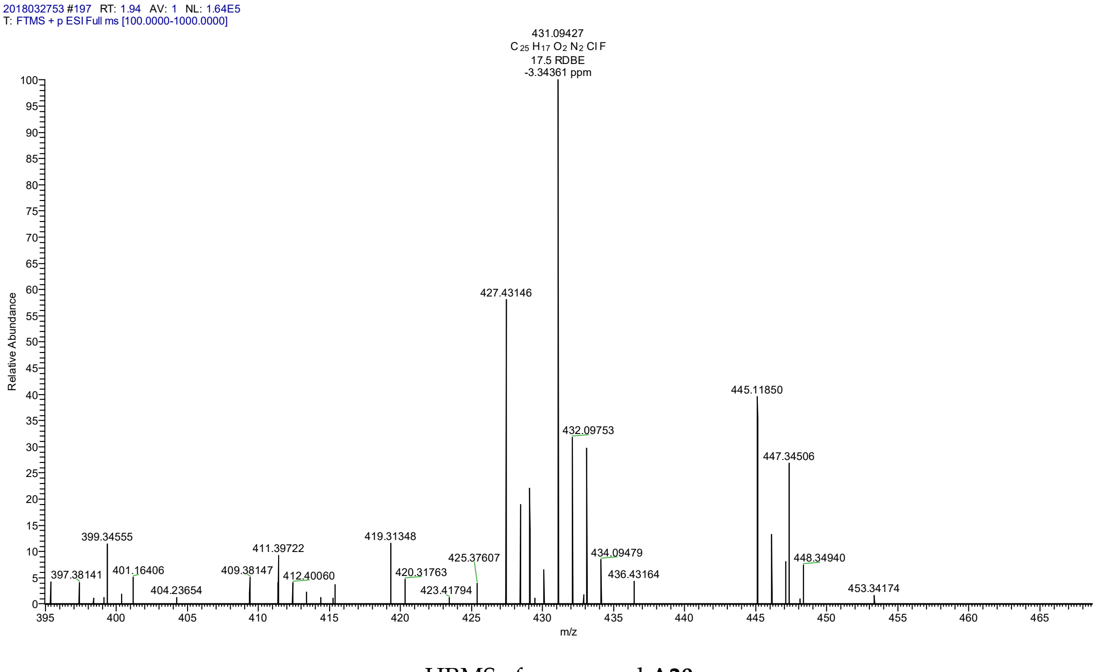


HRMS of compound **A20.**
